# Ring Opening Copolymerization of Boron-Containing Anhydride with Epoxides as a Controlled Platform to Functional Polyesters

**DOI:** 10.1021/jacs.3c03261

**Published:** 2023-06-13

**Authors:** Fernando Vidal, Sevven Smith, Charlotte K. Williams

**Affiliations:** Department of Chemistry, Chemical Research Laboratory, University of Oxford, 12 Mansfield Road, Oxford OX1 3TA, U.K.

## Abstract

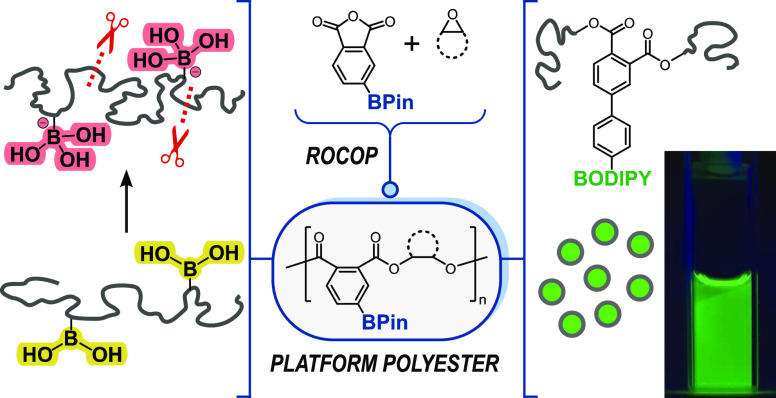

Boron-functionalized polymers are used in opto-electronics, biology, and medicine. Methods to produce boron-functionalized and degradable polyesters remain exceedingly rare but relevant where (bio)dissipation is required, for example, in self-assembled nanostructures, dynamic polymer networks, and bio-imaging. Here, a boronic ester-phthalic anhydride and various epoxides (cyclohexene oxide, vinyl-cyclohexene oxide, propene oxide, allyl glycidyl ether) undergo controlled ring-opening copolymerization (ROCOP), catalyzed by organometallic complexes [Zn(II)Mg(II) or Al(III)K(I)] or a phosphazene organobase. The polymerizations are well controlled allowing for the modulation of the polyester structures (e.g., by epoxide selection, AB, or ABA blocks), molar masses (9.4 < *M*_n_ < 40 kg/mol), and uptake of boron functionalities (esters, acids, “ates”, boroxines, and fluorescent groups) in the polymer. The boronic ester-functionalized polymers are amorphous, with high glass transition temperatures (81 < *T*_g_ < 224 °C) and good thermal stability (285 < *T*_d_ < 322 °C). The boronic ester-polyesters are deprotected to yield boronic acid- and borate-polyesters; the ionic polymers are water soluble and degradable under alkaline conditions. Using a hydrophilic macro-initiator in alternating epoxide/anhydride ROCOP, and lactone ring opening polymerization, produces amphiphilic AB and ABC copolyesters. Alternatively, the boron-functionalities are subjected to Pd(II)-catalyzed cross-couplings to install fluorescent groups (BODIPY). The utility of this new monomer as a platform to construct specialized polyesters materials is exemplified here in the synthesis of fluorescent spherical nanoparticles that self-assemble in water (*D*_h_ = 40 nm). The selective copolymerization, variable structural composition, and adjustable boron loading represent a versatile technology for future explorations of degradable, well-defined, and functional polymers.

## Introduction

Boron-functionalized polymers benefit from special properties due to the control over boron’s molecular orbital energies, Lewis and Bronsted acidity.^1−3^ In addition, boronic acids or esters confer dynamic, stimuli-responsive, and self-healing properties to materials, e.g., by transesterification reactions with aliphatic 1,2-, 1,3-diols or aromatic catechols.^4−6^ They are also well-known in C–C bond formations by metal catalyzed cross-couplings, accessing more complex pendant substituents.^7,8^ These features result in polymers that contain boronic esters/acids/borates being “synthons” for optoelectronics,^9,10^ (bio-)chemical sensors,^11^ drug delivery,^12,13^ cellular imaging,^14,15^ and vitrimers.^16−20^

Exploiting their properties requires efficient and precise synthetic methods. There are two main approaches: direct polymerization of boron-containing monomers or post-polymerization installation of the organoboranes onto polymer backbones.^21^ The former typically yields boronic ester/acid-hydrocarbon polymers by polymerization of boron-vinyl monomers, usually boron-styrene or *N*-phenylacrylamide.^22^ These vinyl monomers are polymerized either by free or controlled radical polymerizations, including reversible addition–fragmentation chain-transfer, RAFT,^23,24^ and atom-transfer radical polymerization, ATRP.^25^ Recently, more sophisticated structures have been obtained by olefin addition polymerization of allenyl- or vinylboronate esters^26−29^ or by ring-opening metathesis polymerization of 1,1-bis(boryl)cyclohexene.^30^ Critically, all these routes furnish hydrocarbon polymer backbones which may complicate any future recycling and usually prevent degradation. Many future materials applications, e.g., in resins or medicine, would benefit from boron-containing degradable polymers with options to manage end-of-life fates in organisms and the environment.

**Scheme 1:**
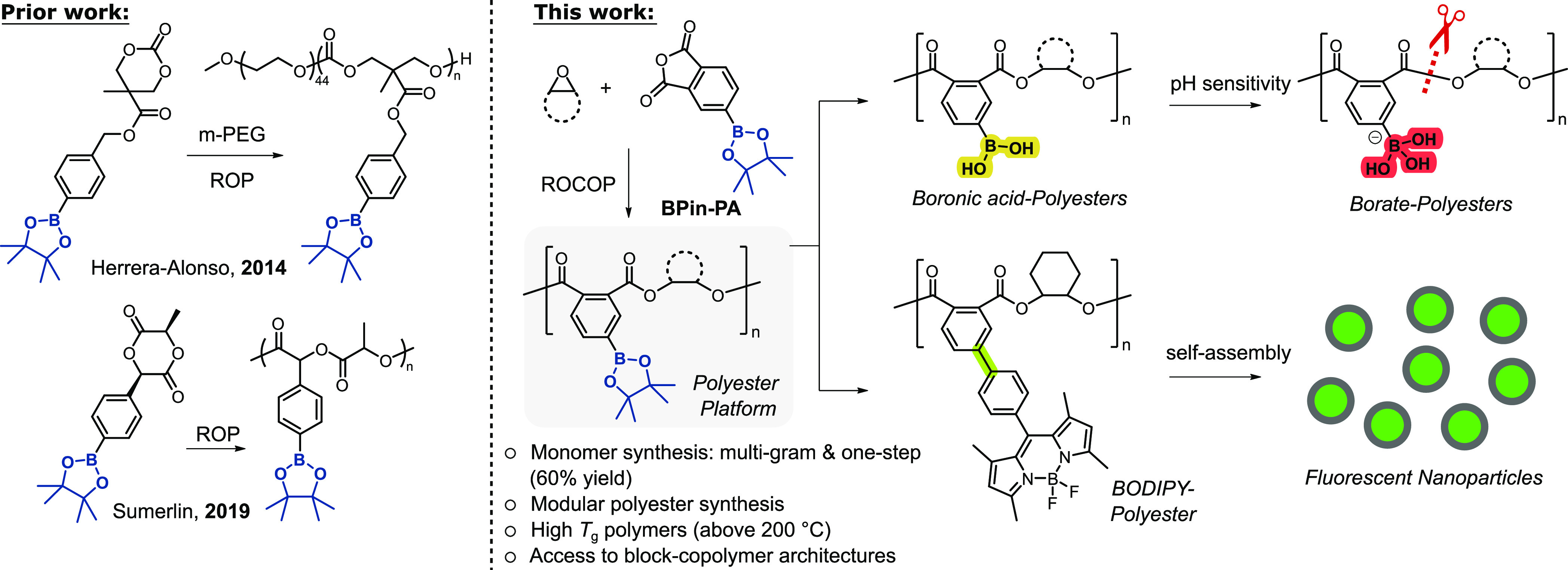
Boron-Containing Polyesters and Carbonates; Left: Prior Work; Right: An Overview of Boron-Polyesters and Block Polyesters in This Work

Polyesters are attractive degradable polymers since the ester linkages are susceptible to acid/base or enzymatic catalyzed hydrolyses, useful for chemical recycling to monomers.^31,32^ In some cases, the ester linkages also confer metabolic pathways for biodegradation or composting.^33−38^ Despite this enticing prospect, very few oxygenated polymers containing boronic ester/acid-units have been reported; some examples were accessed by post-polymerization modification of vinyl-containing polyesters via hydroboration.^39,40^ So far, the direct polymerization of boron-containing monomers was limited to the ring-opening polymerization (ROP) of a boron functionalised cyclic carbonate or lactone (Scheme 1, left). Specifically, Herrera-Alonso and co-workers prepared amphiphilic polycarbonates featuring pendant boronic esters and acids; these polymer nanoparticles self-assembled in water and displayed both pH and oxidative sensitivity.^41−43^ Later, Sumerlin and co-workers synthesized polylactide functionalized with boronic esters and acids, which showed potential to be employed in further derivatization reactions.^44^ The application of these materials as biocompatible and degradable drug nanocarriers exemplifies the importance of developing well-defined methods to access boron-functionalized polyesters.^45^

An alternative controlled route to polyesters is the catalyzed ring-opening copolymerization (ROCOP) of epoxides and cyclic anhydrides. It delivers aromatic, aliphatic and alkene functionalized polyesters starting from a wide range of commercial and bio-based monomers.^46−48^ It is particularly effective for functionalized monomers since the polymerization thermodynamics are favorable obviating the low-ring strain that often limits functionalized-heterocycle ROP. Another opportunity is to couple it with CO_2_/epoxide ROCOP and/or cyclic ester/carbonate ROP, using switchable catalysis, to produce block sequence selective copolymers.^49,50^ Using these switchable methologies, epoxide/anhydride ROCOP blocks were combined with other polymers to furnish thermoplastics,^51,52^ elastomers,^53,54^ pressure sensitive adhesives,^55^ hydrolysable surfactants, and amphiphile nanoparticles,^56^ and additives to toughen bio-based plastics like PLA.^57^

We reasoned that the significant co-monomer diversity accessible in epoxide/anhydride ROCOP might allow access to various boron-polyesters and offer a route to broaden their available functionalities and properties. To this end, a pinacol boronic ester-phthalic anhydride (BPin-PA) monomer was targeted since its formation was reported as feasible starting from commercial phthalic anhydride (PA).^58^ Many epoxide/anhydride ROCOP catalysts are “benchmarked” using PA, and hence, various efficient catalysts were evaluated for BPin-PA/epoxide ROCOP with a view to achieve control over polymer architectures and the extent of boron-functionalization (Scheme 1, right). Four different epoxides were selected for the ROCOP: two are bicyclic, resulting in rigid backbone chemistries, cyclohexene oxide (CHO), and vinyl cyclohexene oxide (vCHO), and two are alkylene oxides, yielding more flexible backbones, propylene oxide (PO), and allyl glycidyl ether (AGE). The second goal was to investigate the reactivity of the boronic ester (polyester-BPin), particularly toward deprotection to yield boronic acids [polyester-B(OH)_2_] and boronate salts [polyester-B(OH)_3_^–^] which might afford water solubility and degradability. As epoxide/anhydride ROCOP is a highly controlled polymerization, exploiting it to incorporate boron functionalized polyesters within block structures and amphiphiles is another objective. Finally, the reactivity −BPin groups in Suzuki–Miyaura cross-coupling offers another opportunity to fine-tune properties and performance of these polyesters. To illustrate this post-polymerization modification, fluorescent BODIPY-bearing amphiphilic poly(ether)-*block*-poly(ester) nanoparticles were explored. The overall aim was to develop efficient, well-controlled polyester syntheses and transformations relevant to future materials as vitrimers, in drug-delivery, imaging, and tissue engineering.^59−61^

## Results and Discussion

### BPin-PA and Epoxide Ring Opening Copolymerization

Phthalic anhydride functionalized with a pinacol boronic ester (BPin-PA) was synthesized from commercially available bis(pinacolato)diboron and 4-bromo-phathalic anhydride using Pd(0)-catalyzed cross-coupling (see the Supporting Information for details). The analytically pure BPin-PA monomer was obtained, after consecutive recrystallization and sublimation, as a white crystalline powder in a multi-gram scale and in moderate yield (60%) (Figures S1–S4).

The ROCOP of BPin-PA with various epoxides was investigated using two different organometallic catalysts, heterodinuclear complexes [L^1^ZnMg(C_6_F_5_)_2_] (**ZnMg**) or [L^2^AlK(Cp)(Et)] (**AlK**) (Table 1 for structures). These catalysts were selected as they showed high activity, end-group selectivity, and control, combined with low loading tolerance, in prior heterocumulene/epoxide ROCOP.^52,53,62^ Both metal-based catalysts feature organometallic co-ligands (bispentafluorophenyl and ethyl/cyclopentadienyl for **ZnMg** and **AlK**, respectively) that react rapidly and irreversibly with diols, e.g., 1,4-benzenedimethanol (BDM), to form the metal-alkoxide initiators in situ. These catalysts show better control over initiation and subsequent chain end-group chemistry than many other ROCOP catalyst systems which often feature several initiators.^52^ The organometallic catalysts are easily removed, after polymerizations, by precipitation and filtration (silica) producing colorless polymers.

**Table 1:**
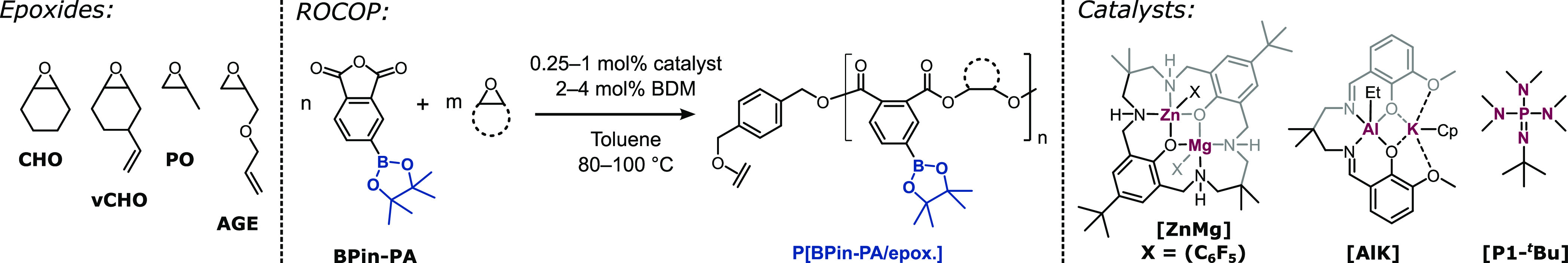
BPin-PA and Epoxide ROCOP^a^

#	polymer	epoxide	catalyst	[BPin-PA] equiv	[BDM] equiv	[epox.] equiv	time (h)	temp. (°C)	conv.^b^ (%)	*M*_n, theo_^c^ (kDa)	*M*_n, GPC_^d^(kDa)	*Đ*^d^	*T*_g_^e^	*T*_d_^f^
1	[BPin-PA/vCHO]	vCHO	[ZnMg]	100	4	400	2	80	100	10.1	11.8	1.08	199	301
2	[BPin-PA/vCHO]	vCHO	[AlK]	400	4	2000	1.5	100	100	40.0	41.5	1.16	212	293
3	[BPin-PA/CHO]	CHO	[ZnMg]	100	4	400	2	80	100	9.4	8.5	1.22	213	322
4	[BPin-PA/CHO]	CHO	[AlK]	400	4	2000	1.5	100	100	37.4	10.6	1.40	224	285
5	[BPin-PA/PO]	PO	P1-^*t*^Bu	100	2	150	24	60	100	16.7	21.1	1.04	139	300
6	[BPin-PA/AGE]	AGE	P1-^*t*^Bu	100	2	150	48	60	>99	18.6	16.5	1.08	81	294

a [BPin-PA]_0_ = 1.00 M in toluene; full experimental details in the Supporting Information.

b Conversion of anhydride as determined by ^1^H NMR spectrocopy.

c *M*_n, theo_ = MW(BPin-PA + epox.) × [BPin-PA]/[cat.] × [ZnMg]/[CTA] × conv (%) + MW of chain-end groups.

d Determined by gel permeation chromatography in THF, except entries 3 and 4 which were run in CHCl_3_, instruments calibrated against polystyrene standards.

e Obtained from the second heating scan by differential scanning calorimetry (10 °C·min^–1^).

f Obtained by thermogravimetric analysis (10 °C·min^–1^).

First, **ZnMg** was tested for BPin-PA/vCHO ROCOP yielding poly(pinacolboronate phthalate-*alt*-vinylcyclohexylene oxide), P(BPin-PA/vCHO). Polymerizations were conducted using 1:4:100:400 loadings of [**ZnMg**]:[BDM]:[BPin-PA]:[vCHO] (i.e., 1 mol % catalyst vs anhydride), in toluene ([BPin-PA]_0_ = 1.00 M), at 80 °C—conditions which were effective for PA/vCHO ROCOP (Table 1, #1). Analysis of reaction aliquots by ^1^H NMR spectroscopy indicated quantitative anhydride conversion to fully alternating polyester within 2 h, as evidenced by the disappearance of the aromatic resonances of BPin-PA (CDCl_3_: 8.43, 8.30, and 7.98 ppm) and the appearance of new broad signals for the aromatic polyester resonances (CDCl_3_: 8.09, 7.92, and 7.68 ppm).

The **AlK** catalyst is among the fastest catalysts reported for CHO/PA ROCOP and hence was tested under more demanding conditions (Table 1, # 2).^52^ These conditions include lower catalyst loading (400 equiv or 0.25 mol % vs anhydride), higher epoxide loading (2000 equiv), and higher temperature (100 °C). The polymerization proceeded to complete anhydride consumption within 1.5 h, resulting in a turnover-frequency (TOF) of 267 h^–1^. The polymer, P(BPin-PA/vCHO), showed a high molar mass, *M*_n_ = 41.5 kg/mol, and narrow dispersity (*D̵* = 1.16). It also showed high selectivity for ester linkages (>99%), and extending the reaction time beyond complete BPin-PA conversion did not form any ether linkages despite the large excess of epoxide.

**Figure 1:**
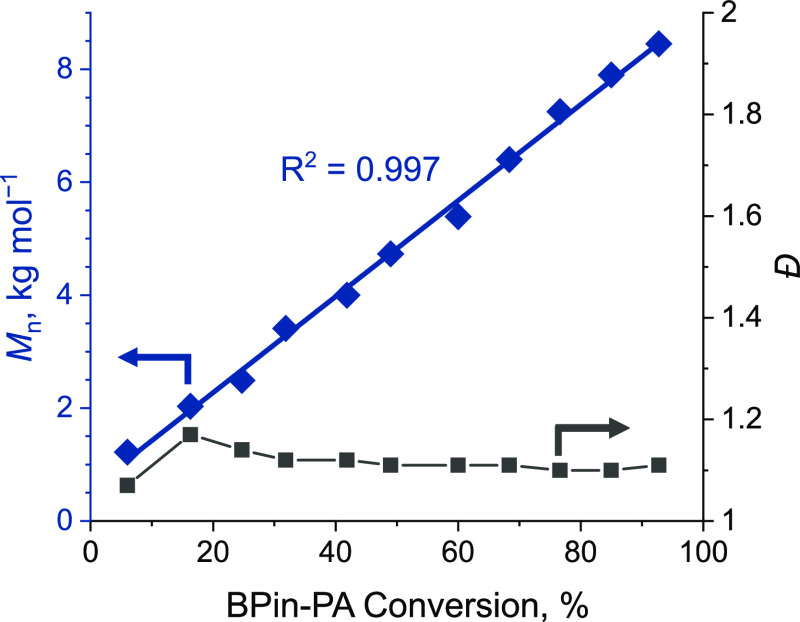
(a) Plot of P(BPin-PA/vCHO) molar mass, *M*_n_ (blue diamonds), and dispersity, *Đ* (black squares) vs BPin-PA conversion as catalyzed by **ZnMg** ([BPin-PA]_0_ = 0.76 M, [BPinPA]:[**ZnMg**]:[BDM] ratio of 100:1:4, neat, 80 °C).

**Figure 2:**
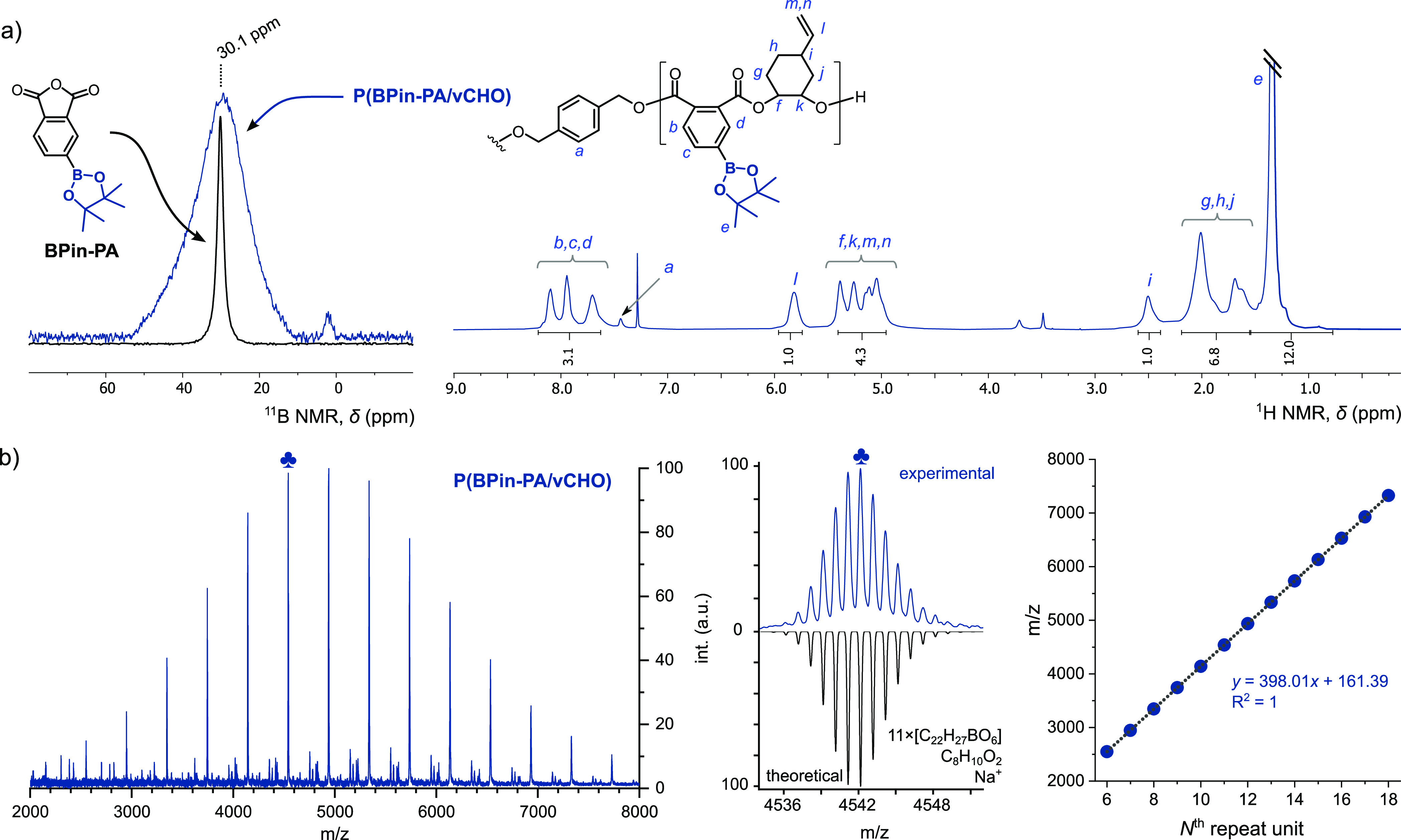
Characterization data for boron-functionalized polyesters. (a) Left: ^11^B NMR, and right: ^1^H NMR spectra (CDCl_3_, 25 °C) of P(BPin-PA/vCHO) (blue line) and BPin-PA (black line). (b) Left: MALDI-TOF spectrum of P(BPin-PA/vCHO) (low molar mass aliquot) using **ZnMg;** middle: comparison of the experimental and theoretical isotope distributions for the peak at 4542 *m*/*z*, 11-mer; and right: plot of *m*/*z* vs *N*th repeat unit. MW_theo_ of the *n*th repeat unit (C_22_H_27_BO_6_) = 398.19 g·mol^–1^; MW_theo_ of the end group (C_8_H_10_O_2_) + Na^+^ = 161.06 g·mol^–1^.

Both organometallic catalysts showed good polymerization with the polyesters showing molar masses close to predicted values and exhibiting monomodal, narrow disperity distributions (Table 1, #1, 2). Using **ZnMg**, the reaction aliquot analyses showed linear increases to polyester molar mass with anhydride conversion, with narrow dispersity (*Đ* = 1.07–1.17, Figure 1). Analysis of the polyester using matrix-assisted laser desorption ionization time-of-flight (MALDI-TOF) mass spectrometry confirmed the selective formation of telechelic α,ω-dihydroxy polyester (Figure 2b). It also corroborated the retention of the pinacol boronic ester moieties attached to the polyester. The experimentally determined polymer repeat unit mass was 398.01 g·mol^–1^ (obtained from the gradient of *m*/*z* vs *n*th repeat unit) which matched closely the theoretical value for BPin-PA/vCHO (398.19 g·mol^–1^). Moreover, modeling of the isotopic distribution for the 11th-mer [molecular formula = (C_22_H_27_BO_6_)_11_ for the polyester chain, C_8_H_10_O_2_ for the chain-end, and Na^+^] exactly matched the experimental peaks centered at 4542 *m*/*z*. The relative integrals of the resonances, in the ^1^H NMR spectrum, were also consistent with a perfectly alternating polyester (i.e. BPin-PA/vCHO). This was most clearly exemplified by the resonances assigned to the −BPin at 1.32 ppm (methyl groups, 12 H) and the vinyl resonances at 5.80 ppm (methine groups, 1 H) (Figure 2a). The pinacol boronic ester was also confirmed by the ^11^B NMR spectrum, in which resonances for monomer and polymer both appear at 30.1 ppm.

Next, BPin-PA was polymerized, using **ZnMg** under otherwise identical conditions, with CHO to produce poly(pinacolboronate phthalate-*alt*-cyclohexylene oxide), P(BPin-PA/CHO) (*M*_n_ = 8.5 kDa, *D̵* = 1.22) (Table 1, # 3). The polymerization was well controlled, as evidenced by the linear increase of polyester P(BPin/CHO) molar mass vs anhydride conversion and retention of narrow dispersity distributions (*D̵* = 1.10–1.13) (Figure S15). Using the **AlK** catalyst resulted in faster polymerization to P(BPin-PA/CHO), although the molar mass distributions were slightly broader perhaps due to low quantities of residual impurities in the epoxide (Table 1, # 4).

**Figure 3:**
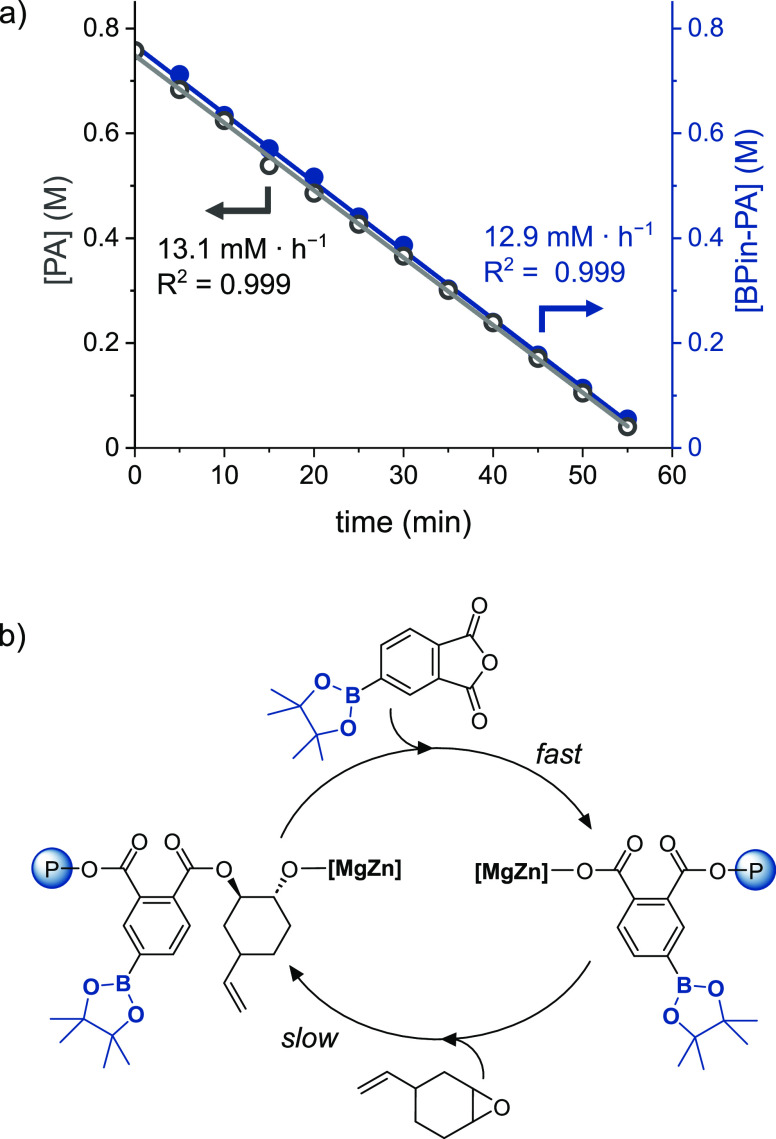
(a) Plots of anhydride concentrations vs time for the ROCOP of PA/vCHO (empty circles) and BPin-PA/vCHO (blue circles) using **ZnMg** (neat epoxide, 80 °C). Data are fit with straight lines and gradients are *k*_obs_ values. (b) Proposed mechanism for BPin-PA/vCHO ROCOP using **ZnMg** (NB: ROCOP is regio-random but a single isomer is illustrated for clarity).

The high degree of polymerization control using **ZnMg** and **AlK** catalysts is significant since it confirms the innocence of the boron functionality (a weak Lewis acid) in the catalysis. The polymerization kinetics for BPin-PA/vCHO ROCOP were evaluated in neat epoxide, using **ZnMg** as the catalyst, with regular aliquot analysis by ^1^H NMR spectroscopy. Plotting anhydride conversion vs time showed a linear fit to the data (*R*^2^ = 0.999), indicating a zeroth-order rate dependence (Figure 3a). This result is fully consistent with investigations of vCHO/PA ROCOP using these organometallic catalysts.^52,53^ The mechanism is proposed to involve fast anhydride insertion into a metal-alkoxide intermediate and rate-limiting, epoxide ring-opening by a metal-carboxylate intermediate (Figure 3b).^63^ Importantly, the pseudo zeroth-order rate constants, *k*_obs_, for ROCOP using vCHO and either PA or BPin-PA were nearly identical (*k*_obs_ = 13.1 and 12.9 mM·h^–1^, respectively). The polymerization kinetics were also monitored for equivalent reactions using CHO; once again, the experimental rate constants for PA or BPin-PA ROCOP were very similar (*k*_obs_ = 13.2 and 14.1 mM·h^–1^, respectively, Figure S13). The **ZnMg** catalyst showed objectively high rates, resulting in TOF values of 102 and 114 h^–1^ for ROCOP of BPin-PA with vCHO and CHO, respectively ([BPin-PA]_0_ = 0.76 M, [BPinPA]:[**ZnMg**]:[BDM] ratio of 100:1:4, neat, 80 °C).

Next, BPin-PA and epoxide ROCOP was investigated using a commercial phosphazene organocatalyst, **P1-**^***t***^**Bu** (Table 1). The organophosphazene “superbase” is typically applied in anhydride/epoxide ROCOP with alcohols as initiators; in this case, the same diol (BDM) was employed. The phosphazene catalyst showed very good control in other epoxide/anhydride ROCOP and was particularly effective using alkylene oxides, e.g., propene oxide (PO) or allyl glycidyl epoxide (AGE).^64^ Here, polymerizations were conducted using 1:2:100:200, [**P1-**^***t***^**Bu**]:[BDM]:[BPin-PA]:[epoxide], at 60 °C, in toluene (Table 1, # 5–6). The polymerizations of BPin-PA/PO and BPin-PA/AGE proceeded slowly over 24 h to produce poly(pinacolboronate phthalate-*alt*-propylene oxide), P(BPin-PA/PO), and poly(pinacolboronate phthalate-*alt*-allyl glycidyl epoxide), P(BPin-PA/AGE). Both polyesters showed perfectly alternating structures, moderate molar masses (*M*_n_ = 21.1 and 16.5 kDa, respectively) and narrow dispersity (*D̵* = 1.04 and 1.08, respectively). Again, the boronic ester substituents remained intact after polymerization, as confirmed by spectroscopic analysis of the isolated polymers (Figures S16–S27). Accordingly, BPin-PA undergoes epoxide ROCOP with both organometallic **ZnMg, AlK**, and **P1-**^***t***^**Bu** catalysts.

Thermal characterization of the four new boronic ester-polyesters was undertaken using differential scanning calorimetry (DSC). All the polyesters are amorphous and show strikingly high glass transition temperatures (*T*_g_), whose values increased with the epoxide rigidity from 81, 139, 212, and 224 °C for P(BPin-PA/AGE), P(BPin-PA/PO), P(BPin-PA/vCHO), and P(BPin-PA/CHO), respectively (Figures S33 and S34). The *T*_g_ values are ∼70–90 °C higher than those for analogous polyesters containing PA, i.e., without the boronic ester (Table S2). Prior studies have also shown that the introduction of −BPin substituents to polystyrene result in ∼90–100 °C higher glass transition temperatures (*T*_g_ = 197–228 °C).^25,65,66^ In the polyester field, glass transition temperatures above 100 °C are quite rare and these values are exceptionally high.^46^ Nevertheless, the materials retain a reasonable processing temperature range with the on-set of thermal decomposition occurring at temperatures (*T*_d_) from 285 to 322 °C (Figures S35–S38).

### Boronic Acid-Polyesters

With a successful method to make poly(boronic ester)s (polyester-BPin) in hand, attention turned to their deprotection as a means to access diverse poly(boronic acid)s [polyester-B(OH)_2_]. One concern was that the typical conditions for boronic ester deprotection might also result in cleavage of the backbone polyester linkages. A common deprotection strategy reacts the boronic ester-polymer with an excess of a soluble boronic acid (i.e. a diol scavenging reagent) to drive the transesterification equilibrium toward the boronic acid-polymer. In this manner, polystyrenes and polycarbonates, featuring boronic ester substituents, were successfully transformed into the corresponding poly(boronic acid)s.^41,67^ Alternatively, a resin support bearing heterogeneous boronic acids was used to produce boronic acid-polystyrene and polylactide with simple purification steps.^23,44^ Another option is to exploit the volatility of methyl boronic acid (MBA) in the transesterification; this method has yielded boronic peptides and covalent organic networks.^68−70^ Overall, the last method is attractive since it was high yielding, economical, performed at room temperature, and involved simple purifications. Thus, the applicability of using MBA in transesterications with various polyester-BPin was investigated.

**Figure 4:**
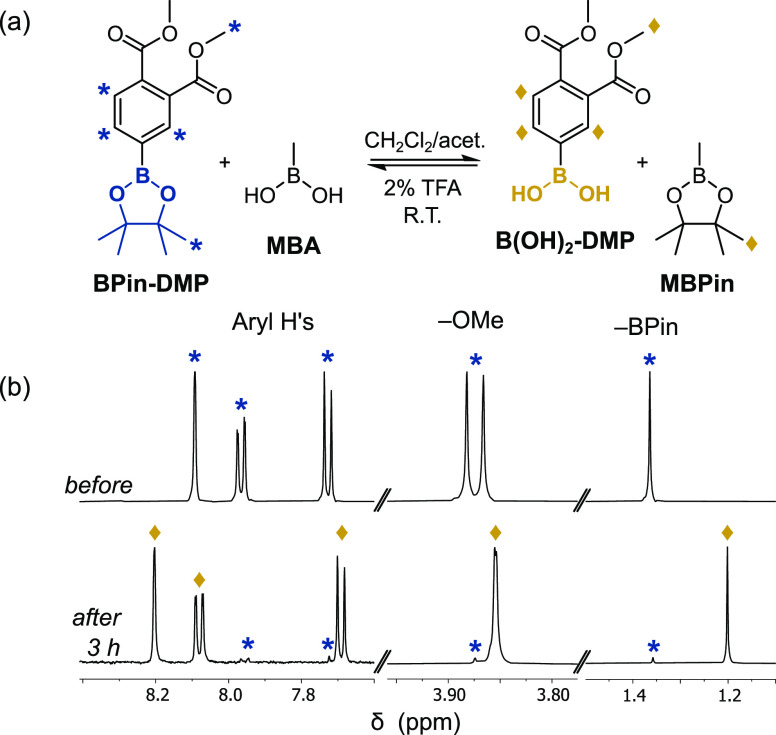
(a) Model reaction between BPin-DMP and MBA. (b) Sequential ^1^H NMR spectra (acetone-*d*_6_, 25 °C) of the reaction mixture before and 3 h after the addition of MBA (20 equiv), with TFA catalyst (2 vol %) at 25 °C.

First, a model study was conducted by reacting 4-BPin-dimethylphthalate (BPin-DMP), which served as a molecular analogue of the polyester chains, with excess MBA, at room temperature and with trifluoroacetic acid (TFA) as the catalyst (Figure 4a). Reaction aliquots were regularly analyzed, using ^1^H NMR spectroscopy, which indicated the formation of the boronic acid-dimethylphthalate [B(OH)_2_-DMP] (resonances: 8.22, 8.09; and 7.70 ppm). These changes were accompanied by the near total disappearance of the methyl resonances of the −BPin moiety (1.36 ppm) and evolution of signals for methyl pinacolboronic ester (MBPin, 1.20 ppm). The ^1^H NMR spectra indicated that reaction conversions were high (>88%) (Table S3). The conversions were rather insensitive to solvent, the amount of TFA or to the initial boronic ester concentration, although a significant increase in conversion, to 96%, was achieved by using a larger excess of MBA (20 equiv, Figure 4b). Purification of B(OH)_2_-DMP was straightforward with the removal of volatile molecules in vacuo and subsequent crystallization to obtain the pure product (acetone/hexane) as a white powder.

**Figure 5:**
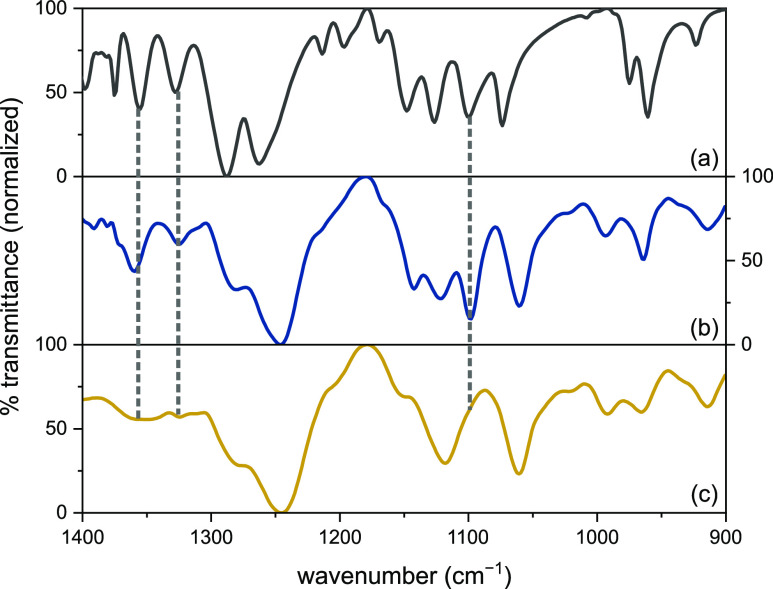
Selected region of the FT-IR spectra of (a) BPin-DMP, (b) P[BPin-PA/vCHO], and (c) P[B(OH)_2_-PA/vCHO]. The dotted lines show the loss of −BPin vibrational bands at 1098, 1326, and 1358 cm^–1^.

Next, the boronic ester-polyesters were reacted under similar conditions ([−BPin] = 0.15 M, 2 vol % TFA, 10 h, room temperature). A solvent mixture (1:1 CH_2_Cl_2_:acetone) was required to maintain homogeneous polymer solutions throughout the reaction. The polyester samples were isolated by evaporation of the volatile species, under vacuum, followed by polymer precipitation in distilled water. The conversion to B(OH)_2_-polyester was very high (>95%), as evidenced by the near total disappearance of the pinacol ester methyl resonances in the ^1^H NMR spectra of the isolated polymers (Figures S40–S49). The polyester ^11^B NMR spectra showed changes to the chemical shifts from ∼30.8 ppm (boronic ester) to ∼28.3 ppm (boronic acid). The IR spectra showed the loss of characteristic −BPin vibrational bands,^71^ for example, P(BPin-PA/vCHO) showed the complete disappearance of resonances at 1098, 1326, and 1358 cm^–1^ assigned to the −BPin groups (Figure 5).

**Figure 6:**
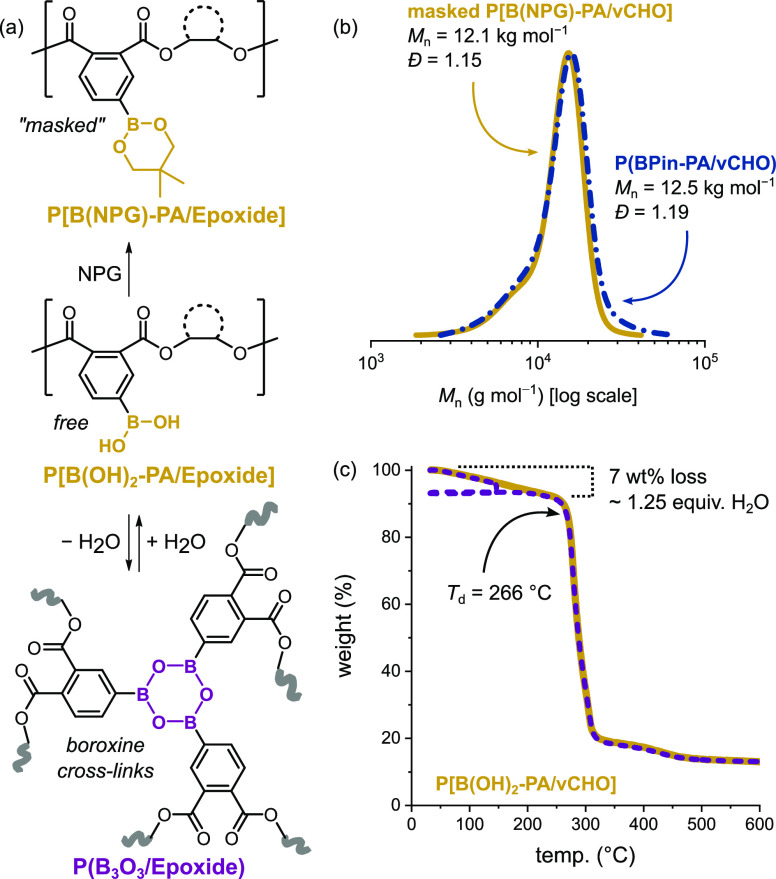
(a) Summary of reactions undertaken to demonstrate the dynamic covalent chemistry of boronic acid-polyesters. (b) GPC traces of P[B(OH)_2_-PA/vCHO] masked with NPG (solid yellow) and parent P(BPin-PA/vCHO) (dashed blue). (c) Thermogravimetric analyses (TGA) of P[B(OH)_2_-PA/vCHO]: experiments conducted with direct temperature increases (solid yellow), and with a 30 min isotherm at 150 °C prior to temperature increase (dashed purple) (ramp rate = 10 °C·min^–1^).

To establish whether any polyester backbone hydrolysis occurred during deprotection, polymers were analyzed by gel permeation chromatography (GPC). Curiously, the B(OH)_2_-polyesters show very broad molar mass distributions, despite having high solubility in THF (vide infra). The peak broadening may arise from backbone hydrolysis or could be due to boronic acid aggregation and/or interactions with the GPC columns. To investigate further, the B(OH)_2_-polyesters were reacted in situ with a 1,3-diol (neopentyl glycol, NPG), in THF, as the transesterification equilibrium is, of course, reversible (Figure 6a).^72,73^ This strategy proved very effective since the resulting B(NPG)-polyesters showed well-defined, narrow dispersity peaks in the GPC. It should be noted that when conducting the reactions in the GPC vials, a relatively large excess of NPG was required [10–20 equiv vs −B(OH)_2_ units] (Figures S50–S53). For example, reacting both P[B(OH)_2_-PA/vCHO] and P[B(OH)_2_-PA/PO] with NPG resulted in polymers showing nearly identical molar mass and dispersity to the starting BPin-polyesters (Figure 6b).

The ability to react the boronic acids with NPG was also effective for the analysis of the polymers using MALDI-TOF (Figures S54–S56). For example, reacting P[B(OH)_2_-PA/vCHO] with excess NPG, resulted in mass distributions with repeat units, obtained from the gradients of *m*/*z* vs *n*th repeat unit, of 383.93 g·mol^–1^ which were in excellent agreement with theoretical values of 384.17 g·mol^–1^ for P[B(NPG)-PA/vCHO]. Both GPC and MALDI-TOF data provide good evidence of the polyester stability during the boronic acid formation.

Boronic acid-polymers are also well-known to undergo thermally activated cross-linking reactions via boroxine formation, with elimination of water.^19,74^ This reactivity has been quite successful in producing self-healable and reprocessable elastomers, and responsive polymer networks.^75−78^ Samples of solid B(OH)_2_-polyester were heated under vacuum (50–60 °C) and became swollen gels in good solvents (acetone, THF, dioxane, and methanol), pointing toward a cross-linked structure. If dehydration led to the formation of heteroboroxines as nodes in dynamic covalent networks, the reverse reaction (i.e., addition of water to re-form the boronic acids) should disrupt the cross-links. Indeed, the addition of even trace amounts of distilled water (or D_2_O) to the swollen gels resulted in instantaneous dissolution and formation of transparent polymer solutions which were employed for NMR and GPC characterization (vide supra). Thermograms, obtained by TGA and DSC, further demonstrated that the B(OH)_2_-polyesters eliminated water on heating (Figures S57–S64). Specifically, the TGA traces showed weight losses from 50 °C until the onset of thermal degradation (*T*_d_ 266–314 °C) consistent with elimination of 0.9–1.2 molecules of H_2_O per monomer repeat unit (Figure 6c). DSC thermograms also showed wide and intense endothermic events with peak maxima from 100 to 107 °C, consistent with B(OH)_2_-polyester dehydration. The resulting cross-linked polymers (B_3_O_3_-polyester) did not show any further transitions up to 250 °C (i.e., no glass transition temperatures), consistent with very low chain mobility after cross-linking.

### Functional Block Polyesters and Self-Assembly

**Scheme 2:**
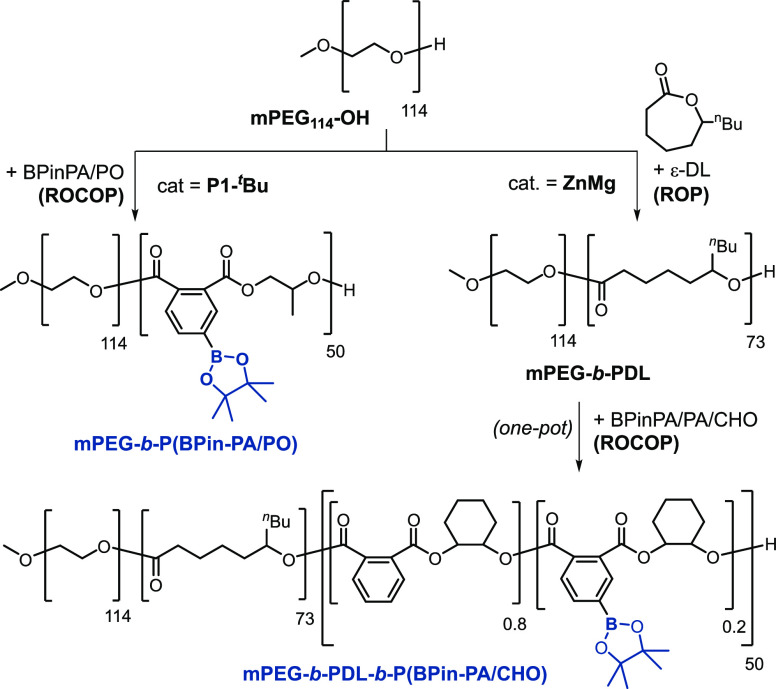
Synthesis of Boronic Ester-Block Polyesters

The controlled initiation and propagation of BPin-PA/epoxide ROCOP from alcohol groups prompted the investigation of hydroxyl-end-capped polymers as macroinitiators for AB and ABC block polymers [A = PEG, B = boronic ester-polyester, C = poly(ε-decalactone)] (Scheme 2). The goals in preparing these functional (multi)block polyesters were to (1) demonstrate the ability to prepare block polymers featuring selectively placed boron substituents; which might result in (2) polymer amphiphiles exploiting the hydrophobicity of boronic ester(acid)-containing blocks; and allowing for the production of (3) fluorescent self-assembled nanoparticles in aqueous media. This progressive build-up of architectural and functional complexity should exemplify how to selectively install useful groups for future applications in medicine, imaging, or sensing. Indeed, these fields greatly benefit from the development of boron-containing nanostructured materials.^79−83^

**Figure 7:**
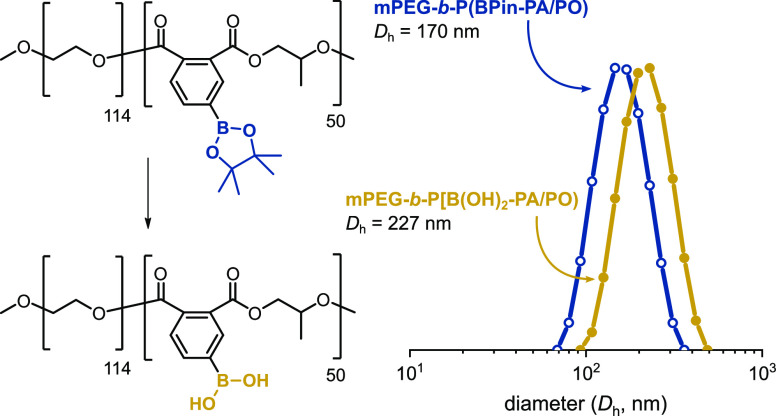
(Left) Structures of amphiphilic AB block polymers, mPEG-*b*-P(BPin-PA/PO), and mPEG-*b*-P[B(OH)_2_-PA/PO]. (Right) Particle size distribution, measured by DLS, of boronic ester- (blue) and boronic acid-block polymers (yellow) (THF/H_2_O 1:9, 0.25 mg·mL^–1^).

First, AB block polymer amphiphiles were prepared by initiating BPin-PA/PO ROCOP from hydrophilic methylpoly(ethylene glycol) (mPEG–OH) (*M*_n_ = 4.13 kDa, *Đ* = 1.12), with **P1-**^***t***^**Bu** (see the Suporting Information for details). The polymerization proceeded to complete anhydride conversion forming a copolymer featuring the P(BPin-PA/PO) block. Polymer analyses, by ^1^H NMR spectroscopy and GPC (*M*_n_ = 15.9 kDa, *Đ* = 1.10), corroborated the AB block polymer structure, i.e., mPEG-*b*-P(BPin-PA/PO) (Figures 7 and S65 and S66). Once again, the boronic ester groups were reacted by transesterification with excess MBA, to form boronic acid-containing block polymers, mPEG-*b*-P[B(OH)_2_-PA/PO]. The ^1^H NMR spectra of the polymers were consistent with boronic acid formation, while GPC traces (after reaction with NPG) showed similar molar mass values and narrow dispersity values (*M*_n_ = 12.8 kDa, *Đ* = 1.24) suggesting no backbone degradation (Figures S67 and S68).

The new AB block polymers contain a hydrophilic PEG and hydrophobic polyester block; thus, their self-assembly was investigated by addition of excess distilled water to block polymer solutions in THF (THF/H_2_O 1:9, 0.25 mg·mL^–1^) (Figure 7). Studies by dynamic light scattering (DLS) of the resulting spherical nanostructures corroborated their amphiphilic nature before and after deprotection. First, the PEG-*b*-P(BPin-PA/PO) self-assembled to form unimodal nanoparticles with an average hydrodynamic diameter, *D*_h_, of 170 nm and narrow dispersity. Interestingly, the boronic acid polymer, mPEG-*b*-P[B(OH)_2_-PA/PO] exhibited even larger size, with average diameters of 227 nm and narrow dispersity. These results suggest greater aggregation and increased hydrophobicity for the boronic acid (vs ester).

Next, the formation of ABC block polymers was attempted using the **ZnMg** catalyst for both anhydride/epoxide ROCOP and lactone ROP. The polymerization was initiated using mPEG_114_–OH and started with the polymerization (ROP) of ε-decalactone (ε-DL), followed, in one pot and without work-up, by BPin-PA/PA/CHO ROCOP. The reaction relies on a mechanistic “switch” in the catalysis from lactone ROP into epoxide/anhydride ROCOP, triggered by the addition of the anhydride. The switch occurs because the anhydride inserts faster into the propagating metal-alkoxide than the lactone. The resulting metal-carboxylate intermediate (after anhydride insertion) cannot react with the lactone but can undergo epoxide/anhydride ROCOP.^49^ Accordingly, the reaction of [**ZnMg**]/[mPEG_114_–OH]/[ε-DL] (1:4:315), in a mixture of epoxide (CHO) and toluene at 80 °C, resulted in near complete conversion of ε-DL (93%) in 20 min. An aliquot removed at this point confirmed the formation of an AB block polymer, i.e., mPEG-*b*-PDL with molar mass values in good agreement with theoretical values and narrow dispersity (*M*_n_ = 13.6 kDa, *D̵* = 1.22). At this point, a mixture of two anhydrides, BPin-PA/PA (1:4, 200 equiv), was added resulting in epoxide/anhydride ROCOP. The polymerization was maintained at 80 °C for a further 26 h, resulting in the complete consumption of both anhydrides, as evidenced by ^1^H NMR spectroscopy (Figure S69). The isolated ABC poly(ethylene glycol)-*b*-poly(ε-decalactone)-*b*-poly(pinacolboronic ester-phthalate-*alt*-cyclohexene oxide), mPEG-*b*-PDL-*b*-P(BPin-PA/CHO-*ran*-PA/CHO) showed an increase in its molar mass and maintained narrow dispersity, monomodal molar mass distributions (*M*_n_ = 20.4 kDa, *D̵* = 1.19). The ABC block polymer showed relative degrees of polymerization, DP_*n*_, as determined by NMR spectroscopy, of 114 (m-PEG), 73 (PDL), and 50 P(BPin-PA/PA/CHO) blocks, respectively. These values were consistent with the monomer stoichiometries and conversions vs catalyst and initiator (Figure S70). The block polymer structures feature ∼26 wt % hydrophilic mPEG block (expected to provide sufficient amphiphilic character) and ∼10 boronic ester (BPin) groups per chain (ensuring sufficient sites for BODIPY addition) separated by, on average, 4 PA/CHO repeat units.

**Figure 8:**
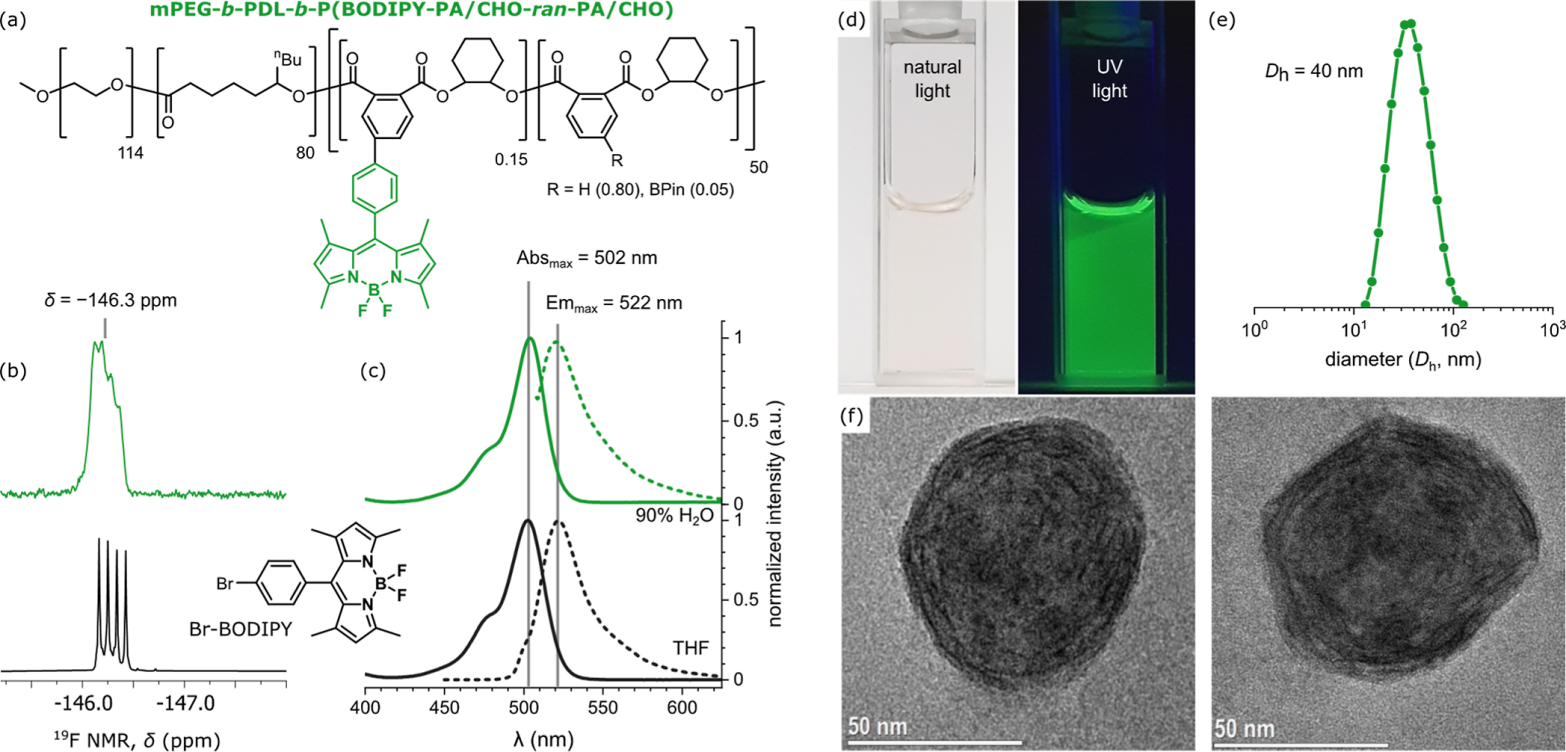
(a) Structure of ABC amphiphilic block polymer, mPEG-*b*-PDL-*b*-(BODIPY-PA/CHO-*ran*-PA/CHO); (b) ^19^F NMR spectra (CDCl_3_) of BODIPY-block polymer (top, green) and molecular BODIPY fluorophores (bottom, black); (c) UV–vis absorption (solid line) and emission (dotted line) spectra in solution for BODIPY-polymer (green lines, 90% H_2_O, 0.20 mg·mL^–1^) and Br-BODIPY (black lines, THF); (d) photographs under natural light and UV-irradiation illustrating the strong fluorescence of the self-assembled BODIPY-block polymer nanoparticles in 90% water; (e) particle size and distribution, measured by DLS of the BODIPY-block polymer nanoparticles (90% H_2_O, 0.20 mg·mL^–1^); and (f) transmission electron micrographs of BODIPY-block polymer nanoparticles.

Next, the ABC triblock polymer was modified to install fluorescent moieties by the Suzuki–Miyaura cross-coupling reaction of the pendent −BPin moieties with a bromo-substituted BODIPY. Accordingly, mPEG-*b*-PDL-*b*-P(BPin-PA/CHO-*ran*-PA/CHO) was reacted under typical cross-coupling conditions (0.1 equiv Pd(OAc)_2_, 0.2 equiv SPHOS, 3.0 equiv K_2_CO_3_, 100 °C, toluene/water (10:1); see the Supporting Information for reaction details). After 24 h, ∼70% of the boronic ester groups were converted to the corresponding BODIPY substituents according to analysis by ^1^H NMR spectroscopy (Figures 8a and S74). These high conversion levels provide a final average number of BODIPY units per chain of ∼7.5. The polymer was isolated by precipitation in Et_2_O and filtration through silica. In comparison, a boronic ester-polyester, P(BPin-PA/CHO), reached >99% boronic ester conversion under identical conditions (Figures S71–S73). The slightly reduced boronic ester conversion in the ABC block polymer may arise due to the dilute conditions and highly viscous reaction media. The successful installation of BODIPY units onto the ABC polymer was supported by several different characterization methods. The ^1^H NMR spectrum of the isolated functionalized block polymer showed characteristic signals corresponding to the dipyrromethene group (5.99 and 2.57 ppm, Figure S74). The ^19^F and ^11^B NMR spectra also showed broadened signals in the expected regions for the fluorophore (−146.3 ppm and 0.7 ppm, respectively) (Figures 8b, and S74 and S75). The polymer nanoparticles, dispersed in THF/H_2_O (1:9, 0.20 mg·mL^–1^), gave strong UV–vis absorptions and green light emission (502 and 522 nm, respectively) which almost perfectly matched those of the molecular analogue (Figure 8c). Indeed, the aqueous solution had a faint yellow color under ambient light, but exhibited a strong green fluorescence under UV-irradiation, typical of BODIPY dyes (Figure 8d). The particle size distributions, measured by DLS in 90% H_2_O (0.20 mg·mL^–1^), revealed nanoparticles with uniform diameters of 40 nm which were only slightly smaller than the unfunctionalized nanoparticles [*D*_h_ = 53 nm for mPEG-*b*-PDL-*b*-P(BPin-PA/CHO-*ran*-PA/CHO)] (Figure 8e).

Transmission electron microscopy (TEM) further confirmed the formation of uniaxial (spherical) polymer nanoparticles (Figure 8f). The spherical self-assembled structures showed diameters of ∼50 nm, in close agreement with DLS measurements. Interestingly, the TEM images revealed distinct polymer domains inside the nanoparticles (dark and light regions), indicating some block phase separation between PDL and anhydride/epoxide polymer blocks within the core. Similar phase separation of PDL and (other) anhydride/epoxide polyester blocks, although yet to be observed by TEM, has been previously detected by small angle X-ray scattering (SAXS) and exploited in the formation of thermoplastic elastomers.^53^ These analyses indicate the formation of spherical micelles composed of a PEG corona (not visible in TEM) and a phase-segregated polyester, PDL-*b*-(BODIPY-PA/CHO-*ran*-PA/CHO), core. These combined findings highlight the future potential to develop new functional materials by exploiting the amphiphilic character of multiblock polymers and cross-coupling processes to install other chemical functionalities.

### Polymer Water Solubility and Hydrolysis

Water-soluble, yet degradable, polyesters would be useful for liquid formulations, drug delivery, and tissue scaffolds. So far, there are only a few water soluble polyesters and hydrophilicity is typically achieved by post-polymerization installation of alcohols, carboxylic acids, PEG chains, or electrolytes.^56,84−87^ Alternatively, pH-dependent ionization of boronic acids has been used to induce increased hydrophilicity and water solubility into polystyrenes.^23,88^ We reasoned that similar transformations should also render these B(OH)_2_-polyesters water-soluble. One aspect to consider is that, under such conditions, there may also be an opportunity for base-catalyzed hydrolytic degradation of the polyester backbone. Thus, the formation of borate salts may offers a strategy to trigger, accelerate, and monitor in situ polyester degradation.

**Figure 9:**
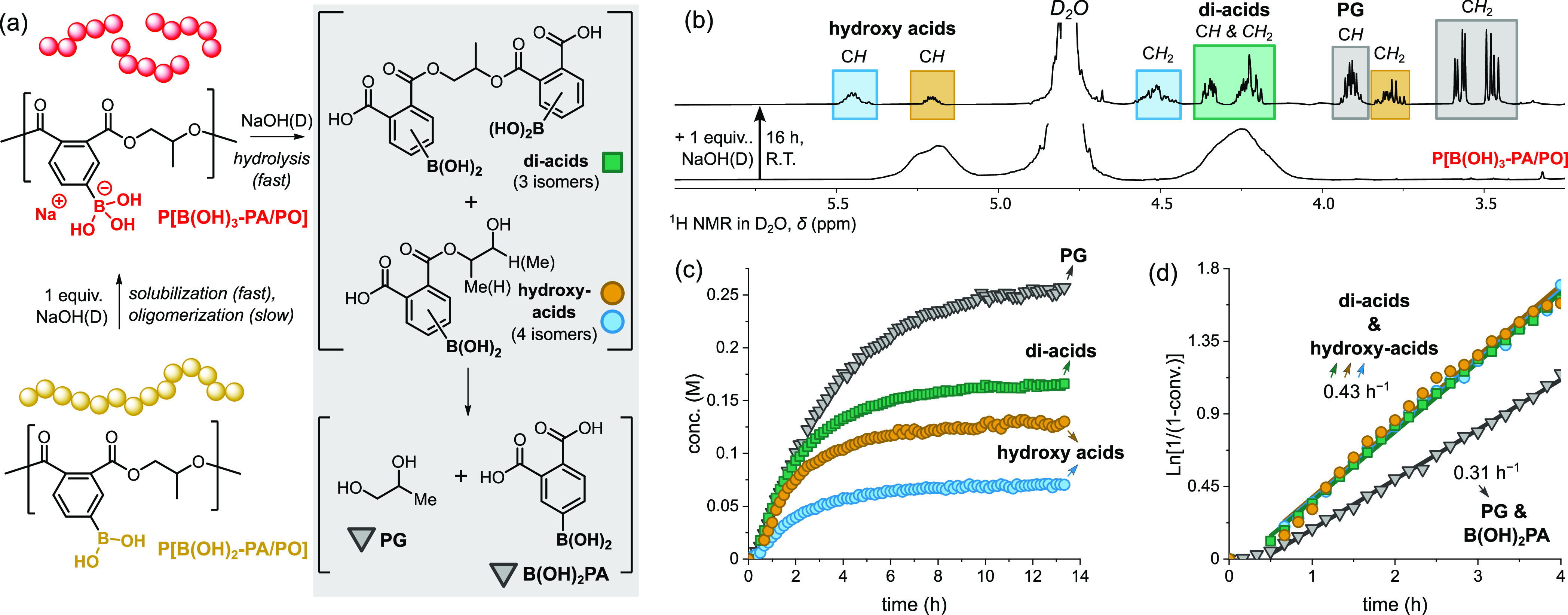
(a) Experiments to form borates from boronic acid polyesters, and the chemical structures of degradation products (in neutral form). (b) Portion of the ^1^H MMR spectra (D_2_O, 25 °C) of water-soluble P[B(OH)_3_-PA/PO] (bottom), and 16 h after the addition of 2 equiv of NaOD (top). (c) Monitoring the polyester hydrolytic degradation using P[B(OH)_3_-PA/PO] with degradation product concentration vs time plots. (d) Semi-logarithmic plots of degradation product concentration vs time, with associated rate coefficients (*k*_obs_ as the gradient to linear fits).

As proof of potential, suspensions of neutral B(OH)_2_-polyester, in D_2_O, were treated with NaOD (1 equiv per boronic acid). The solutions were vortexed at room temperature, after which they became transparent and were immediately analyzed by ^1^H and ^11^B NMR spectroscopy. The spectra showed broad resonances consistent with the formation of soluble B(OH)_3_–polyesters specifically, the large shifts (Δ ∼ 24 ppm) in the ^11^B NMR spectra to 1.9–2.3 ppm signal formation of tetracoordinated borate anions (Figures S79–S86). To assess polymer stability under ambient conditions, the ^1^H NMR spectrum of P[B(OH)_3_-PA/PO] was monitored over time. Interestingly, the broad polymer resonances remained almost completely unchanged for up to a week, indicating that the polymer backbone was mostly unreacted (Figure S87). After around 1 month at room temperature, new sharper signals, in addition to the polymer resonances, were observed in the ^1^H NMR spectrum, indicating the on-set of polyester degradation (Figure 9a, vide infra). To gain further insights, an identical fresh sample of P[B(OH)_3_-PA/PO] was immediately neutralized with benzoic acid and analyzed by GPC in the presence of excess NPG. A low MW shoulder and slight broadening to the molar mass distribution suggested that slight backbone fragmentation occurred upon solubilization (Figure S88). The solution was maintained at room temperature for 4 days, after which the polymer molar mass had significantly decreased and the dispersity broadened (*M*_n_ = 3.78 kg/mol and *Đ* = 1.41). These findings indicated that slow oligomerization occurred, likely through free hydroxide anions in equilibrium with the borate anions, which slowly catalyze polyester hydrolysis.

Using more forcing conditions, degradation was accelerated, allowing insights into the rates of hydrolysis. Starting from a fresh solution of P[B(OH)_3_-PA/PO], in D_2_O at 25 °C, 2 equiv of NaOD (per boronic acid) were added and the degradation reaction was monitored by ^1^H NMR spectroscopy (Figure S89). After 2 h, the polymer resonances disappeared and the formation of propylene glycol (PG), together with other hydrolysis products, reached equilibrium over 12 h (Figures 9b,c). Analysis of the final solution by high-performance liquid chromatography mass spectrometry (HPLC–MS) revealed a mixture of phthalate esters (in particular, three di-acid and four hydroxy-acid regio-isomers), and the phthalic acid, B(OH)_2_-PA, as the other major decomposition products (Figures S92–S99 and Table S4). Importantly, the boronic acid moieties were present in all the phthalate degradation products. With the degradation products confirmed by HPLC-mass spectrometry, the new signals previously noted in the ^1^H NMR spectrum were assigned using multinuclear ^1^H–^1^H COSY and ^1^H–^13^C HMBC NMR experiments (Figures S90–S91). Using dimethyl sulfone as an internal standard, the total equilibrium concentration of the different di-acids (overlapping signals at 4.34 and 4.22 ppm) and hydroxy acids (two sets of overlapping signals at 5.45, 5.20, 4.51, and 3.78 ppm) were calculated. The two concentrations were very similar: [di-acids]_total_ = 0.17 M vs [hydroxy-acids]_total_ = 0.20 M. In contrast, the equilibrium concentrations of diol (PG) (3.57, and 3.47 ppm) was slightly higher, [PG]_total_ = 0.25 M. Kinetic plots revealed that the rates of formation of all the di- and hydroxy-acids were equivalent (*k*_1_ = 0.43 h^–1^) and the formation of PG was somewhat slower (*k*_2_ = 0.31 h^–1^) (Figure 9d). It is worth noting that other diol species (i.e., HO-PO-PA-PO-OH) were not detected by ^1^H NMR spectroscopy or HPLC–MS.

These experiments allow formulation of a polymer degradation (hydrolysis) mechanism (Scheme S1). When the polyester-B(OH)_2_ was exposed to one equivalent of NaOH(D), there was rapid and quantitative ionization to give the borate ions (p*K*_a_ of B(OH)_2_-DMP ∼ 7.0, Figure S100). Nonetheless, there were sufficient hydroxide anions, in equilibrium with the borate anions, to slowly catalyze random chain scission processes (i.e., ester hydrolyses) at room temperature. The polyester degradation resulted in oligomerization over the first few days. Then, fragmentation into various small-molecules, mostly comprising dimeric esters, occurred. These dimers underwent further hydrolysis to form di-acids or hydroxy-acids depending on which ester group was hydrolyzed. These species reacted further with water, more slowly, to form the diol (PG) and the hydrolyzed phthalic acid [B(OH)_3_-PA] monomers.

## Conclusions

A series of generally applicable syntheses and reactions of boronic ester-, acid-, and borate-polyesters were described, giving rise to amphiphilic and functional (multi)block polyesters. The methods allow for: (1) direct polymerization, and copolymerization, of the boronic ester-monomer at rates and selectivity equivalent to unfunctionalized monomers; (2) modulation of the polymer structures and properties; and (3) transformations of boronic ester-substituents into boronic acids-, borates-, and fluorescent groups. The boronic ester-polyesters (polyester-BPin) were synthesized using ROCOP of pinacol boronic ester-PA with four different epoxides, catalyzed by either organometallic complexes or a organophosphazene “superbase” in the presence of alcohols. All polymerizations were fast, selective, and well controlled. The new boronic ester-polyesters showed amorphous structures with high glass transition temperatures (81–224 °C) and good thermal stability (285–322 °C), important features for future applications as plastics or thermoplastic elastomers. The pinacol boronic ester substituents were efficiently deprotected into boronic acid polyesters [B(OH)_2_-polyester] without compromising the integrity of the polyester backbone. These boronic acid-polyesters spontaneously underwent reversible cross-linking by dehydration to boroxines. The syntheses were also applied in more complex AB and ABC block polymer amphiphile structures and used to install fluorescent markers via cross-coupling processes. These materials self-assembled in aqueous solution to spherical nanostructures. Finally, the boronic acid substituents were transformed into borates which triggered polyester hydrolysis and ultimately complete degradation.

This paper demonstrates a synthetic platform to obtain a range of boron-substituted polyesters. The use of easily accessible BPin-PA monomer and compatibility with existing polymerization methods can easily be extended to make other, yet more complex, structures including through copolymerizations with other heterocycles (oxiranes, thiiranes, and aziridines), heterocumulenes (carbon dioxide and carbon disulfide), and cyclic monomers (carbonates, esters, and pyrolidones). Thanks to the orthogonality of the −BPin substituents and the plethora of coupling reagents available, this strategy should allow decoration of other semi-aromatic polyester backbones. After deprotection, the water solubility and degradation of the resulting -B(OH)_2_ containing polymers could be triggered on demand. These new materials warrant future investigations in biology, medicine, sensing, and responsive cross-linked (elastomeric) plastics.

## Supporting Information Available

Full details on monomer and polymer synthesis and characterization; NMR, GPC, and thermal data; and reaction methods (PDF)
