# Supplementary material for: Ring Opening Copolymerization of Boron-Containing Anhydride with Epoxides as a Controlled Platform to Functional Polyesters
Source: J Am Chem Soc. 2023 Jun 13;145(25):13888–900. doi: 10.1021/jacs.3c03261 (PMC10311538; doi:10.1021/jacs.3c03261)
Supplement: Supplementary file 1 — ja3c03261_si_001.pdf [file ja3c03261_si_001.pdf]

Supporting Information

Ring Opening Copolymerization of Boron-Containing Anhydride with Epoxides as a Controlled Platform to Functional Polyesters

Fernando Vidal,* Sevven Smith, Charlotte K. Williams*

Chemistry Research Laboratory, University of Oxford, 12 Mansfield Road, Oxford, UK, OX1 3TA

Table of Contents

1. Materials, Instruments, and General Methods

2. Synthesis and Characterization of Boron Compounds

2.1. Synthesis of phthalic anhydride pinacolboronate ester, (BPin-PA)
2.2. Synthesis of dimethyl phthalate pinacolboronate ester, (BPin-DMP)
2.3. Synthesis of dimethyl phthalate boronic acid, [$B(OH)_2$-DMP]

3. ROCOP of BPin-PA with Epoxides

3.1. Typical BPin-PA/epoxide ROCOP.
3.2. Kinetics of BPin-PA/epoxide ROCOP and PA/epoxide ROCOP using [ZnMg].
3.3. NMR spectra of BPin-PA polyesters
3.4. Gel permeation chromatography (GPC) of BPin-PA polyesters
3.5. MALDI-TOF of P(BPin-PA/vCHO)
3.6. Summary of thermal properties of semi-aromatic polyesters
3.7. Differential scanning calorimetry (DSC) of BPin-PA polyesters
3.5. Thermogravimetric analysis (TGA) of BPin-PA polyesters

4. Deprotection to Polymeric Boronic Acids

4.1. Investigation of boronic ester transesterification using small-molecule model
4.2. General method for the deprotection of $B(OH)_2$ polyesters
4.3. GPC analysis of $B(OH)_2$ polyesters
4.4. MALDI-TOF of P[B(NPG)-PA/vCHO]
4.5. Differential scanning calorimetry (DSC) of $B(OH)_2$ polyesters
4.6. Thermogravimetric analysis (TGA) of $B(OH)_2$ polyesters

5. Synthesis and Characterization of Boron-containing Block-copolymers

5.1. Synthesis of poly(ethylene glycol)-*b*-poly(pinacolboronate phthalate-alt-propylene), *m*PEG-*b*-P(BPin-PA/PO)
5.2. Synthesis of poly(ethylene glycol)-*b*-poly(boronic acid phthalate-alt-propylene), *m*PEG-*b*-P[$B(OH)_2$-PA/PO].
5.3. Synthesis of poly(ethylene glycol)-*b*-poly(ε-decalactone)-*b*-poly(phthalate/pinacolboronate phthalate-alt-cyclohexene), mPEG-*b*-PDL-*b*-P(BPin-PA/CHO)

6. Suzuki-Miyaura Cross-Coupling post-polymerization modification

6.1. Synthesis of poly(phthalate/BODIPY-alt-cyclohexene), P(BODIPY-PA/CHO).
6.2 Synthesis of poly(ethylene glycol)-*b*-poly(ε-decalactone)-*b*-poly(phthalate/BODIPY phthalate-alt-cyclohexene), *m*PEG-*b*-PDL-*b*-P(BODIPY-PA/CHO-*ran*-PA/CHO)

7. Aqueous Solution Studies of Polymeric Boronic Acids

7.1. NMR scale investigations in water

7.2. Hydrolytic stability in basic aqueous media
7.3. Monitoring of polymer degradation by NMR spectroscopy
7.4. Liquid Chromatography-Mass Spectrometry of degradation products
7.5. Proposed hydrolysis mechanism

8. Potentiometric Titration of dimethyl phthalate boronic acid, [B(OH)$_2$-DMP]

9. References

1. Materials, Instruments, and General Methods

Materials. All oxygen- and moisture-sensitive manipulations were carried out under an inert atmosphere using either standard Schlenk techniques on a dual-manifold Schlenk line or in a N_2-filled glovebox. Toluene, hexanes, and tetrahydrofuran, where collected under an inert atmosphere from a Solvent Purification System, degassed via three freeze/pump/thaw cycles, and stored in a N_2-filled glovebox over dry molecular sieves.

Bis(pinacolato)diboron, 4-bromophthalic anhydride, methylboronic acid, dimethyl 4-hydroxyphthalate and dimethyl 4-bromophthalate were purchased from Fluorochem (Ltd.); cyclohexene oxide (CHO) was purchased from Acros Organics; dichloro[1,1'-bis(diphenylphosphino)ferrocene]palladium(II), and 1,4-benzenedimethanol (BDM) were purchased from Alfa Aesar; palladium(II) acetate, SPhos, potassium acetate, anhydrous 1,4-dioxane, trifluoroacetic acid, 2,2-dimethyl-1,3-propanediol (neopentyl glycol, NPG), tert-butylimino-tris(dimethylamino)phosphorene (P_1-^{t}Bu), epsilon-decalactone (εDL), 4-vinyl-1-cyclohexene 1,2-epoxide (vCHO), allyl glycidyl ether (AGE), and (+/-)-propylene oxide (PO) were purchased from Sigma-Aldrich. Literature procedures were followed to prepare the following compounds: [ZnMg] catalyst,[1] [AlK] catalyst,[2] 10-(4-bromophenyl)-5,5-difluoro-1,3,7,9-tetramethyl-5H-4l4,5l4-dipyrrolo[1,2-c:2',1'-f][1,3,2]diazaborinine (Br-BODIPY).[3]

Epoxides were dried from CaH_2 at room temperature over 48 h, vacuum distilled, and stored under inert atmosphere inside the glovebox. CHO was further treated with NaH over 24 h and vacuum distilled (50 °C, 200 mbar). BDM was recrystallized from dry toluene and stored under inert atmosphere inside the glovebox. All other commercial reagents were used as received without further purification.

NMR spectroscopy. NMR data were acquired at 25 °C on a Bruker Avance III HD nanobay 400 MHz spectrometer (400.2 MHz ^{1}H, 128.4 MHz ^{11}B, 100.6 ^{13}C, 376.5 ^{19}F, 162.0 ^{31}P), or on a Bruker Avance III HD 500 MHz spectrometer (499.9 MHz ^{1}H, 160.4 MHz ^{11}B, 125.7 MHz ^{13}C, 470.4 MHz ^{19}F and 202.4 MHz ^{31}P). ^{1}H and ^{13}C{^{1}H} spectra were referenced internally to solvent signals: chemical shifts are reported as parts per million relative to $SiMe_4$. ^{11}B{^{1}H} spectra were recorded with background suppression. Deuterated solvents (Cambridge Isotope Laboratories Inc.) were used as received, except for application with moisture sensitive materials in which case they were dried over CaH_2, vacuum distilled, and stored over molecular sieves prior to use.

Other spectroscopy. UV-visible absorption data were acquired on a Shimadzu UV-1800 spectrometer. The fluorescence data were acquired using a Perkin-Elmer LS-55 Luminescence Spectrophotometer. Fourier-Transform Infrared Spectroscopy (FT-IR) was measured on a Shimadzu IRSpirit.

Differential Scanning Calorimetry (DSC). Polymer samples were analysed on a DSC25 (TA Instruments) under a N_2 flow (50 mL min^{-1}). Samples were heated (10 °C min^{-1}) and equilibrated to 250 °C to remove their thermal history, then cooled to −80 °C (10 °C min^{-1}) before heating a second time to 250 or 300 °C at a rate of 10 °C min^{-1}. Glass transition temperatures (T_g) were determined from the midpoint of the transition in the second heating curve.

Thermal Gravimetric Analysis (TGA). Polymer samples were analysed on a TGA/DSC 1 (Mettler-Toledo Ltd), or a TGA5500 (TA Instruments). Powder polymer samples were heated from 30 to 700 °C at a rate of 10 °C min^{-1} under N_2 flow (50 mL min^{-1}).

MALDI-TOF Mass Spectrometry. MALDI-TOF analyses were carried out on a Bruker Autoflex Speed MALDI-TOF. Spectra were acquired in positive-reflectron mode. Spotting method A, for pinacolboronate polyesters, P(BPin/epox.): sample solutions of polymer (10 mg mL^{-1} in THF), dithranol (10 mg mL^{-1} in THF), and NaI (10 mg mL^{-1} in MeOH) were pre-mixed in 1:4:1 ratio and subsequently spotted onto a metal plate and allowed to fully evaporate before analysis. Spotting method B, for free boronic acid polyesters masked with neopentyl glycol, P[B(OH)$_2$/epox.]: sample solutions of boronic acid polymer (10 mg mL^{-1} in THF with 1% vol. H_2O), dithranol (10 mg mL^{-1} in THF), neopentyl glycol (10 mg mL^{-1} in MeOH), and NaI (10 mg mL^{-1} in MeOH) were pre-mixed in 1:4:1:1 ratio and subsequently spotted onto a metal plate and allowed to fully evaporate before analysis.

Dynamic Light Scattering. Aqueous polymer samples (90:10 H_2O/THF) were analysed using a Zetasizer Pro Blue system (Malvern Instruments). The scattering angle was fixed at 173°. Data processing was carried out using cumulant analysis of the experimental correlation function and the Stokes−Einstein equation was used to calculate the hydrodynamic radii. All solutions were analysed using quartz cuvettes.

Gel Permeation Chromatography (GPC). Polymer samples (2-10 mg) dissolved in HPLC grade THF (1.2 mL) were syringe filtered through 25 μm PTFE filters. Next, they were injected into Shimadzu LC-20AD SEC instrument with two PSS SDV 5 μm linear M columns heated to 30 °C. HPLC grade THF was used as the eluent at a flow rate 1.0 mL min^{-1}. RI and UV detectors were calibrated using a series of narrow molecular weight polystyrene standards. Shimadzu SEC post run program was used to analyse the data. For experiments with free boronic acid polyesters, different amounts of neopentyl glycol (NPG) were added as describe in the main-text.

Elemental Analysis (EA). Elemental analyses were performed by Elemental Microanalysis Ltd, Hameldown Road, Okehampton Business Park, Exeter Road, Okehampton, Devon, EX20 1UB, UK.

Liquid Chromatography Mass Spectrometry (LC-MS). Degraded polymers were analysed using a Waters LCT Premier bench-top orthogonal acceleration time-of-flight LC-MS system, connected to a CTC Analytics HTS PAL Sample Manager, Acquity PDA Detector, Acquity Column Heater/Cooler and Acquity Binary Solvent Manager.

Transmission Electron Microscopy (TEM). Amorphous carbon-coated copper TEM grids (Agar Scentific) were plasma glow discharged for 15 s to generate a hydrophilic surface. Individual samples (2-4 μL) were placed onto the freshly discharged grids for 5 mins, before being blotted with a filter paper to remove excess solutions. The grids were dried in fume hood overnight prior to characterization. TEM images were obtained by Tianyi Chen on JEOL 3000F scanning transmission electron microscope, equipped with an Oxford X-Max 80 SDD EDX detector, at an operating voltage of 200 kV UV-vis and Fluorescence Spectroscopy.

2. Synthesis and Characterization of Boron Compounds

2.1. Synthesis of 5-(4,4,5,5-tetramethyl-1,3,2-dioxaborolan-2-yl)isobenzofuran-1,3-dione, (BPin-PA)

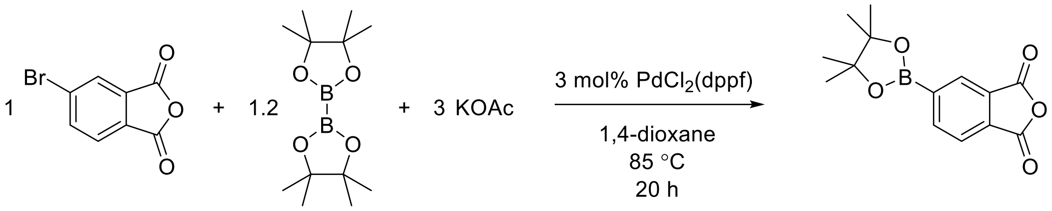

An oven-dried 500 mL round bottom Schlenk flask was loaded with a large magnetic stir bar and finely ground potassium acetate (13.0 g, 0.13 mol), capped and gently heated under high vacuum to remove any traces of moisture. After cooling to room temperature, 5-bromoisobenzofuran-1,3-dione (10.0 g, 0.044 mol), bis(pinacolato)diboron (13.5 g, 0.053 mol), and [1,1′-bis(diphenylphosphino)ferrocene]dichloropalladium(II), dichloromethane complex, (1.08 g, 1.32 mmol) were loaded, and the flask then placed under an inert atmosphere of N_2. Then, dry dioxane (250 mL) was added via cannula, the rubber septum was replaced with a Teflon-lined screw cap and the flask sealed under N_2. The reaction mixture was degassed, via three freeze/pump/thaw cycles, and then heated to 85 °C, with vigorous stirring for 24-48 hours, during which time a dark purple precipitate formed. Conversion was monitored by analysis of ^{1}H NMR spectra of reaction aliquots. Once the mixture had reached full conversion of the anhydride to the pinacolborate product, the slurry was cooled to room temperature, the solids filtered through a fine porosity frit, and the residue was thoroughly washed with EtOAc (3 × 100 mL). The resulting filtered solution was washed with brine (3 × 150 mL), the organic phase was dried (Na_2SO_4) and the solvents evaporated in a rotary evaporator to yield a dark brown oil. This residue was dispersed in silica gel and purified by flash column chromatography with hexanes/EtOAc (3:2 v:v) as eluting phase. The product was dissolved in hot toluene/hexane (1:9 v:v) solution (100 mL), hot-filtered to remove all insoluble impurities, and crystallized to remove excess bis(pinacolato)diboron. The resulting off-yellow solid was dried under vacuum and further purified by sublimation (117 °C, 0.005 mBar) to give a white crystalline powder consisting of the analytically pure product. Yield = 7.2 g (60%). Elemental Analysis for $C_{14}H_{15}BO_5$ (%): expected C, 61.35; H, 5.52; N, 0.00; found C, 61.37; H, 5.53; N, <0.05.

^{1}H NMR of **BPin-PA** ($CDCl_3$, 25 °C, 400.2 MHz): δ 8.44 (s, 1H, Ar-*H*); 8.30 (dd, J_1 = 0.8 Hz, J_2 = 7.6 Hz, 1H, Ar-*H*); 8.30 (dd, J_1 = 0.8 Hz, J_2 = 7.6 Hz, 1H, Ar-*H*); 7.99 (dd, J_1 = 0.8 Hz, J_2 = 7.4 Hz, 1H, Ar-*H*); 1.38 (s, 12H, BPin). ^{13}C{^{1}H} NMR of **BPin-PA** ($CDCl_3$, 25 °C, 100.6 MHz): δ 163.1, and 162.9 (*C*=O), 142.2, 133.3, 132.0, and 130.5 (Ar-*C*), 124.8 (Ar-*C*), 85.2 (O–*C*), 25.0 (–Me). ^{11}B{^{1}H} NMR of **BPin-PA** ($CDCl_3$, 25 °C, 128.4 MHz): δ 30.1.

FT-IR of **BPin-PA** (25 °C, cm^{-1}): 2975, 1850, 1771, 1617, 1597, 1491, 1425, 1392, 1381, 1372, 1359, 1349, 1314, 1268, 1244, 1214, 1171, 1144, 1108, 1062, 965, 926, 912, 879, 851, 819, 789, 740, 703, 695, 686, 666, 652, 639, 578.

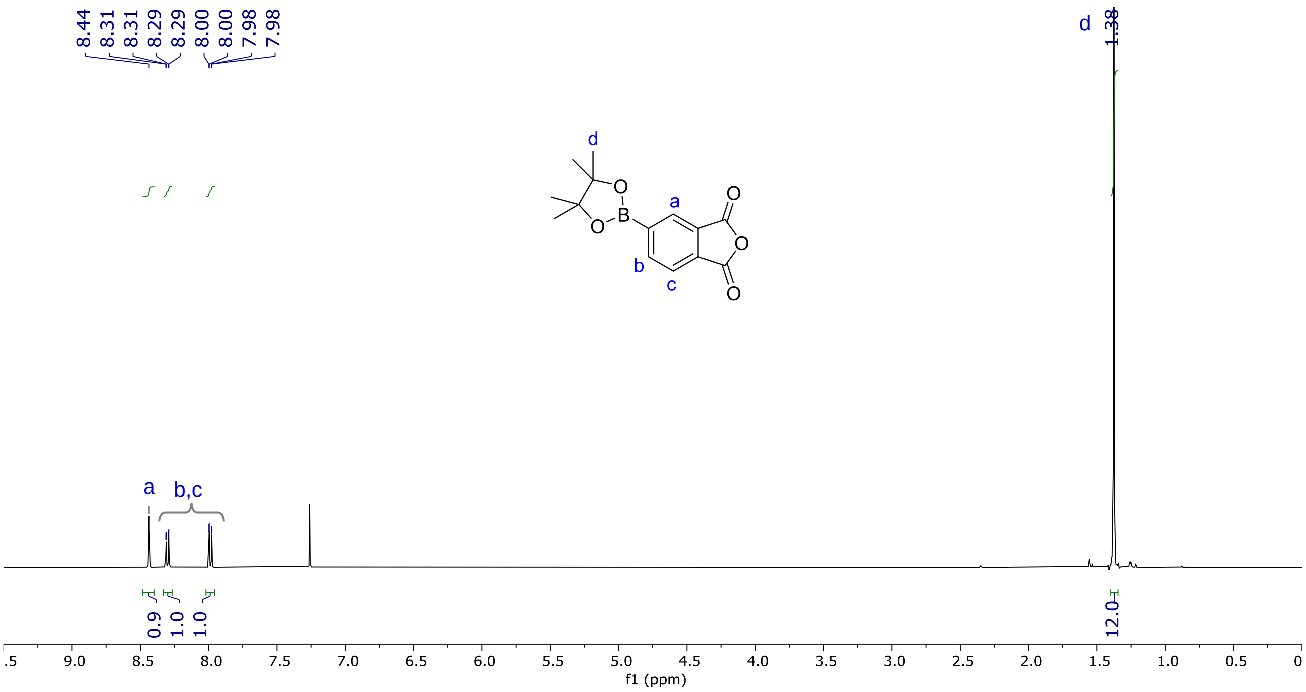

Figure S1. ^{1}H NMR ($CDCl_3$, 25 °C, 400.2 MHz) spectrum of **BPin-PA**.

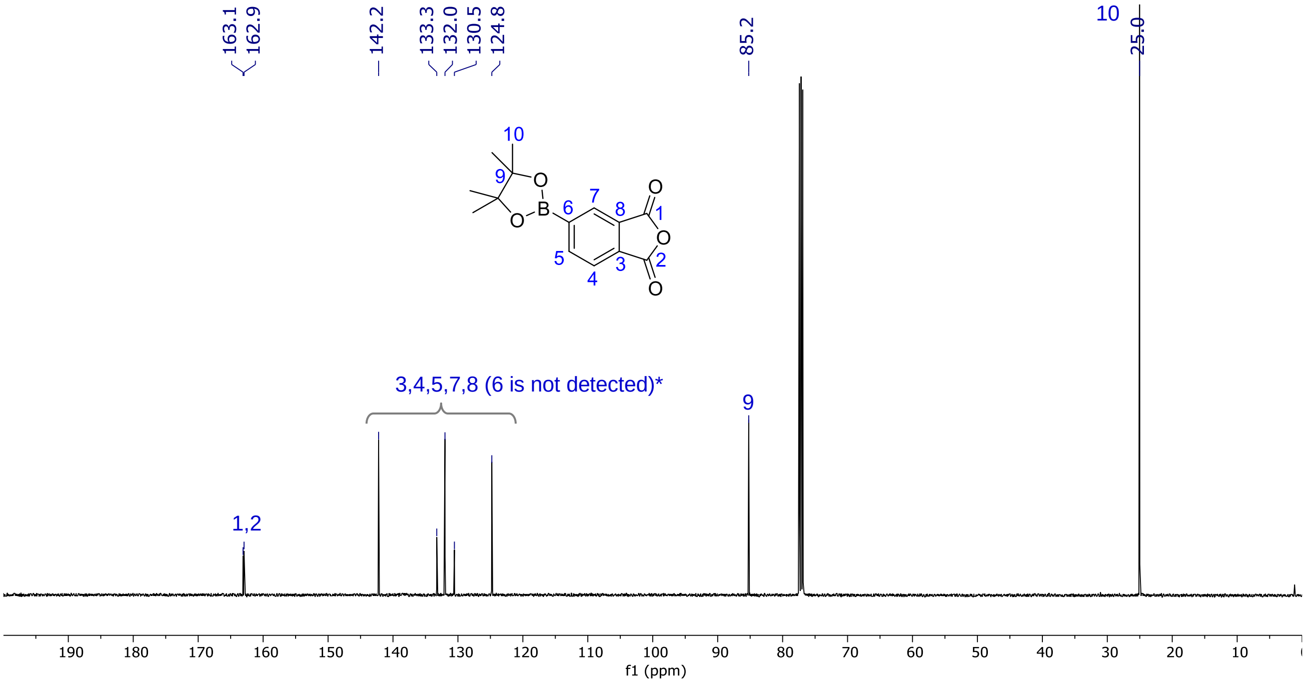

Figure S2. ^{13}C NMR ($CDCl_3$, 25 °C, 100.6 MHz) spectrum of **BPin-PA**. (* due to B–C coupling)

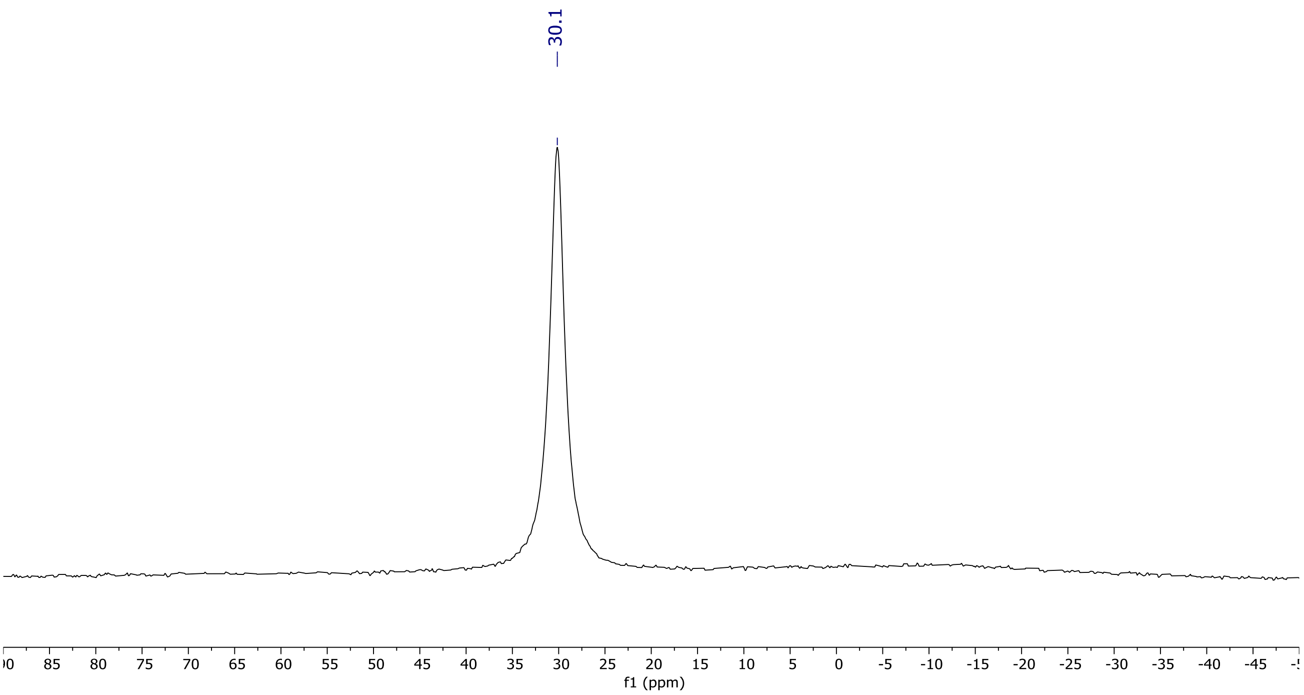

Figure S3. $^{11}B\{^{1}H\}$ NMR ($CDCl_3$, 25 °C, 128.4 MHz) spectrum of **BPin-PA**.

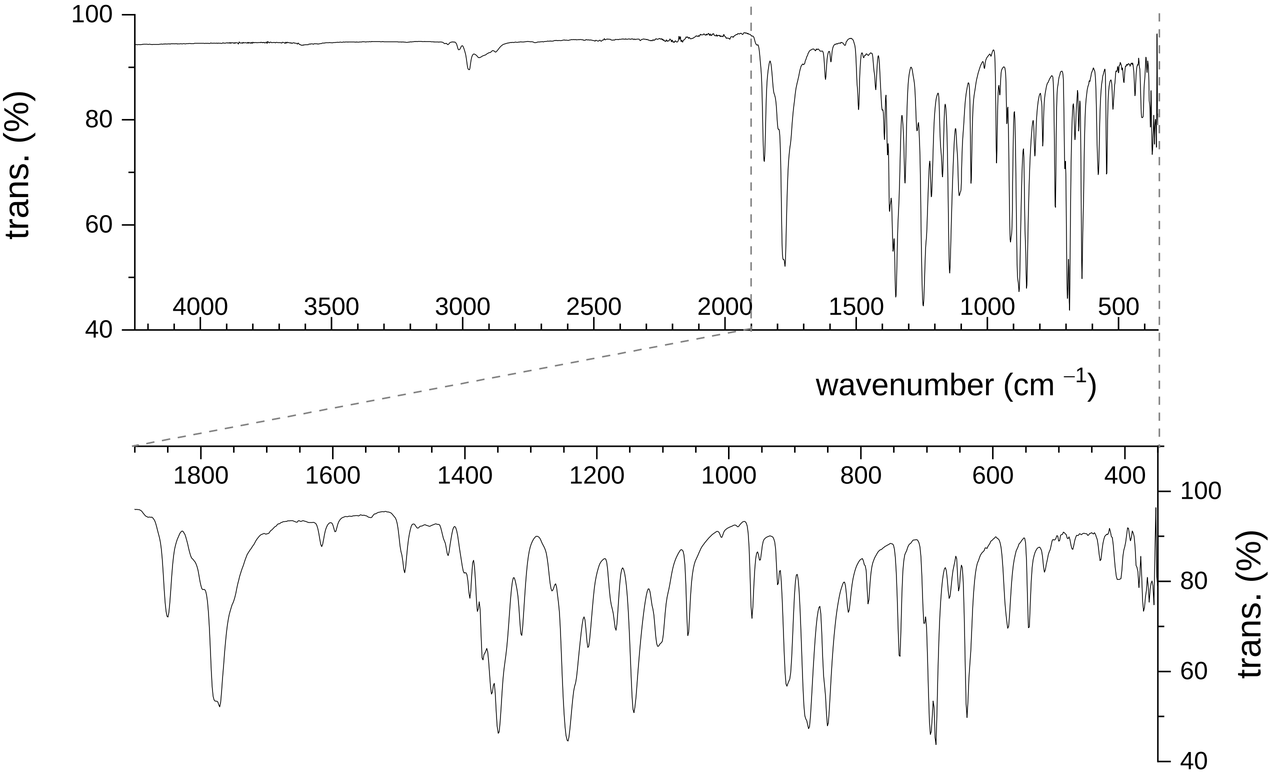

Figure S4. FT-IR spectrum at 25 °C of **BPin-PA** (top) and zoom in the 1900–350 cm^{-1} region (bottom).

2.2. Synthesis of dimethyl phthalate pinacolboronate ester, (BPin-DMP)

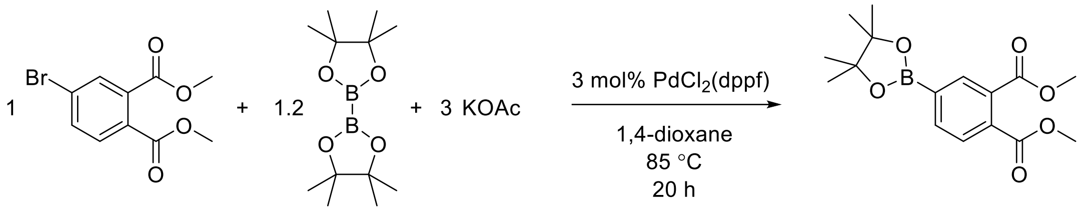

An oven-dried 250 mL round bottom Schlenk flask was loaded with a large magnetic stir bar and finely ground potassium acetate (5.42 g, 55.2 mmol), capped and gently heated, under high vacuum, to remove any traces of moisture. After cooling to room temperature, dimethyl 4-bromophthalate (5.00 g, 18.4 mmol), bis(pinacolato)diboron (5.61 g, 22.1 mmol), and [1,1′-bis(diphenylphosphino)ferrocene]dichloropalladium(II), dichloromethane complex, (0.45 g, 0.55 mmol) were loaded, and the flask then placed under an inert atmosphere of N_2. Then, dry dioxane (80 mL) was added by cannula, the rubber septum was replaced with a Teflon-lined screw cap and the flask sealed under N_2. The reaction mixture was degassed, via three freeze/pump/thaw cycles, and then heated to 85 °C, with vigorous stirring for 20 hours, during which time a dark purple precipitate formed. Analysis of a reaction aliquot by ^{1}H NMR spectroscopy indicated full conversion to the pinacolborate product. Subsequently, the slurry was cooled to room temperature, the solids filtered through a fine porosity frit, and the residue was thoroughly washed with EtOAc (3 × 100 mL). The resulting filtered solution was washed with brine (3 × 150 mL), the organic phase was dried (Na_2SO_4) and the solvents evaporated in a rotary evaporator to yield an orange oil. Pale yellow crystals were grown from cold hexanes, and the product was further purified by sublimation (115 °C, 0.005 mBar) to give a white crystalline powder consisting of the analytically pure product. Yield = 3.2 g (54%). Elemental Analysis for $C_{14}H_{15}BO_5$ (%): expected C, 60.03; H, 6.61; N, 0.00; found C, 59.97; H, 6.45; N, <0.05.

^{1}H NMR of **BPin-DMP** ($CDCl_3$, 25 °C, 400.2 MHz): δ 8.15 (s, 1H, Ar-*H*); 7.95 (dd, J_1 = 1.0 Hz, J_2 = 5.0 Hz, 1H, Ar-*H*); 7.69 (d, J = 5.2 Hz, 1H, Ar-*H*); 3.91 (s, 3H, –OMe), 3.90 (s, 3H, –OMe), 1.34 (s, 12H, BPin). ^{13}C{^{1}H} NMR of **BPin-DMP** ($CDCl_3$, 25 °C, 100.6 MHz): δ 168.3 and 168.2 (*C*=O), 137.5, 135.2, 134.3, 131.1, and 128.1 (Ar-*C*), 84.6 (O–*C*), 52.8 (CO_2*Me*), 52.7 (CO_2*Me*), 25.0 (–Me). ^{11}B{^{1}H} NMR of **BPin-DMP** ($CDCl_3$, 25 °C, 128.4 MHz): δ 30.1.

FT-IR of **BPin-DMP** (25 °C, cm^{-1}): 2964, 1724, 1610, 1565, 1492, 1455, 1437, 1415, 1398, 1375, 1355, 1328, 1288, 1262, 1213, 1197, 1169, 1148, 1126, 1099, 1074, 975, 961, 923, 879, 865, 852, 822, 803, 796, 766, 710, 682, 670, 660, 575, 523, 510.

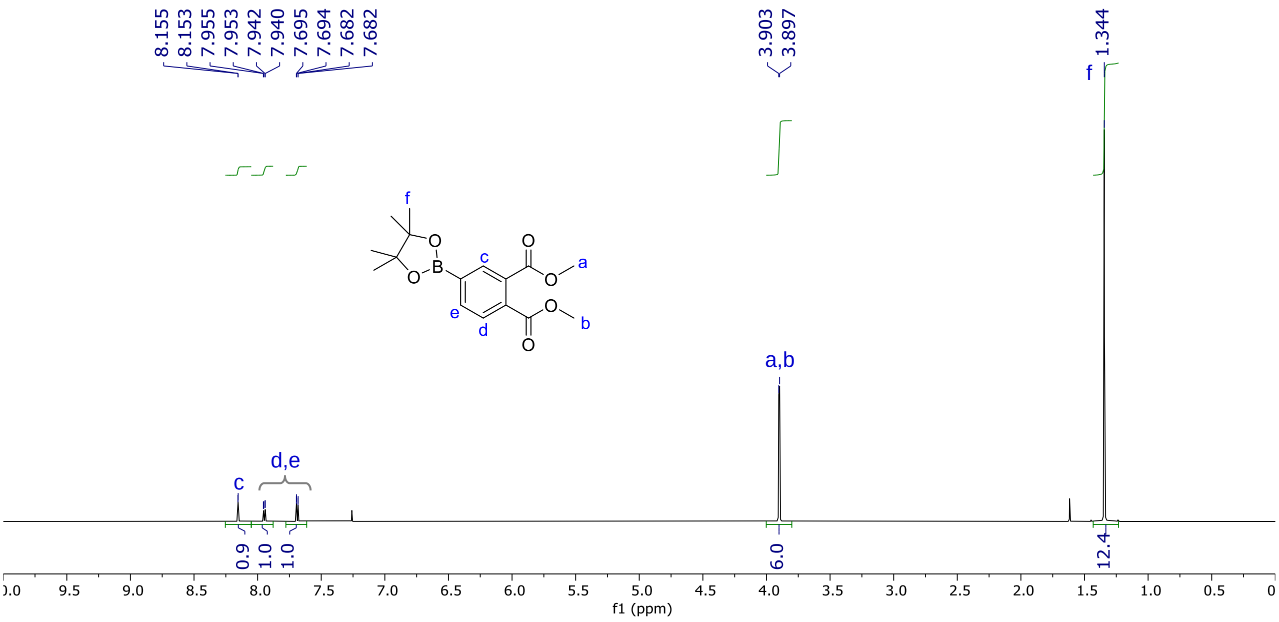

Figure S5. ^{1}H NMR ($CDCl_3$, 25 °C, 400.2 MHz) spectrum of **BPin-DMP**.

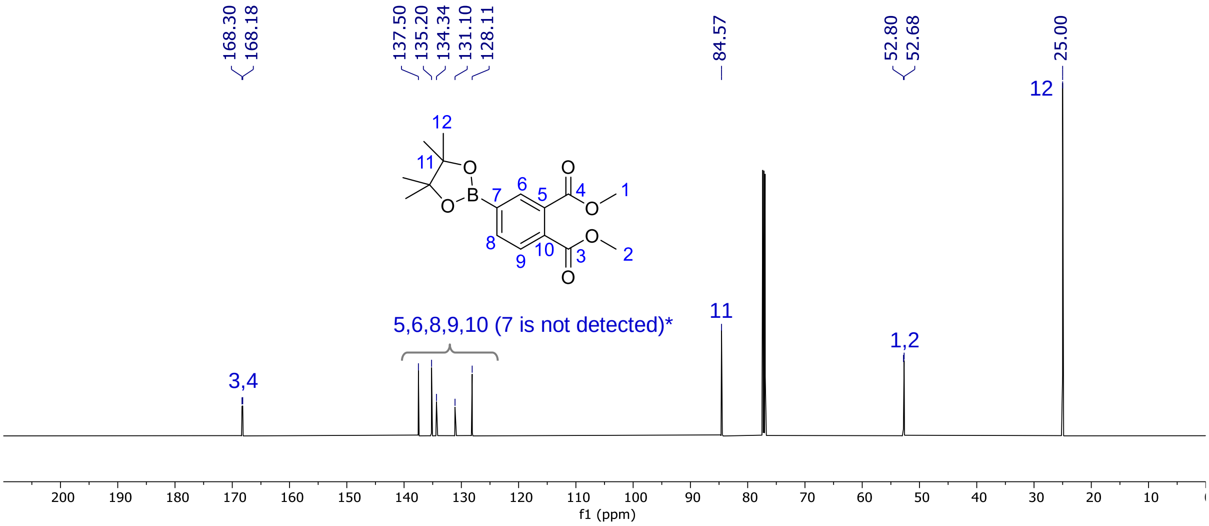

Figure S6. ^{13}C NMR ($CDCl_3$, 25 °C, 100.6 MHz) spectrum of **BPin-DMP**. (* due to B–C coupling)

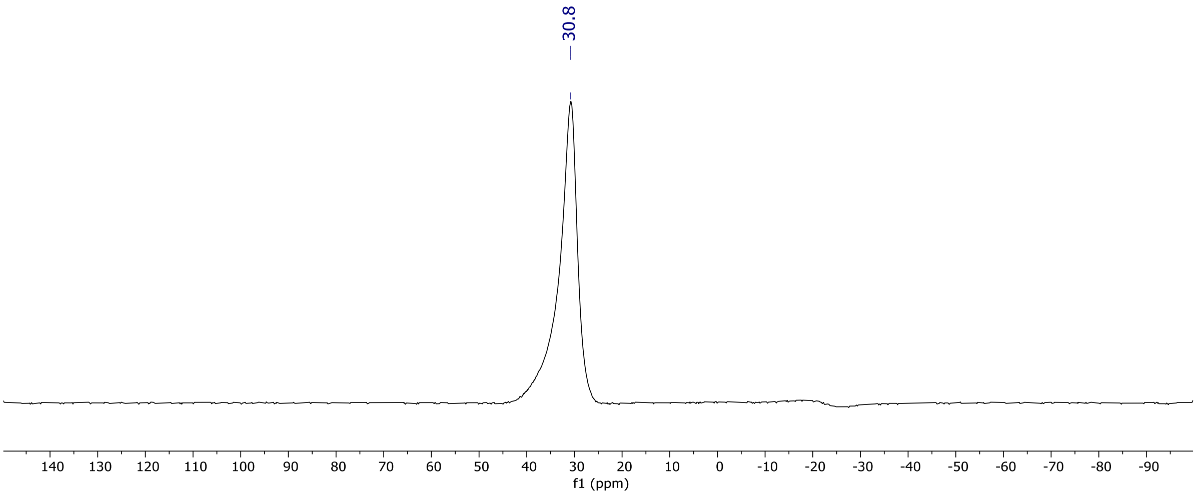

Figure S7. $^{11}B\{^{1}H\}$ NMR ($CDCl_3$, 25 °C, 128.4 MHz) spectrum of **BPin-DMP**.

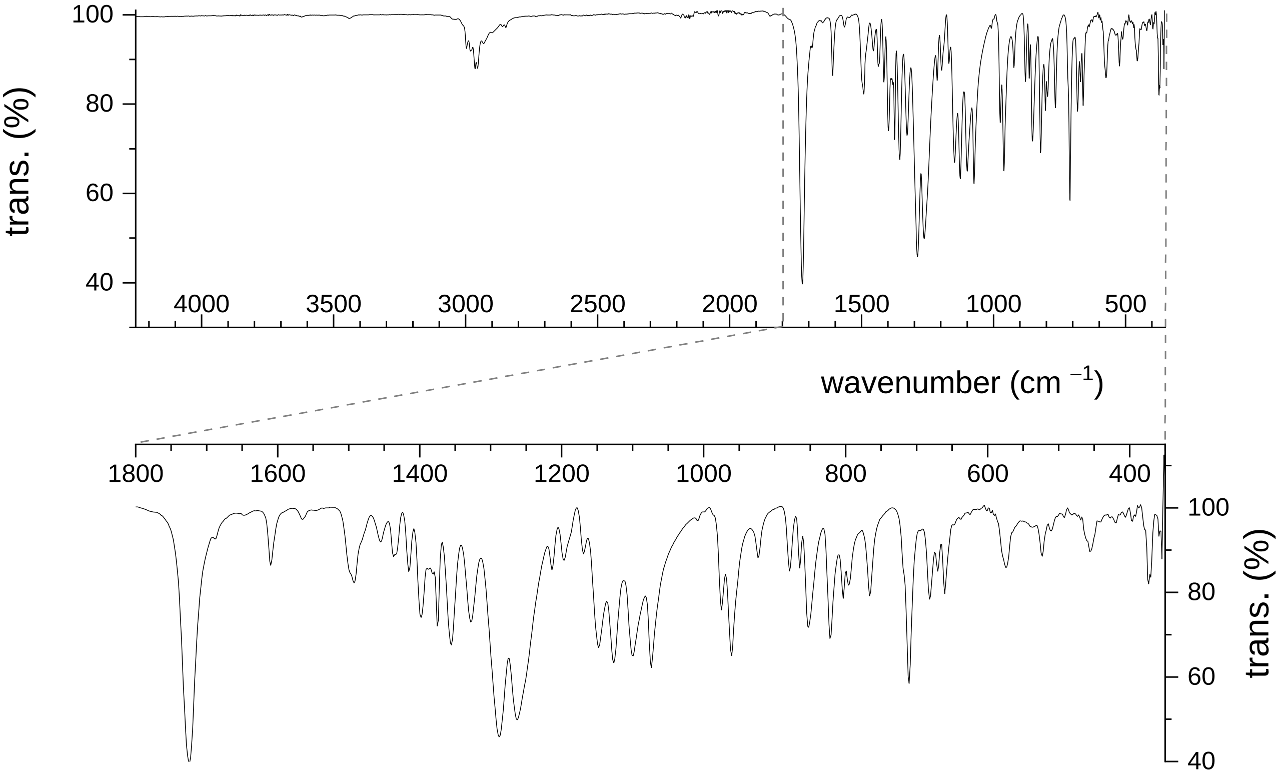

Figure S8. FT-IR spectrum at 25 °C of **BPin-DMP** (top) and zoom in the 1800–350 cm^{-1} region (bottom).

2.3. Synthesis of dimethyl phthalate boronic acid, [B(OH)$_2$-DMP]

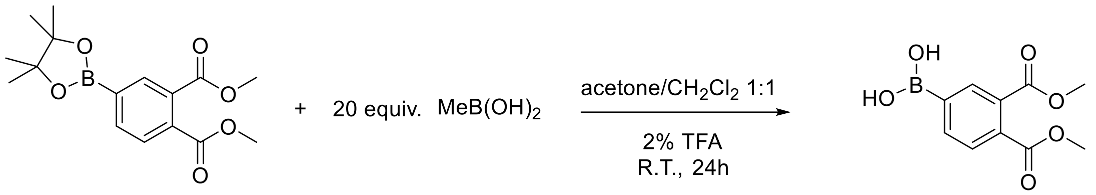

On the benchtop and under air, a 40 mL reaction vial containing a magnetic stir bar was loaded with dimethyl phthalate pinacolboronate ester, **BPin-DMP**, (320 mg, 1.0 mmol) and methylboronic acid (1.2 g, 20.0 mmol). The solids were dissolved in a solution of trifluoroacetic acid (10 mL, 2% in acetone/CH_2Cl_2 1:1) and stirred at room temperature for 24 h. After this time, the volatiles were removed using a rotary evaporator at 40 °C. The residue was redissolved in acetone (3 mL, containing 100 µL of H_2O) and evaporated under vacuum. The boronic acid was crystallized from acetone/hexane mixture. Yield = 180 mg (76%).

^{1}H NMR of **B(OH)$_2$-DMP** [$(CD_3)_2CO$, 25 °C, 400.2 MHz]: δ 8.22 (s, 1H, Ar-*H*); 8.09 (dd, J_1 = 1.2 Hz, J_2 = 7.6 Hz, 1H, Ar-*H*); 7.70 (d, *J* = 7.6 Hz, 1H, Ar-*H*); 7.55 (s, 2H, B-OH), 3.86 (s, 3H, –OMe), 3.86 (s, 3H, –OMe). ^{13}C{^{1}H} NMR of **B(OH)$_2$-DMP** [$(CD_3)_2CO$, 25 °C, 100.6 MHz]: δ 168.7 and 168.6 (*C*=O), 137.7, 135.2, 134.7, 131.9, and 128.6 (Ar-*C*), 52.8 (CO_2*Me*), 52.7 (CO_2*Me*). ^{11}B{^{1}H} NMR of **B(OH)$_2$-DMP** [$(CD_3)_2CO$, 25 °C, 128.4 MHz]: δ 28.3.

FT-IR of **B(OH)$_2$-DMP** (25 °C, cm^{-1}): 3362, 3032, 2999, 2948, 2842, 1728, 1698, 1612, 1567, 1498, 1454, 1234, 1409, 1369, 1344, 1307, 1274, 1188, 1164, 1131, 1079, 1061, 1042, 968, 916, 856, 819, 790, 773, 706, 676, 668, 615, 573, 526.

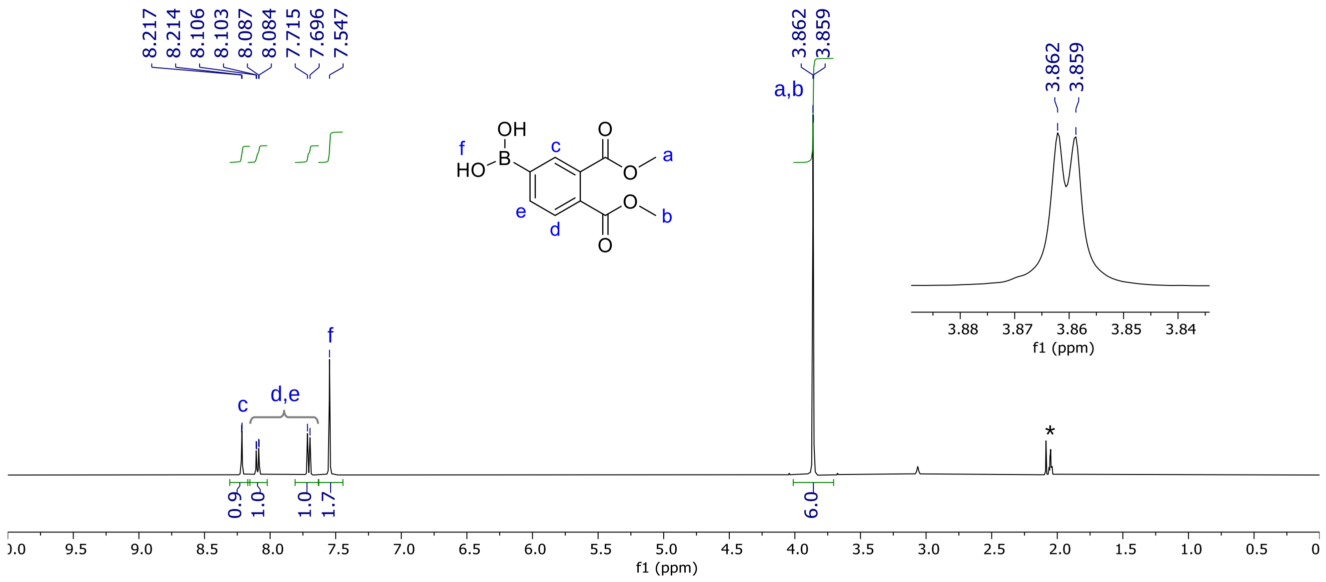

Figure S9. ^{1}H NMR [$(CD_3)_2CO$, 25 °C, 400.2 MHz] spectrum of **B(OH)$_2$-DMP**. Inserts shows the two singlets in the –OME region. (* = residual acetone).

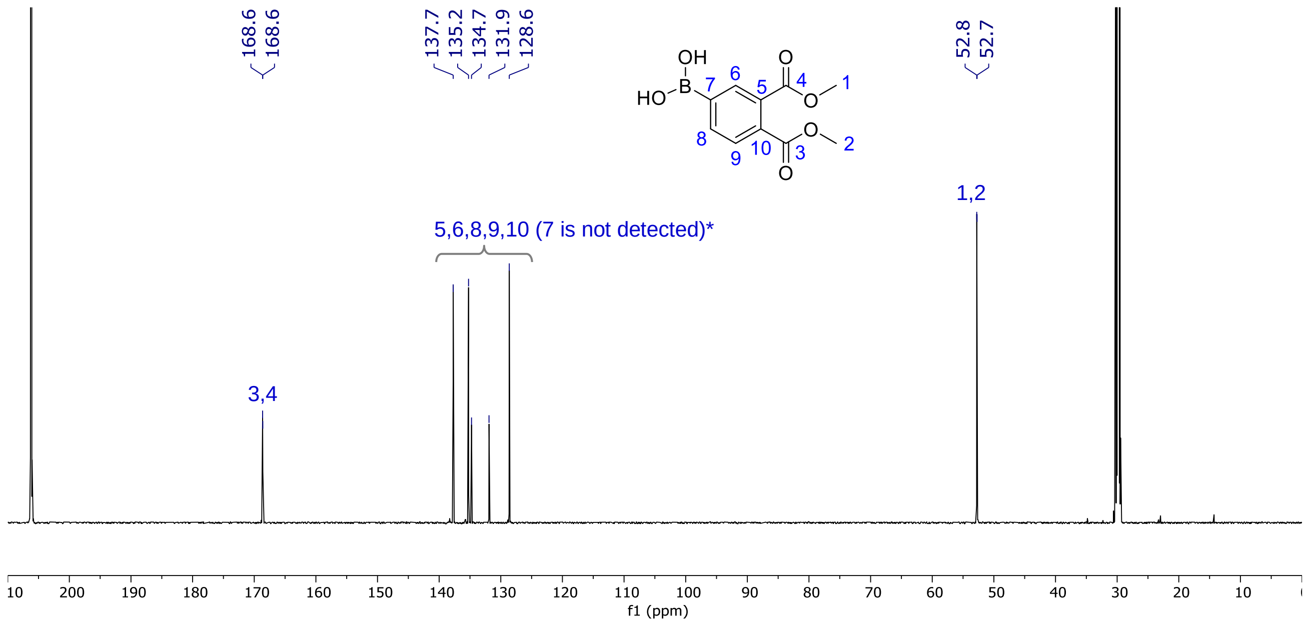

Figure S10. ^{13}C NMR [$(CD_3)_2CO$, 25 °C, 100.6 MHz] spectrum of **B(OH)$_2$-DMP**. (* due to B–C coupling)

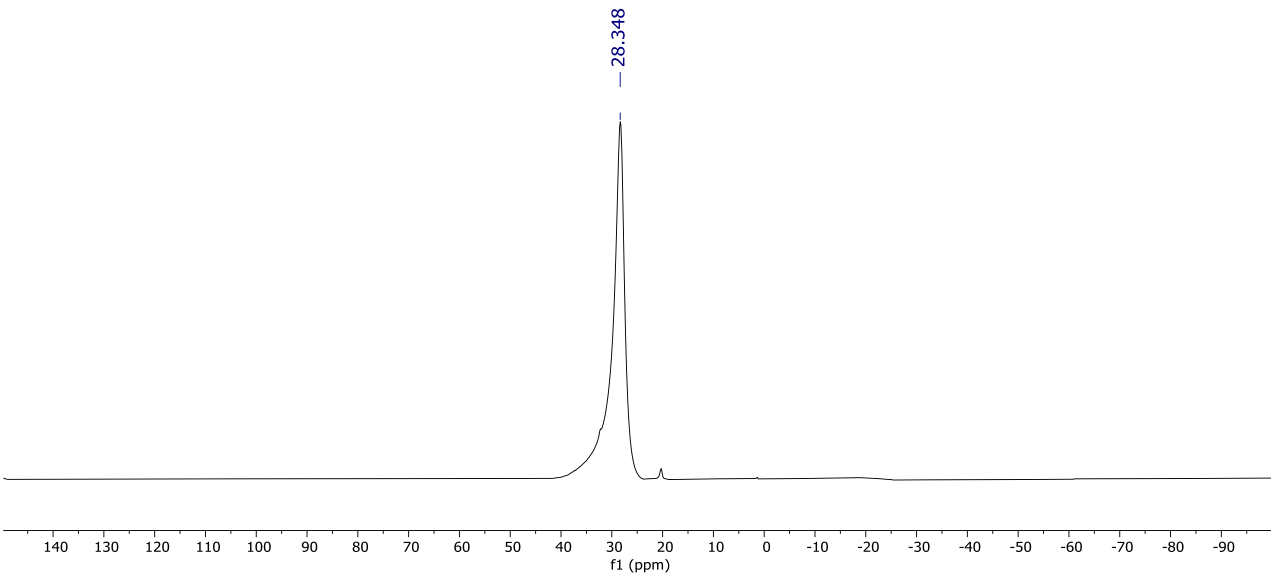

Figure S11. $^{11}B\{^{1}H\}$ NMR [$(CD_3)_2CO$, 25 °C, 128.4 MHz) spectrum of **B(OH)$_2$-DMP**.

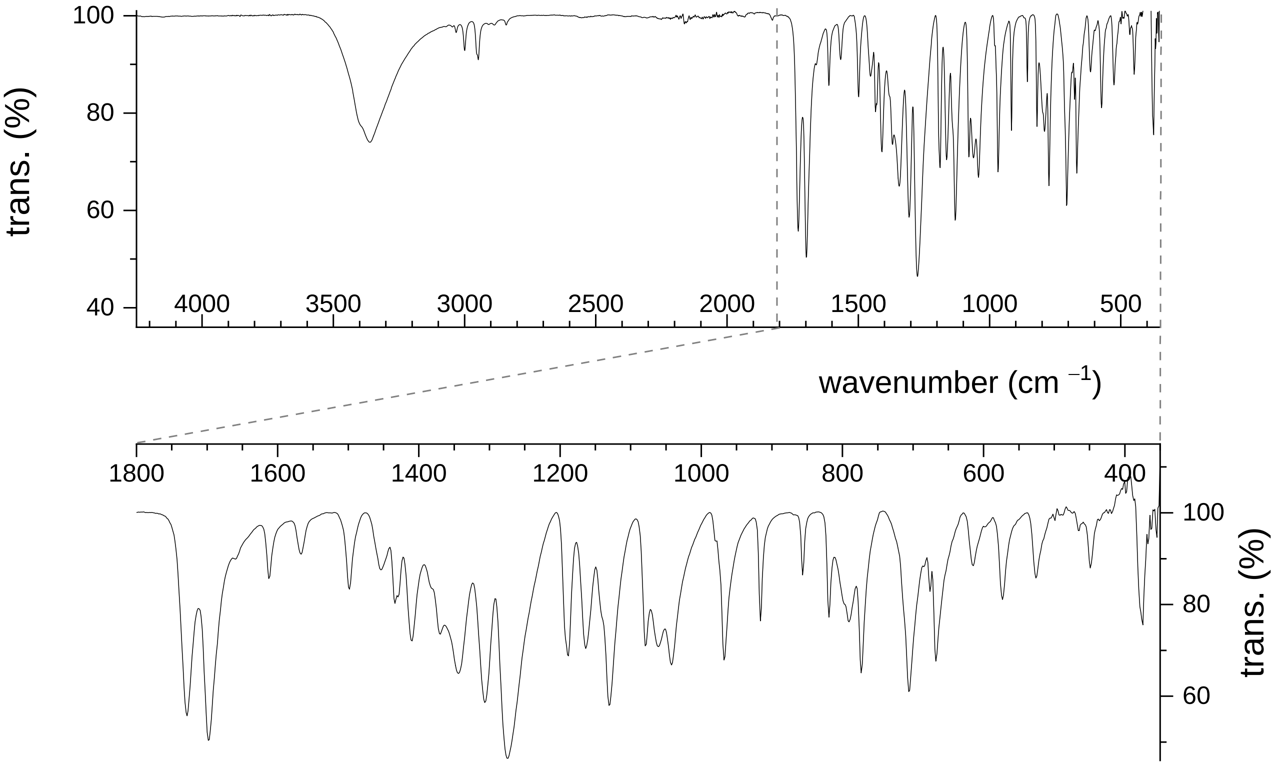

Figure S12. FT-IR spectrum at 25 °C of **B(OH)$_2$-DMP** (top) and zoom in the 1800–350 cm^{-1} region (bottom).

3. ROCOP of BPin-PA with Epoxides

3.1. Typical BPin-PA/epoxide ROCOP.

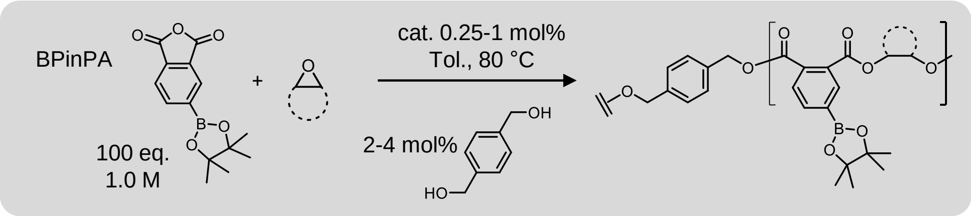

Inside a N_2-filled glovebox, an oven-dried 7 mL reaction vial, with a Teflon-lined screw cap, was loaded with a magnetic stir bar, 1,4-BDM (2.8 or 5.5 mg, 0.02 or 0.04 mmol, 2 or 4 equiv.) and BPin-PA (274 mg, 1.00 mmol, 100 equiv.). Then, epoxide (1.50 or 4.00 mmol, 150 or 400 equiv.) was injected and a solution of [ZnMg] (10 mg, 0.01 mmol, 1 equiv.) or P1-t-Bu (2 mg, 0.01 mmol, 1 equiv.), in toluene, was added so that the final BPin-PA concentration was maintained at 1.00 M. For reactions with [AlK] catalyst, [AlK] (5 mg, 0.01 mmol, 1 equiv.) and BPin-PA (274 mg, 1.00 mmol, 100 equiv.) were loaded onto the reaction vial; then, a solution of 1,4-BDM (6 mg, 0.04 mmol, 4 equiv.) in epoxide (5.00 mmol, 2000 equiv.) was added. Finally, this mixture was further diluted with toluene until the concentration of BPin-PA reached 1.00 M. In all cases, the vial was sealed and transferred to an oil bath, which was preheated to the desired temperature. At appropriate timed intervals, the polymerization was stopped by submersion in an acetone/dry ice bath and the vial was taken into the glovebox, where an aliquot was taken for NMR spectroscopy analysis to determine BPin-PA conversion. After the appropriate time, the reaction was opened to air to quench the catalyst, diluted with dichloromethane (1 mL), and precipitated into excess methanol (50 mL). The solids thus formed were decanted, washed with additional methanol (3 × 50 mL), filtered and dried under vacuum, at 60 °C, until constant weight (yields varied between ca. 70-90% due to losses during manipulation).

P(BPin-PA/CHO). ^{1}H NMR ($CDCl_3$, 25 ◦C, 499.9 MHz): δ 8.13–7.99 (bs, 1H, Ar-*H*); 7.87 (bs, 1H, Ar-*H*); 7.69–7.39 (bs, 1H, Ar-*H*); 7.39 (s, Ar-*H* 1,4-BDM end-group); 5.24–5.07 (bs, 2H, –OC*H*–); 2.35–2.16 (bs, 2H, –CH_2–); 1.74 (bs, 2H, –CH_2–); 1.53 (bs, 2H, –CH_2–); 1.40 (bs, 2H, –CH_2–); 1.27 (bs, 12H, BPin). ^{11}B{^{1}H} NMR ($CDCl_3$, 25 ◦C, 160.4 MHz): δ 31.0. FT-IR (25 °C, cm^{-1}): 2976, 2938, 2866, 1721, 1608, 1561, 1497, 1450, 1391, 1360, 1321, 1284, 1252, 1144, 1124, 1099, 1061, 1025, 992, 964, 918, 852, 795, 763, 711, 668.

P(BPin-PA/vCHO). ^{1}H NMR ($CDCl_3$, 25 ◦C, 499.9 MHz): δ 8.07 (bs, 1H, Ar-*H*); 7.92 (bs, 1H, Ar-*H*); 7.73–7.64 (bs, 1H, Ar-*H*); 7.42 (s, Ar-*H* 1,4-BDM end-group); 5.80 (bs, 1H, –C*H*=C); 5.36–4.96 (m, 4H, –OC*H*– and C=CH_2); 2.48 (bs, 1H, –C*H*–); 1.99 (bs, 4H, –CH_2–); 1.66–1.60 (bs, 2H, –CH_2–); 1.32 (bs, 12H, BPin). ^{11}B{^{1}H} NMR ($CDCl_3$, 25 ◦C, 160.4 MHz): δ 30.1. FT-IR (25 °C, cm^{-1}): 2976, 2932, 2863, 1724, 1640, 1608, 1561, 1497, 1391, 1360, 1325, 1247, 1142, 1122, 1098, 1061, 994, 964, 914, 852, 792, 763, 710, 663.

P(BPin-PA/PO). ^{1}H NMR ($CDCl_3$, 25 ◦C, 499.9 MHz): δ 8.14–8.08 (bs, 1H, Ar-*H*); 7.92 (bs, 1H, Ar-*H*); 7.75–7.63 (bs, 1H, Ar-*H*); 7.38 (s, Ar-*H* 1,4-BDM end-group); 5.41 (bs, 1H, –OC*H*–); 4.41 (bs, 2H, –CH_2–); 1.38 (bs, 3H, –CH_3); 1.30 (bs, 12H, BPin). ^{11}B{^{1}H} NMR ($CDCl_3$, 25 ◦C, 160.4 MHz): δ 31.3. FT-IR (25 °C, cm^{-1}):

2979, 2935, 1724, 1610, 1561, 1497, 1452, 1390, 1360, 1327, 1244, 1141, 1125, 1098, 1058, 993, 964, 921, 851, 791, 764, 709, 668, 578.

P(BPin-PA/AGE). ^{1}H NMR ($CDCl_3$, 25 ◦C, 499.9 MHz): δ 8.14–8.10 (bs, 1H, Ar-*H*); 7.92 (bs, 1H, Ar-*H*); 7.77–7.64 (bs, 1H, Ar-*H*); 7.38 (s, Ar-*H* 1,4-BDM end-group); 5.85 (bs, 1H, –C*H*=C); 5.50 (bs, 1H, –OC*H*–); 5.28–5.13 (bs, 2H, C=CH_2); 4.67–4.53 (bs, 2H, O–CH_2); 4.01 (bs, 2H, O–CH_2); 3.73 (bs, 2H, O–CH_2); 1.30 (bs, 12H, BPin). ^{11}B{^{1}H} NMR ($CDCl_3$, 25 ◦C, 160.4 MHz): δ 30.7. FT-IR (25 °C, cm^{-1}): 2979, 2935, 2863, 1725, 1646, 1610, 1561, 1497, 1448, 1391, 1358, 1327, 1245, 1140, 1124, 1093, 1051, 992, 964, 926, 850, 791, 763, 709, 664, 576.

3.2. Kinetics of BPin-PA/epoxide ROCOP and PA/epoxide ROCOP using [ZnMg].

Inside a glovebox, a 7 mL glass vial, with a Teflon-lined screw cap, was loaded with a magnetic stirrer and [ZnMg] (10 mg, 0.01 mmol, 1 equiv.). Then, 1,4-BDM (6 mg, 0.04 mmol, 4 equiv.) and BPin-PA or PA (1.00 mmol, 100 equiv.) were added along with epoxide (1.3 mL of CHO or vCHO). The vial was sealed and transferred to an oil bath, which was preheated to 80 °C. At appropriate time intervals, the polymerization was stopped by submersion into an acetone/dry ice bath and the reaction vial was taken into the glovebox, where an aliquot was taken for analysis by NMR spectroscopy to determine BPin-PA or PA conversion. Specifically, the amount of unreacted BPin-PA at a given time *t* was determined by integration of the peaks for BPin-PA (centered at 8.30 ppm for one aromatic signal) and BPin polyester (centered at 7.40 ppm for one aromatic signal) according to the percent of unreacted BPin-PA = ($\mathbf{A}_{8.30}/\mathbf{A}_{7.40}$) × 100, where $\mathbf{A}_{8.30}$ is the total integral for the peak centered at 8.30 ppm and $\mathbf{A}_{7.40}$ is the total integral for the peak centered at 7.40 ppm. Conversely, the percent of unreacted PA at a given time *t* was determined by integration of the peaks for PA (centered at 8.02 ppm for one aromatic signal) and PA polyester (centered at 7.67 ppm for one aromatic signal) according to the percent of unreacted PA = ($\mathbf{A}_{8.02}/\mathbf{A}_{7.67}$) × 100, where $\mathbf{A}_{8.02}$ is the total integral for the peak centered at 8.02 ppm and $\mathbf{A}_{7.67}$ is the total integral for the peak centered at 7.67 ppm. The apparent rate constants (k_{app}) were extracted from linear fit plots of $[\text{BPin-PA}]_t$ or $[\text{PA}]_t$ vs time *t*.

The aliquots were also analysed by gel permeation chromatography to determine M_n and $Đ$ values.

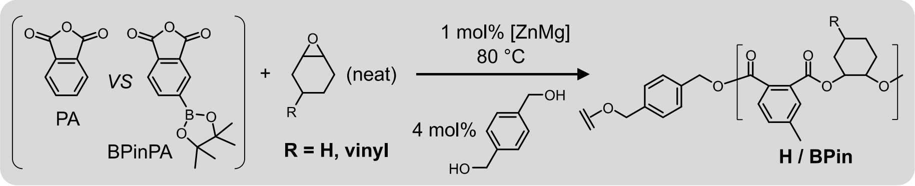

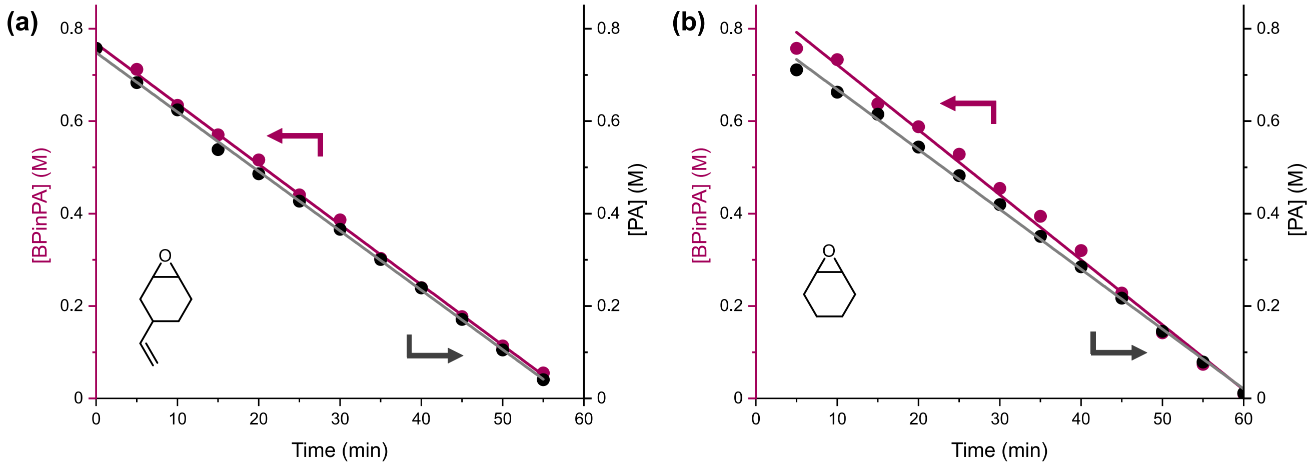

Figure S13. Plots of [BPin-PA] (purple) and [PA] (black) versus time (min) in the ROCOP with vCHO (a) and CHO (b), using the [ZnMg] catalyst, as determined by ^{1}H NMR analysis of reaction aliquots. Conditions: $[\text{MgZn}]_0$ = 7.6 mM, neat epoxide, 80 °C, [anhydride]/[1,4-BDM]/[ZnMg] = 100:4:1.

Table S1. Kinetic data for the ROCOP of BPin/PA and CHO/vCHO with [ZnMg] catalyst. [a]

anhydride	epoxide	TOF (h^{-1})	k_{obs} (mM h^{-1})
BPin-PA	CHO	114	14.1 ± 0.3
PA	CHO	106	13.2 ± 0.2
PA	vCHO	103	13.1 ± 0.1
BPin-PA	vCHO	102	12.9 ± 0.1

[a] Conditions: $[ZnMg]_0$ = 7.6 mM, [anhydride]/[1,4-BDM]/[ZnMg] = 100:4:1, neat epoxide, 80 °C.

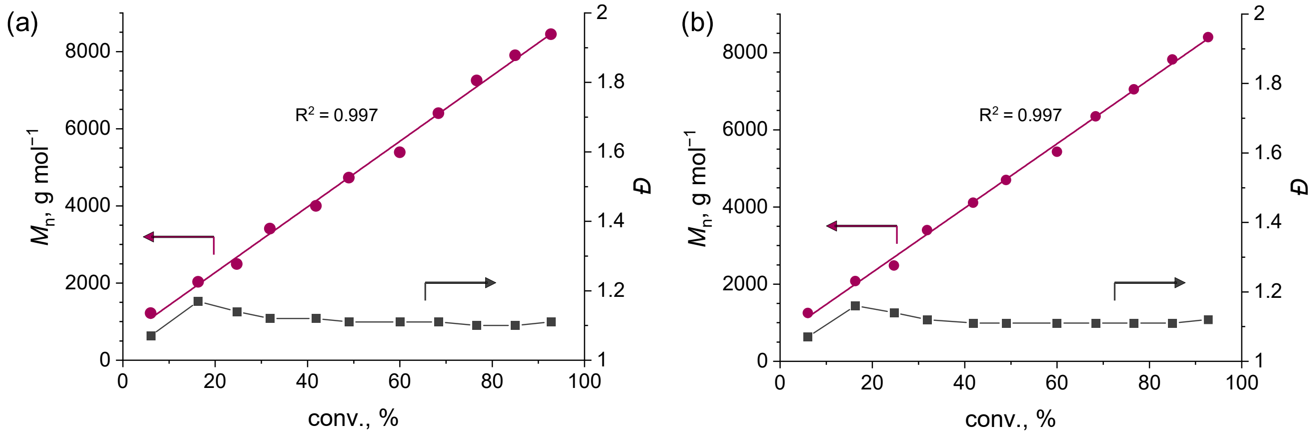

Figure S14. Plots of M_n (g mol $^{-1}$) and $Đ$ of P(BPin-PA/vCHO) vs BPin-PA conversion: (a) M_n using the RI detector and (b) M_n using the UV detector. Conditions: $[MgZn]_0$ = 7.6 mM in neat vCHO, 80 °C, [BPin-PA]/[1,4-BDM]/[cat.] = 100:4:1.

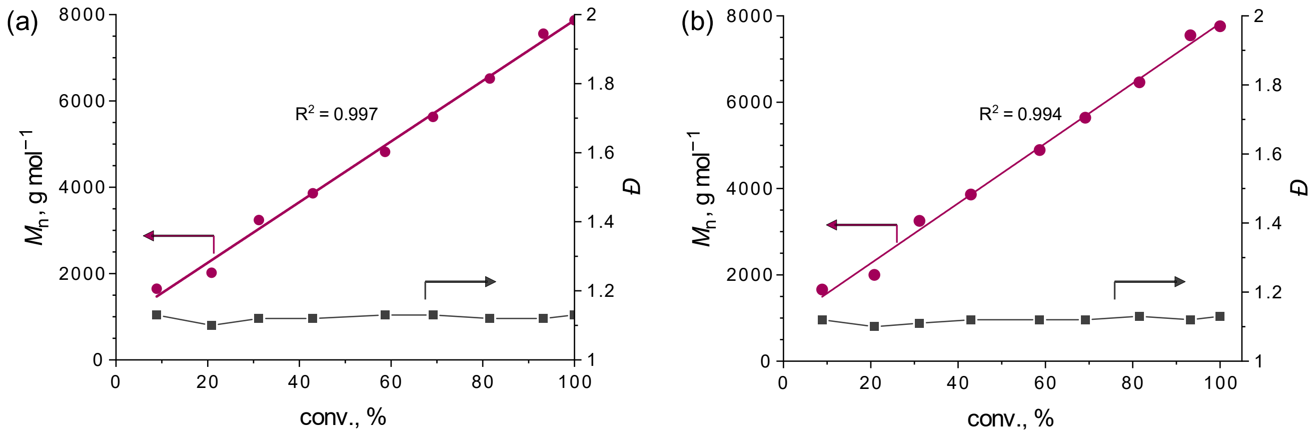

Figure S15. Plots of M_n (g mol $^{-1}$) and $Đ$ of P(BPin-PA/CHO) vs BPin-PA conversion, as determined by GPC: (a) RI detector and (b) UV detector. Conditions: $[MgZn]_0$ = 7.6 mM in neat CHO, [BPin-PA]/[1,4-BDM]/[cat.] = 100:4:1.

3.3. NMR Spectra of BPin-PA Polyesters

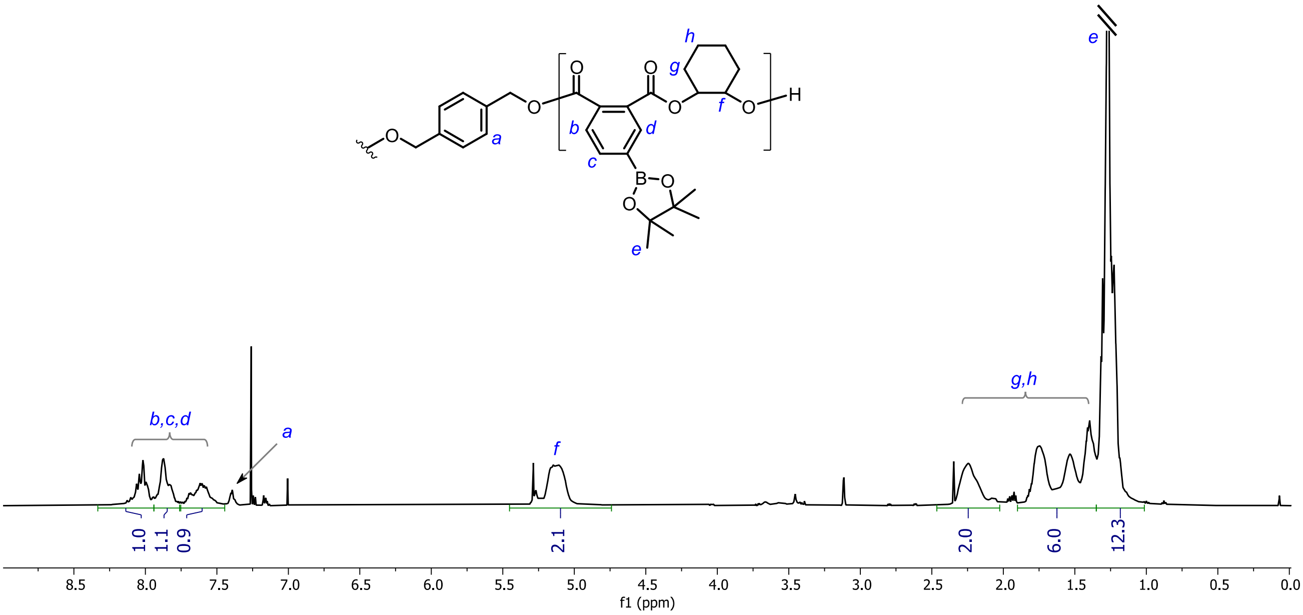

Figure S16. ^{1}H NMR ($CDCl_3$, 25 °C, 499.9 MHz) spectrum of isolated P(BPin-PA/CHO).

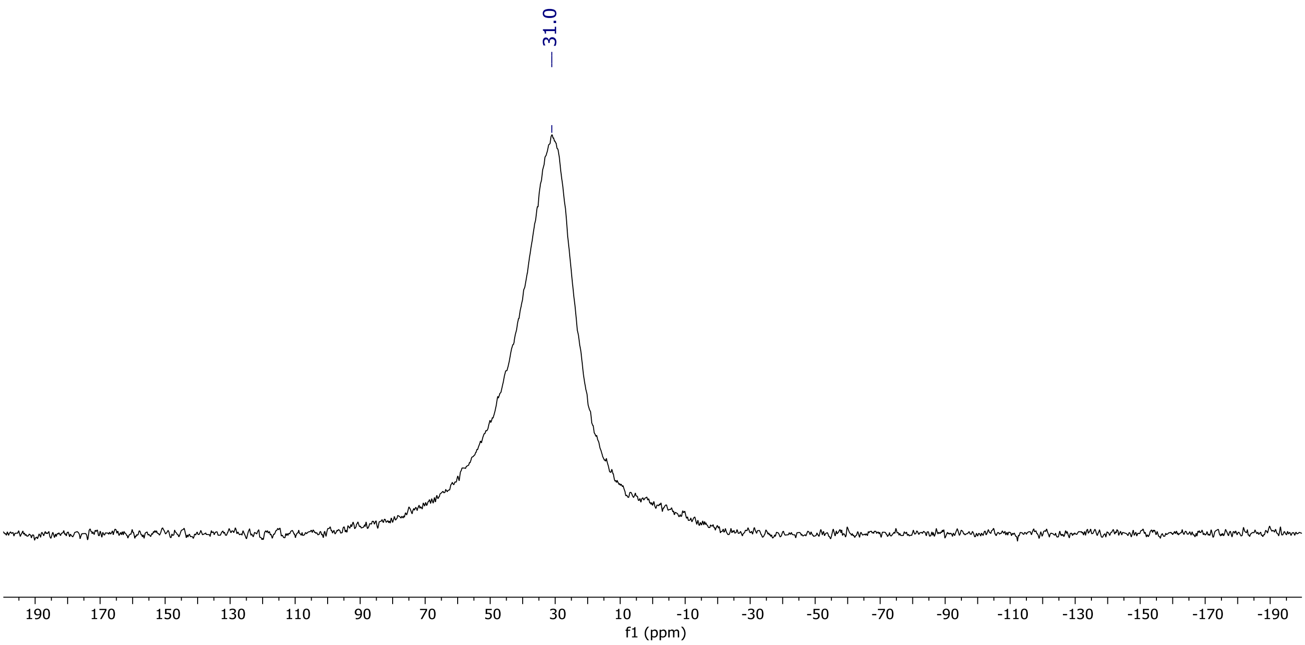

Figure S17. $^{11}B\{^{1}H\}$ NMR ($CDCl_3$, 25 °C, 160.4 MHz) spectrum of isolated P(BPin-PA/CHO).

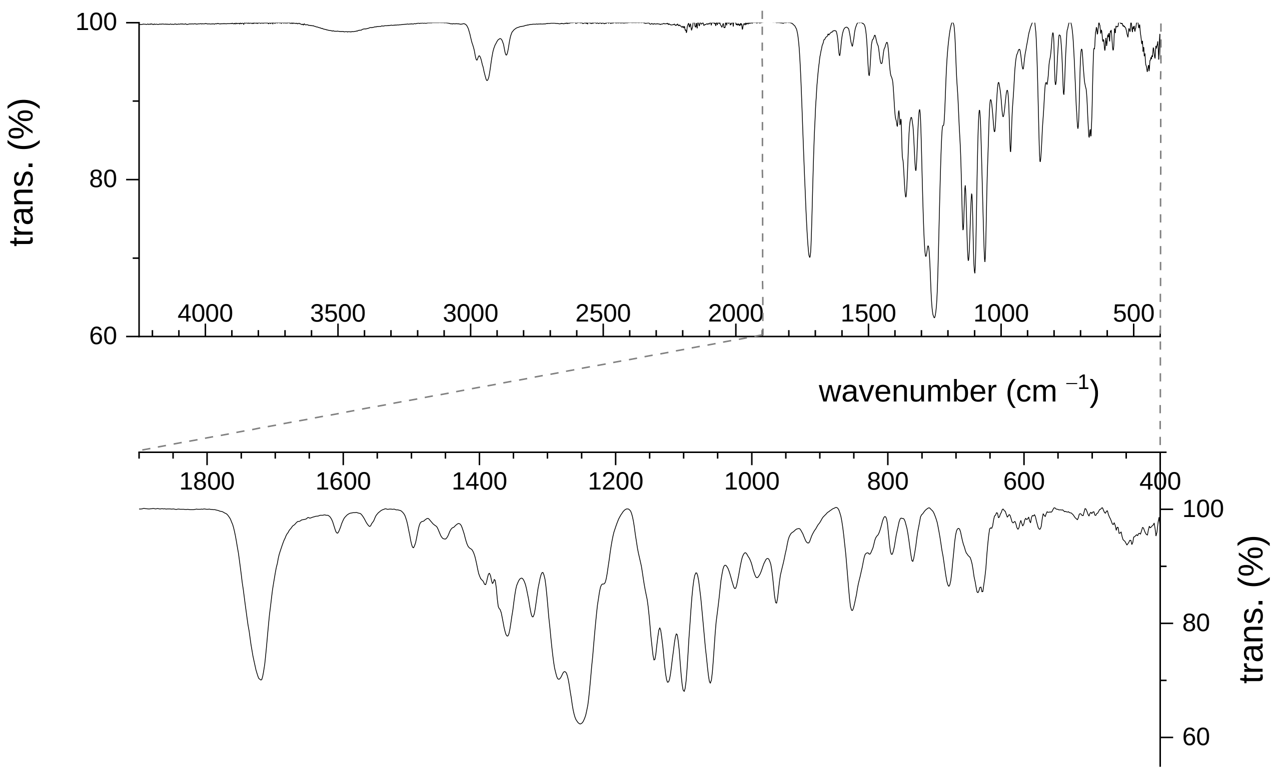

Figure S18. FT-IR spectrum at 25 °C of P(BPin-PA/CHO) (top) and zoom in the 1900–400 cm^{-1} region (bottom).

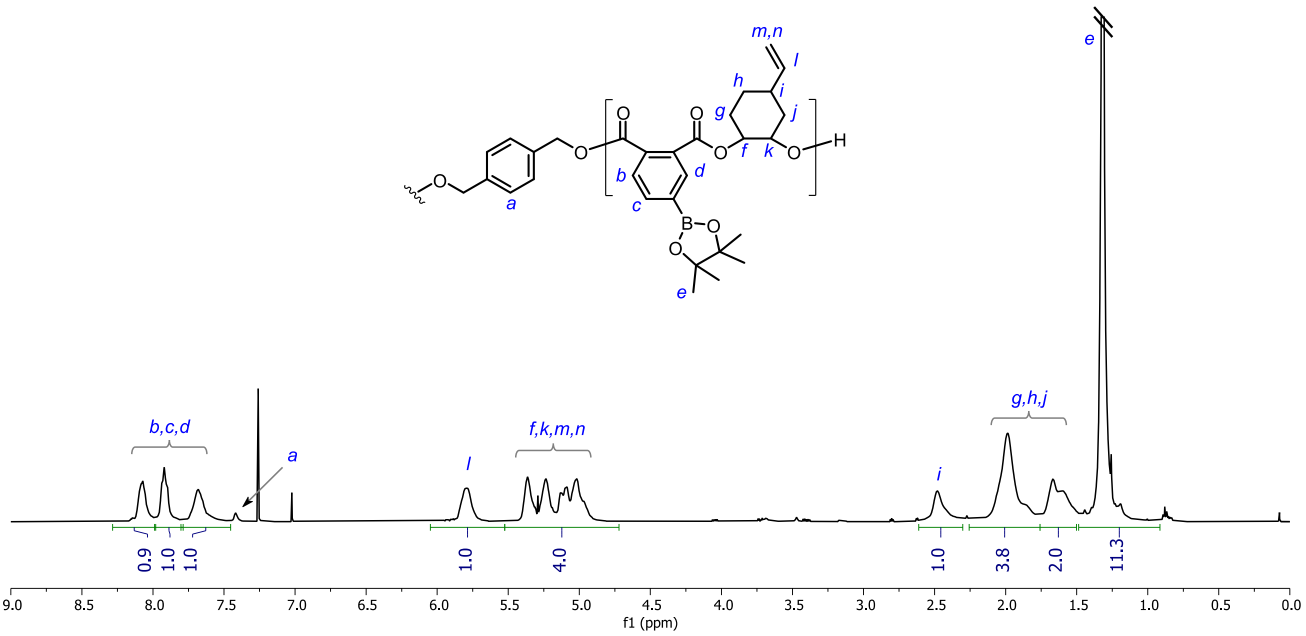

Figure S19. ^{1}H NMR ($CDCl_3$, 25 °C, 499.9 MHz) spectrum of isolated P(BPin-PA/vCHO).

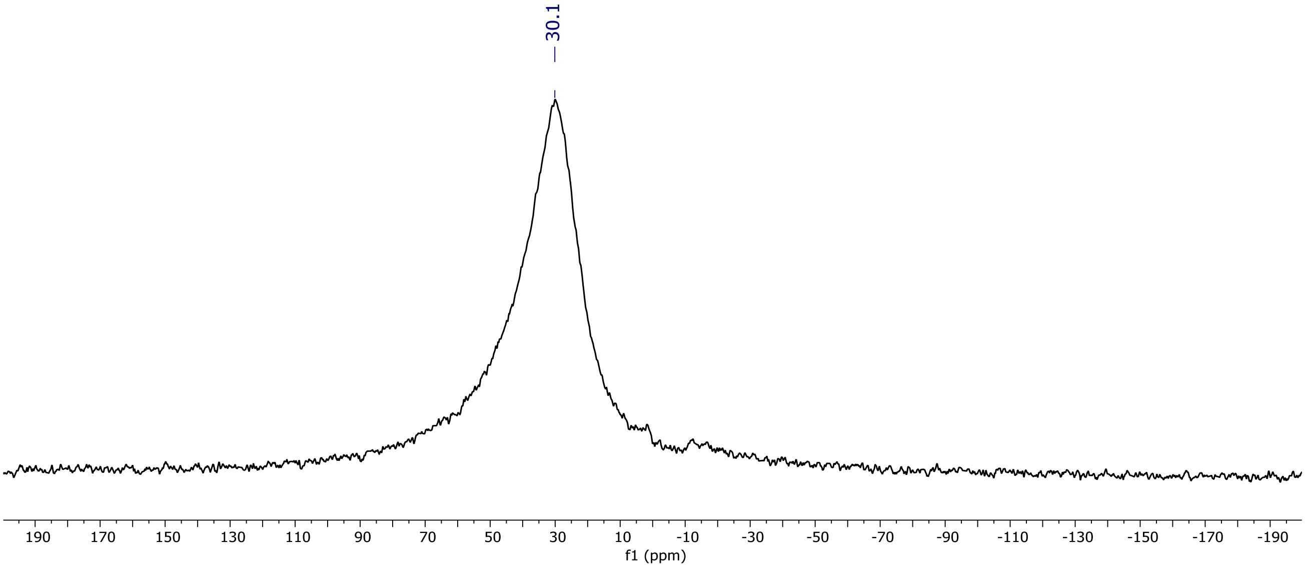

Figure S20. $^{11}B\{^{1}H\}$ ($CDCl_3$, 25 °C, 160.4 MHz) spectrum of isolated P(BPin-PA/vCHO).

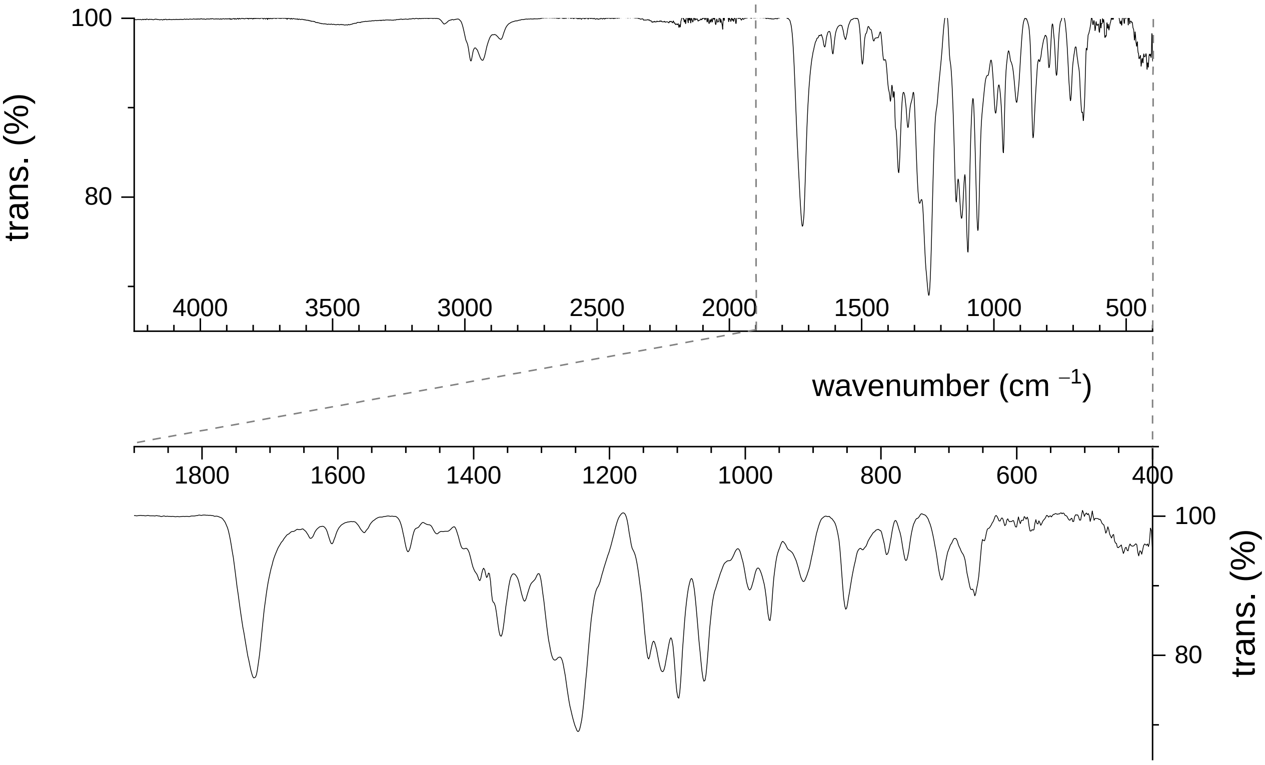

Figure S21. FT-IR spectrum at 25 °C of P(BPin-PA/vCHO) (top) and zoom in the 1900–400 cm^{-1} region (bottom).

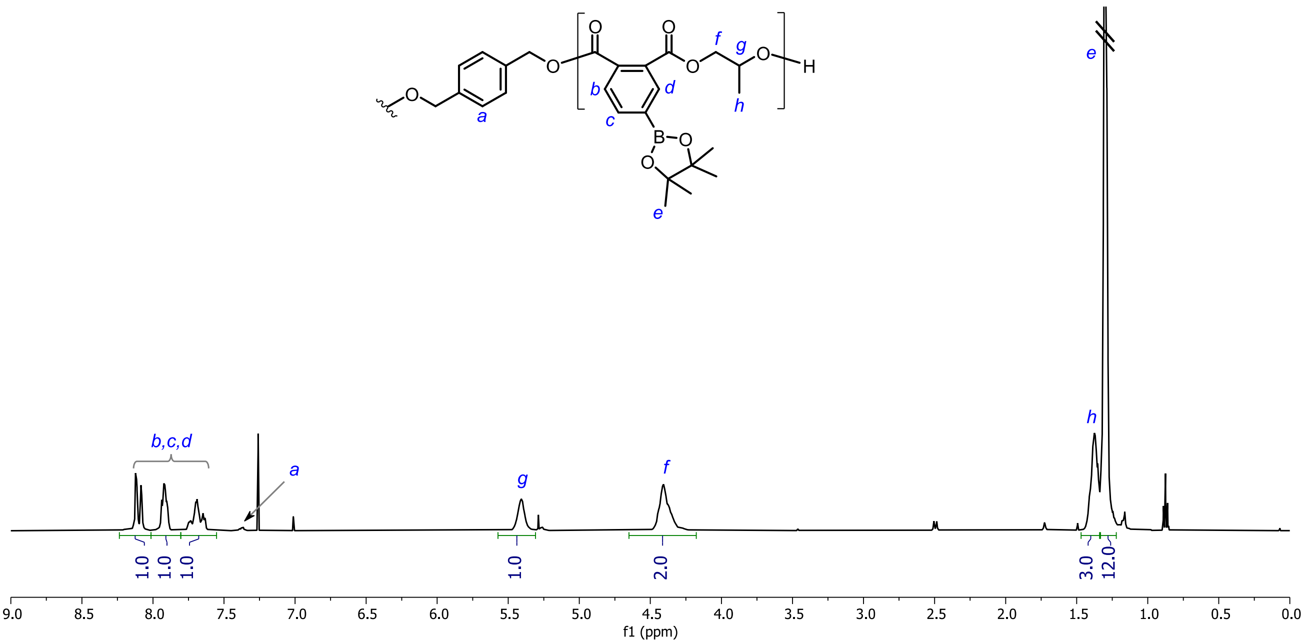

Figure S22. ^{1}H NMR ($CDCl_3$, 25 °C, 499.9 MHz) spectrum of isolated P(BPin-PA/PO).

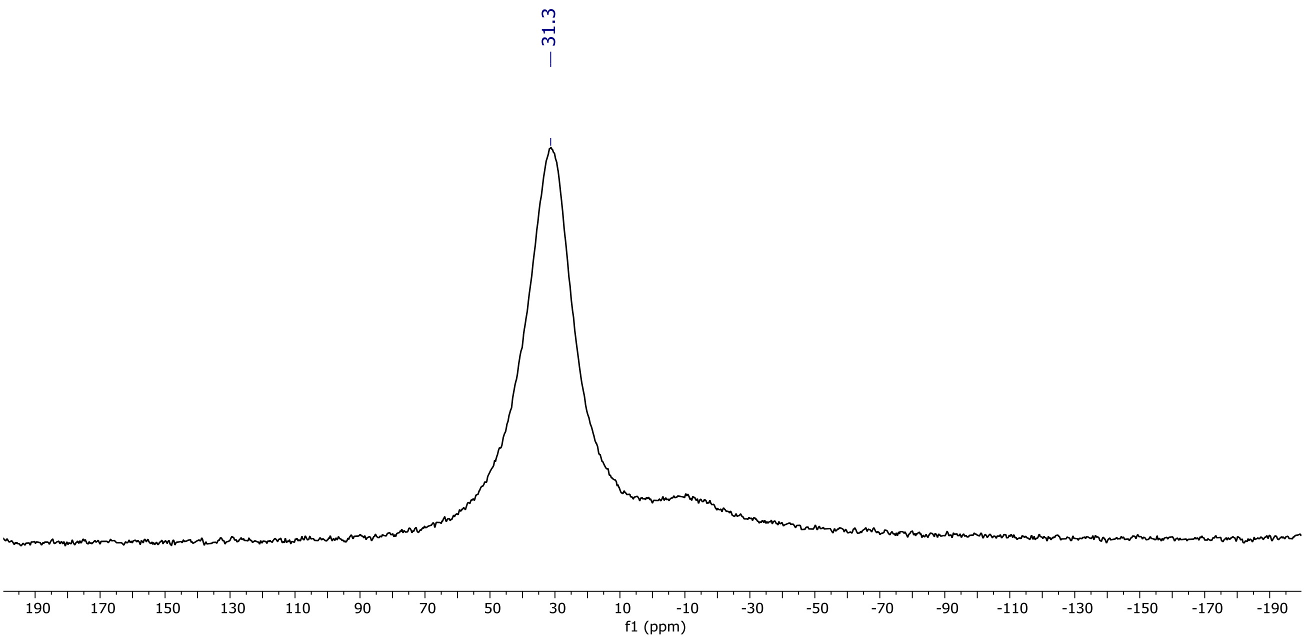

Figure S23. $^{11}B\{^{1}H\}$ ($CDCl_3$, 25 °C, 160.4 MHz) spectrum of isolated P(BPin-PA/PO).

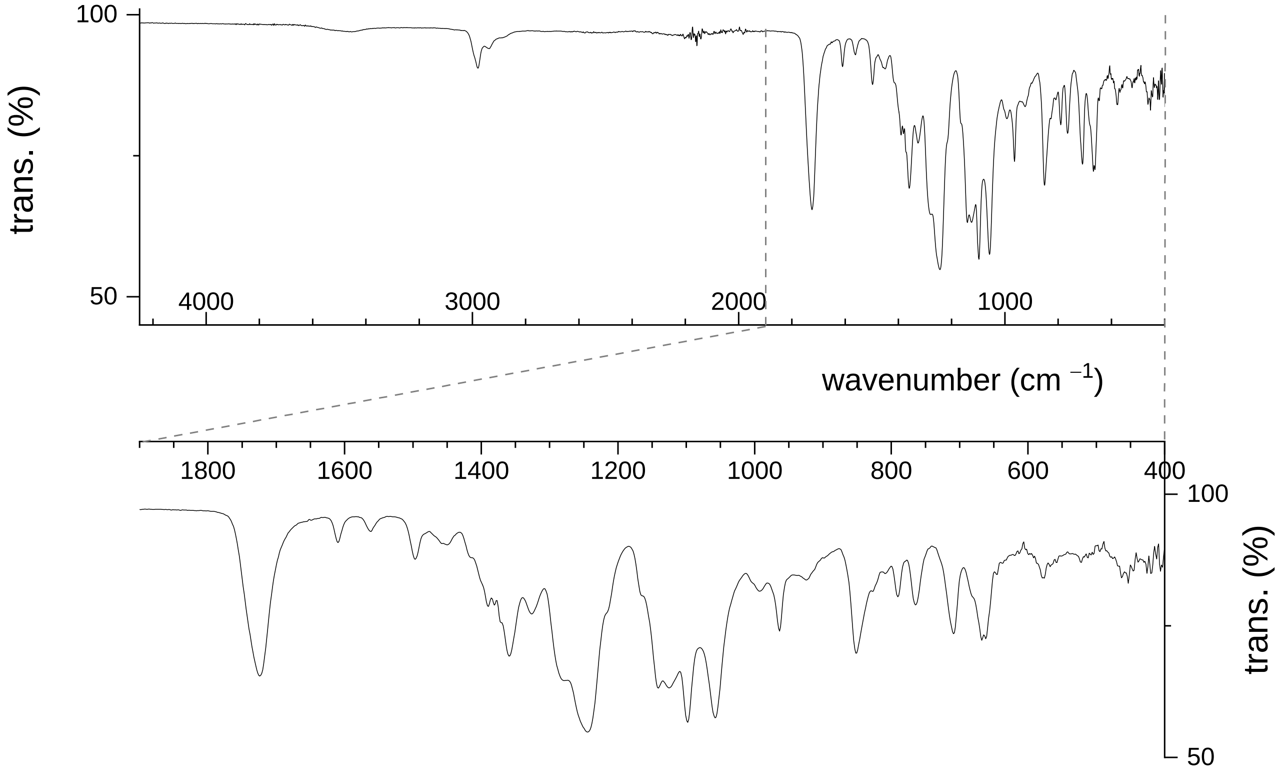

Figure S24. FT-IR spectrum at 25 °C of P(BPin-PA/PO) (top) and zoom in the 1900–400 cm^{-1} region (bottom).

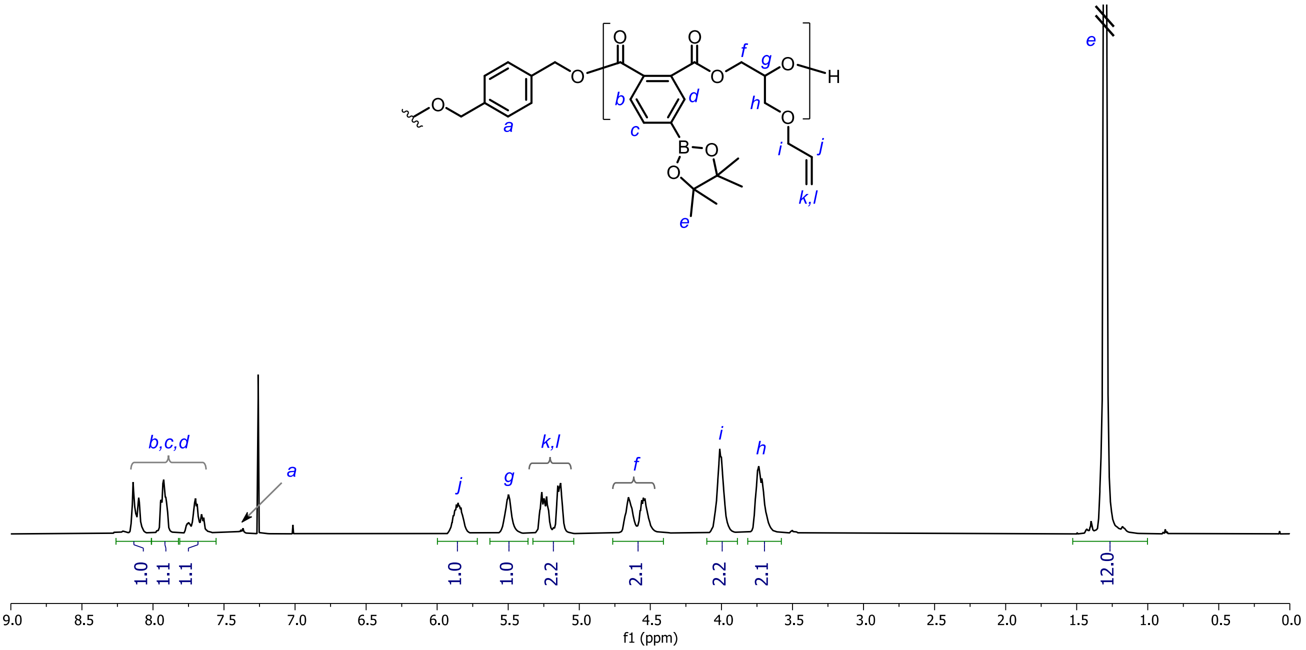

Figure S25. ^{1}H NMR ($CDCl_3$, 25 °C, 499.9 MHz) spectrum of isolated P(BPin-PA/AGE).

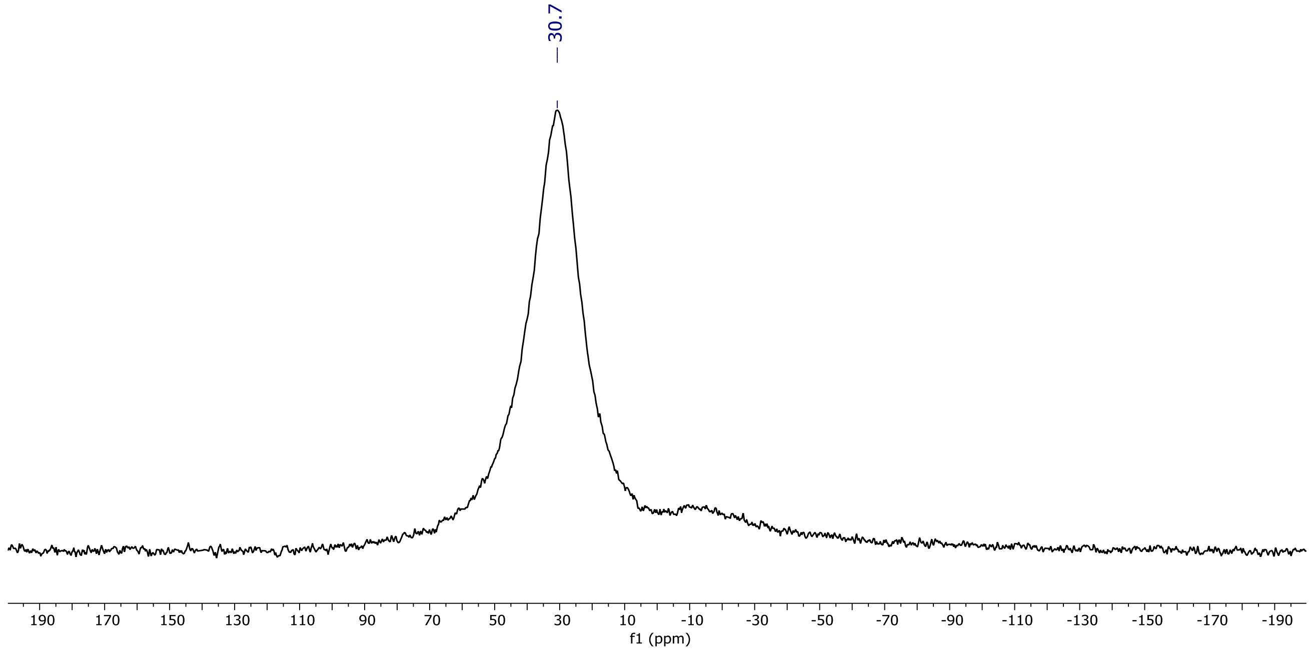

Figure S26. $^{11}B\{^1H\}$ ($CDCl_3$, 25 °C, 160.4 MHz) spectrum of isolated P(BPin-PA/AGE).

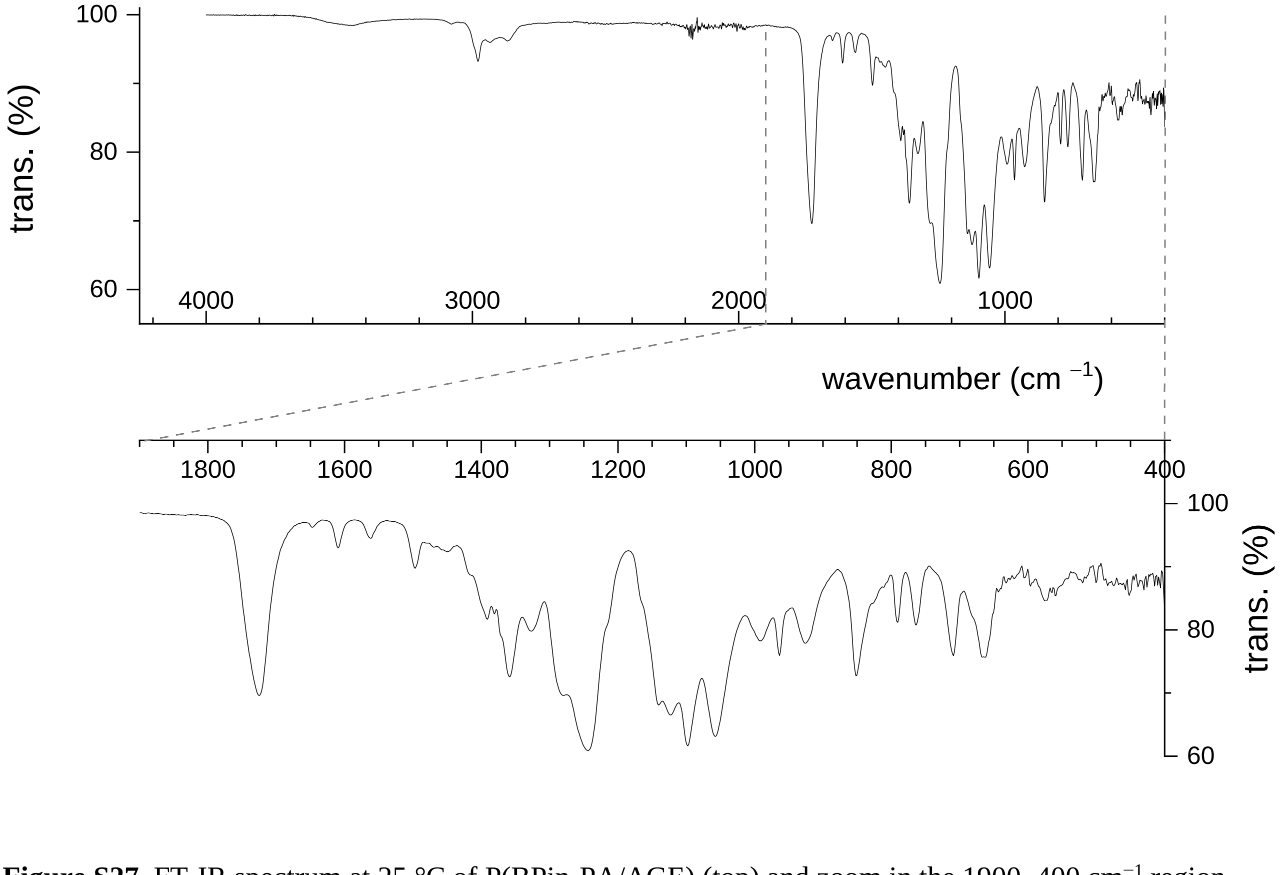

Figure S27. FT-IR spectrum at 25 °C of P(BPin-PA/AGE) (top) and zoom in the 1900–400 cm^{-1} region (bottom).

3.4. Gel Permeation Chromatography (GPC) of BPin-PA Polyesters

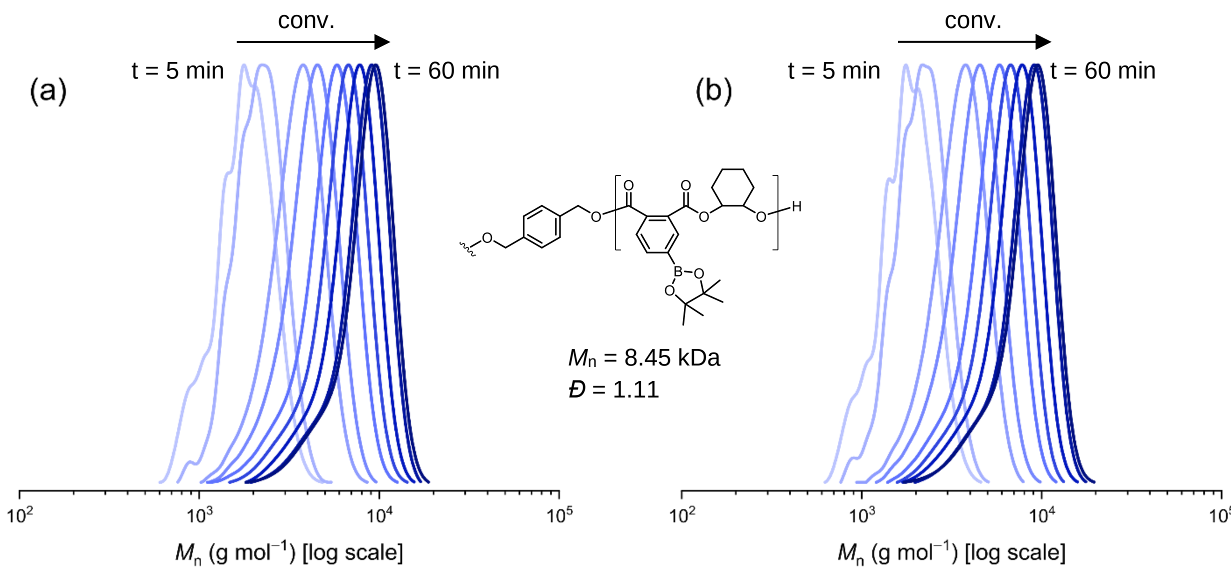

Figure S28. Normalized GPC traces of reaction aliquots during the polymerization of BPin-PA and CHO, catalysed by [ZnMg], displaying the growth in M_n of P[BPin/CHO]: RI detector (a) and UV detector (b) in THF.

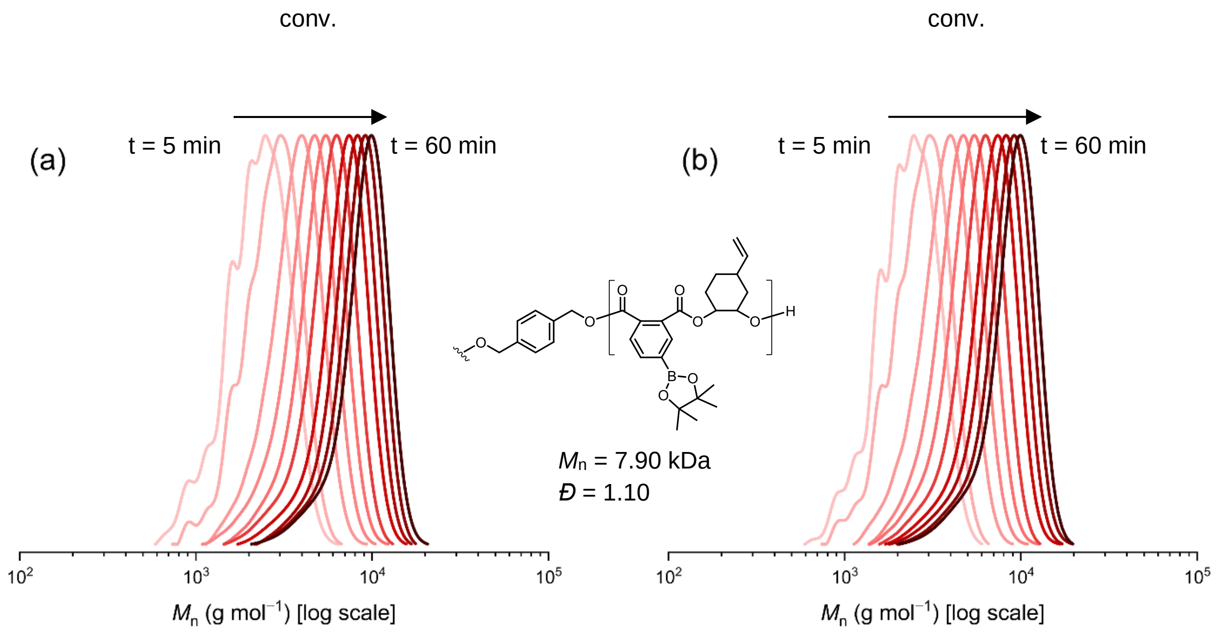

Figure S29. Normalized GPC traces of reaction aliquots during the polymerization of BPin-PA and vCHO, catalysed by [ZnMg], displaying the growth in M_n of P[BPin/vCHO]: RI detector (a) and UV detector (b) in THF.

3.5. MALDI-TOF of P(BPin-PA/vCHO)

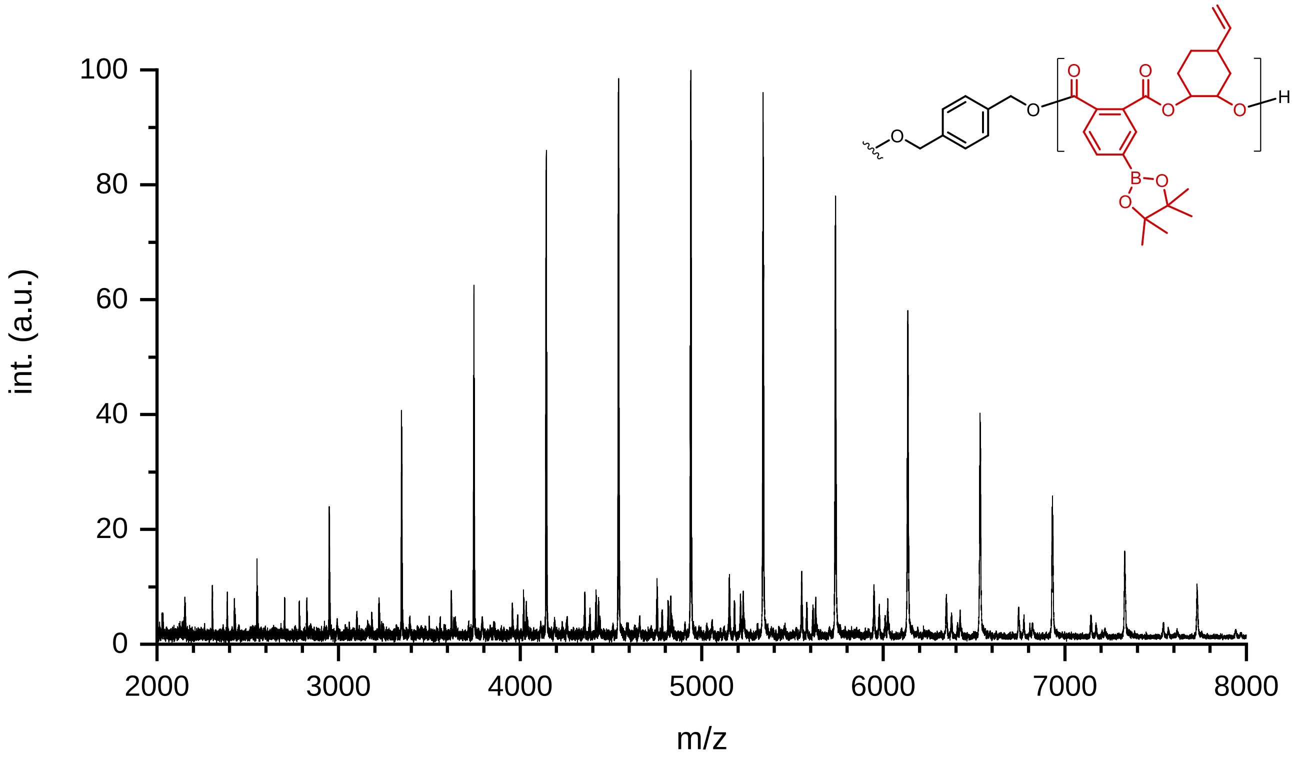

Figure S30. MALDI-TOF spectrum of pinacol ester-protected polymer P(BPin-PA/vCHO), produced by **[ZnMg]** ([BPin-PA]/[vCHO]/[BDM]/[cat] = 50:100:4:1, 80◦C, 2 h. in toluene.

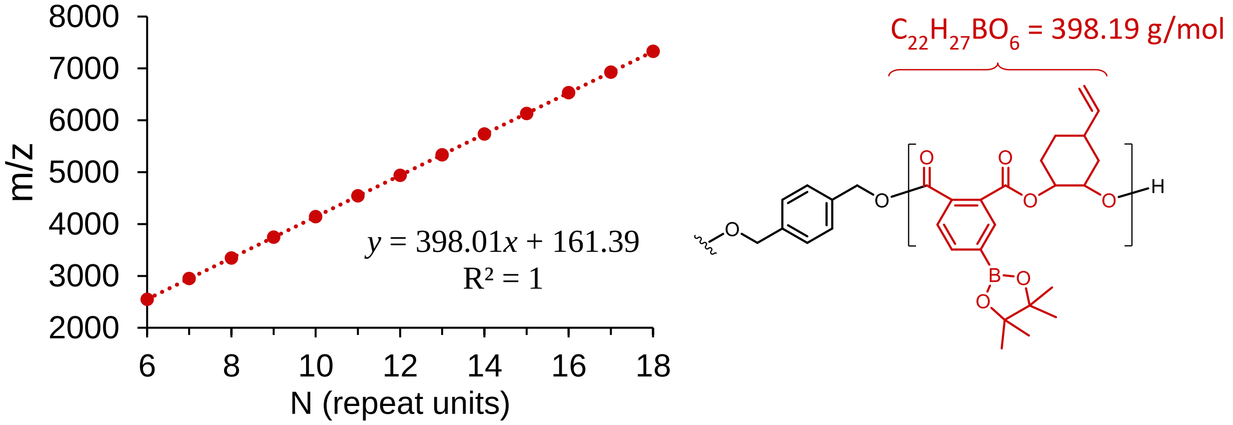

Figure S31. Plot of *m/z* values vs the number of BPin-PA/vCHO repeat units (*n*) from the MALDI-TOF spectrum in Figure S30. Mass of expected end-group ($C_8H_{10}O_2Na$) = 161.06

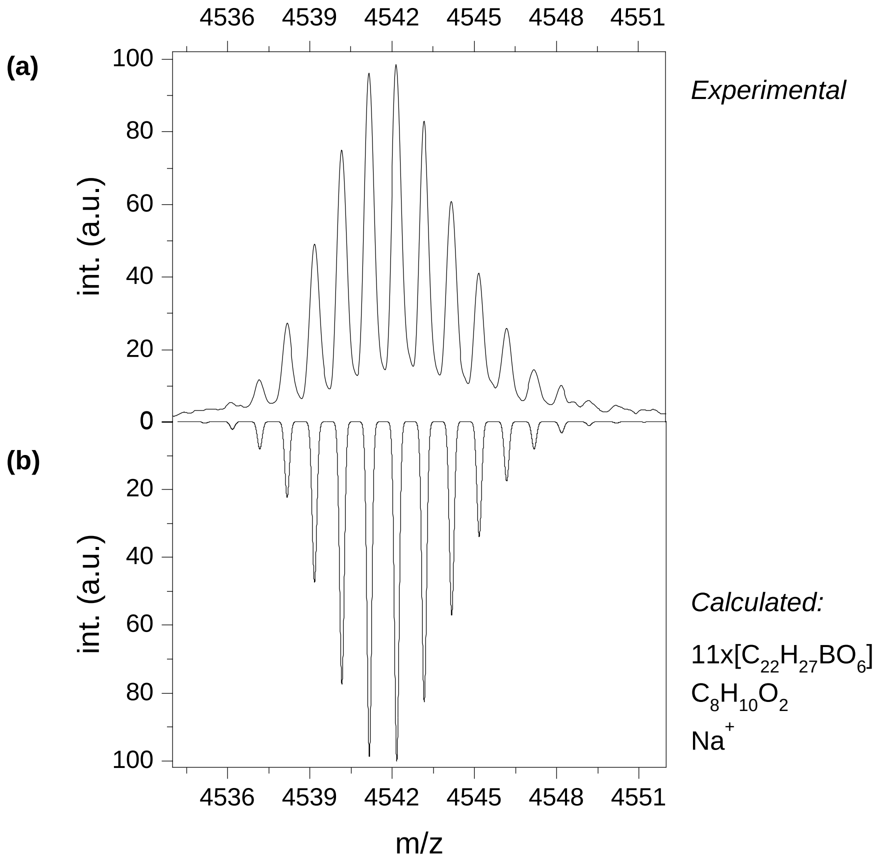

Figure S32. (a) Section of the MALDI-TOF spectrum shown in Figure S30 displaying the isotope distribution pattern, and (b) the calculated isotope distrubtions for the molecular formula $C_{250}H_{307}B_{11}O_{68}Na$, which is consistent with the 11th-mer repeat unit of the pinacol ester-protected polymer P(Pin/vCHO) (featuring BDM/proton end-groups and sodium salt).

3.6. Summary of Thermal Properties of Semi-Aromatic Polyesters

Table S2. Thermal characterization of boron containing polyesters and comparison with poly(phthalic anhydride-alt-epoxide) analogues.

entry #	Polyester	M_n [a] (kDa)	Đ [a]	T_g [b] (°C)	T_d [c] (°C)	*R*ef
1	P(BPin-PA/CHO)	8.5	1.22	213	322	*this work*
2	P(BPin-PA/CHO)	10.6	1.40	224	285	*this work*
3	P(PA/CHO)	17.0	1.10	135	—	[4]
4	P(PA/CHO)	13.2	1.36	146	—	[5]
5	P(BPin-PA/vCHO)	11.8	1.08	199	301	*this work*
6	P(BPin-PA/vCHO)	41.5	1.16	212	293	*this work*
7	P(PA/vCHO)	26.1	1.05	119	—	[4]
8	P(PA/vCHO)	22.1	1.51	128	—	[5]
9	P(BPin-PA/PO)	21.1	1.04	139	300	*this work*
10	P(PA/PO)	16.4	1.04	55	—	[4]
11	P(BPin-PA/AGE)	16.5	1.08	81	294	*this work*
12	P(PA/AGE)	26.9–49.2	1.05–1.06	12–16	—	[4]
13	P(PA/AGE)	11.2	1.27	−1	—	[5]
14	P[B(OH)$_2$-PA/CHO]	7.28	1.09	n.d.	266	*this work*
15	P[B(OH)$_2$-PA/vCHO]	12.1	1.15	n.d.	270	*this work*
16	P[B(OH)$_2$-PA/PO]	18.2	1.09	n.d.	314	*this work*
17	P[B(OH)$_2$-PA/AGE]	14.8	1.08	n.d.	306	*this work*

[a] Determined by gel permeation chromatography (THF) against polystyrene standards; P[B(OH)$_2$-PA/epoxide] polyester solutions in THF/H_2O containing ~30 mg mL^{-1} of NPG. [b] Obtained by differential scanning calorimetry during the second heating scan (10 °C min^{-1}). [b] Obtained by thermogravimetric analysis (10 °C min^{-1}). n.d. = not detected.

3.7. Differential Scanning Calorimetry (DSC) of BPin-Polyesters

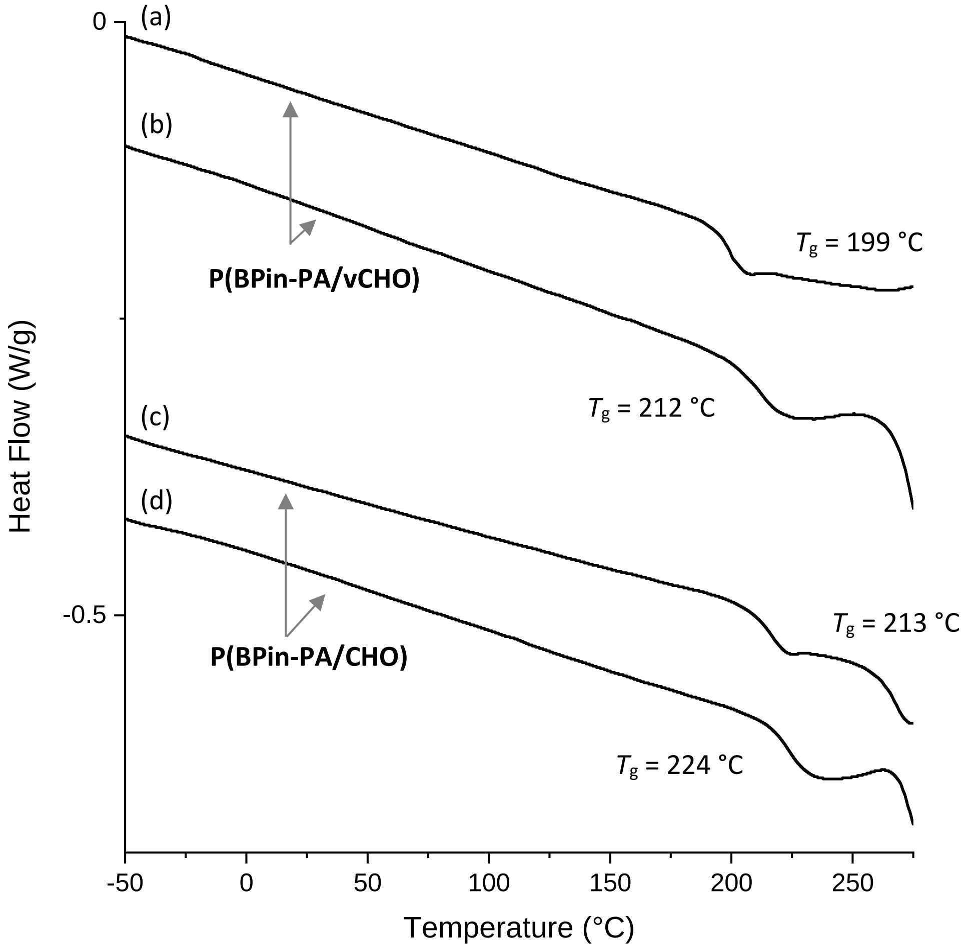

Figure S33. DSC thermograms during the second heating scan, recorded at 10 °C min^{-1}, for P(BPin-PA/vCHO) and P(BPin-PA/CHO). Samples are listed in Table S2 entries 5 (a), 6 (b), 1 (c), and 2 (d). Degradation onsets are observed at ~275 °C.

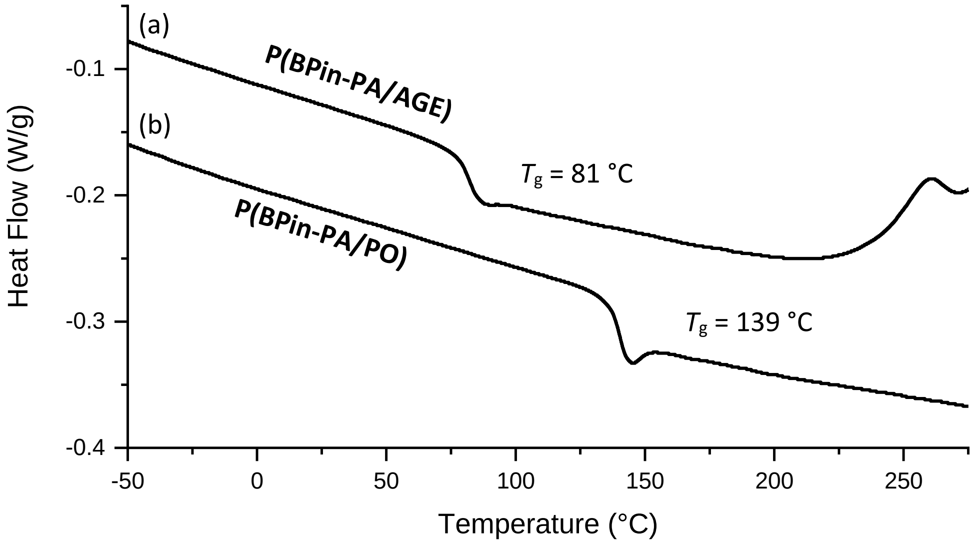

Figure S34. DSC thermograms during the second heating scan, recorded at 10 °C min^{-1}, for P(BPin-PA/AGE) and P(BPin-PA/PO). Samples are those listed in Table S2 entries 11 (a), and 9 (b).

3.8. Thermogravimetric Analysis (TGA) of BPin-Polyesters

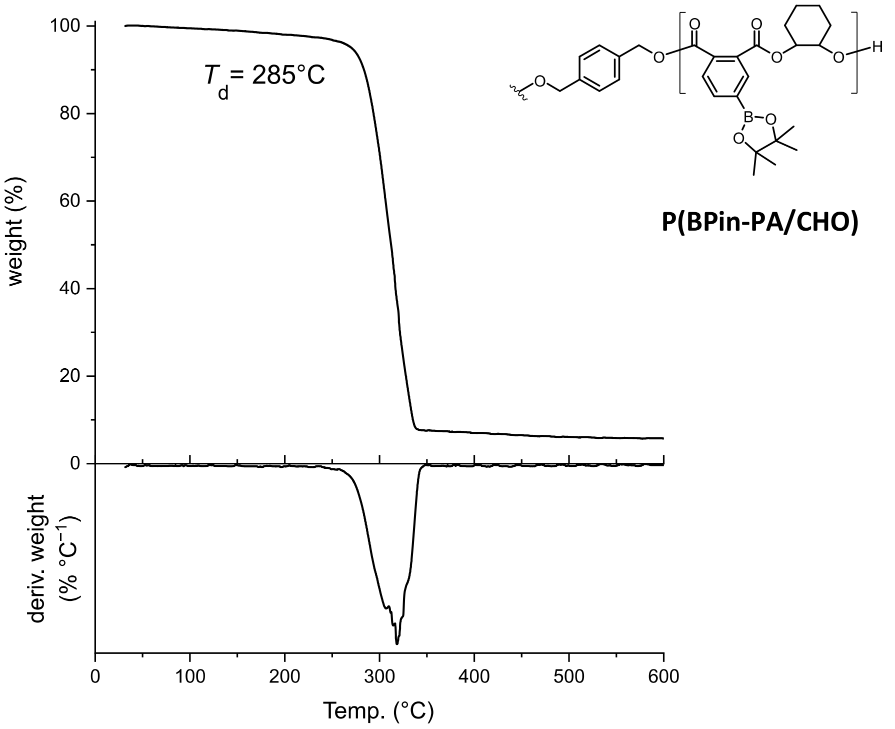

Figure S35. TGA Data for P(BPin-PA/CHO) recorded at 10 °C min^{-1} (top), and the first derivative (bottom).

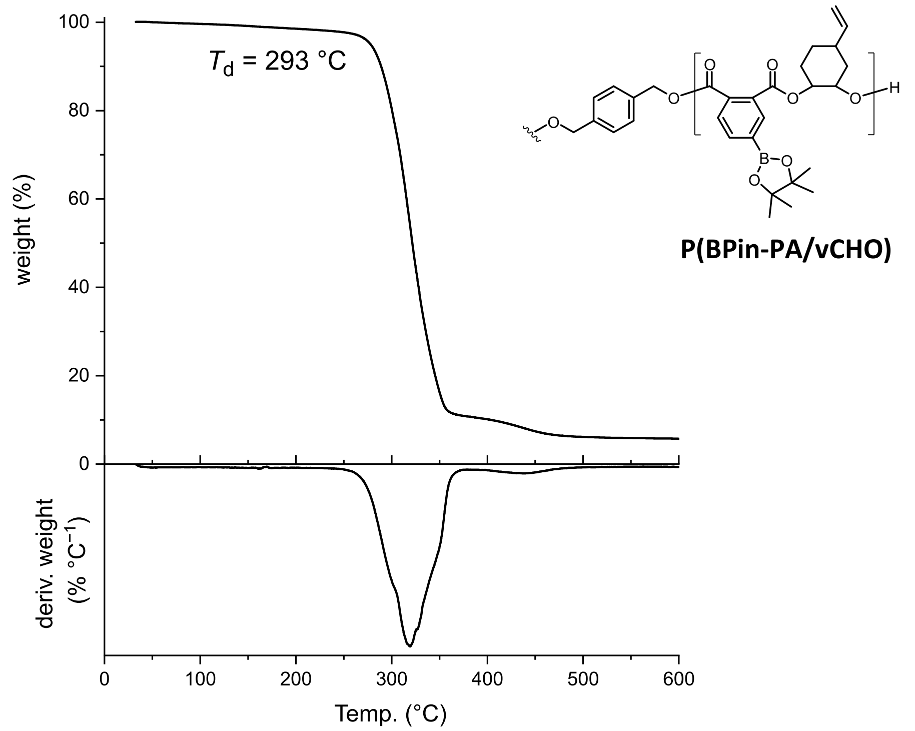

Figure S36. TGA Data for P(BPin-PA/vCHO) recorded at 10 °C min^{-1} (top), and the first derivative (bottom).

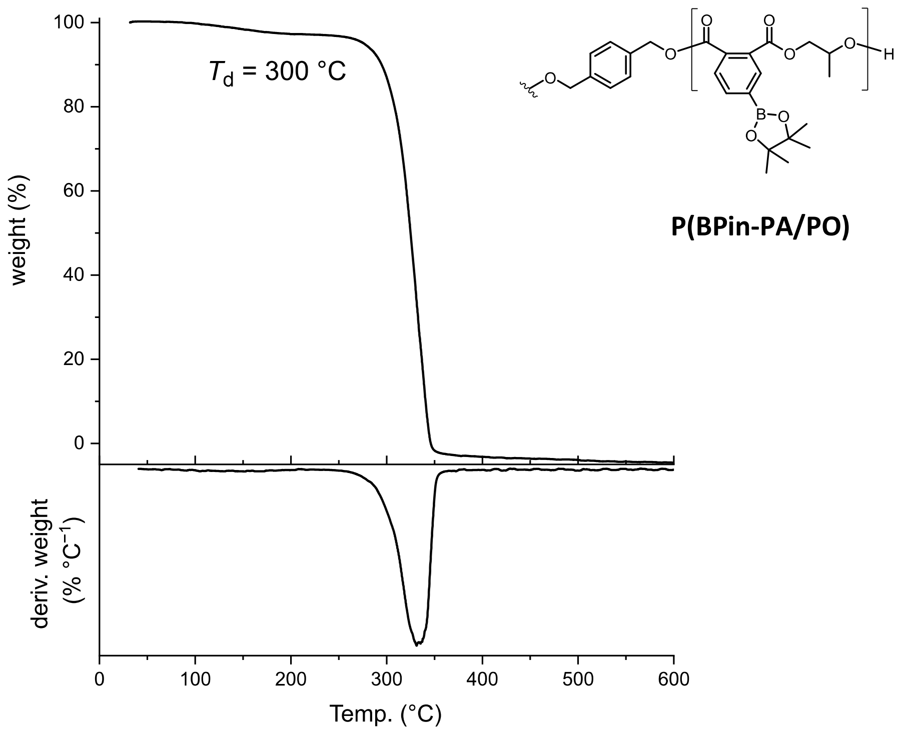

Figure S37. TGA Data for P(BPin-PA/PO) recorded at 10 °C min^{-1} (top), and the first derivative (bottom).

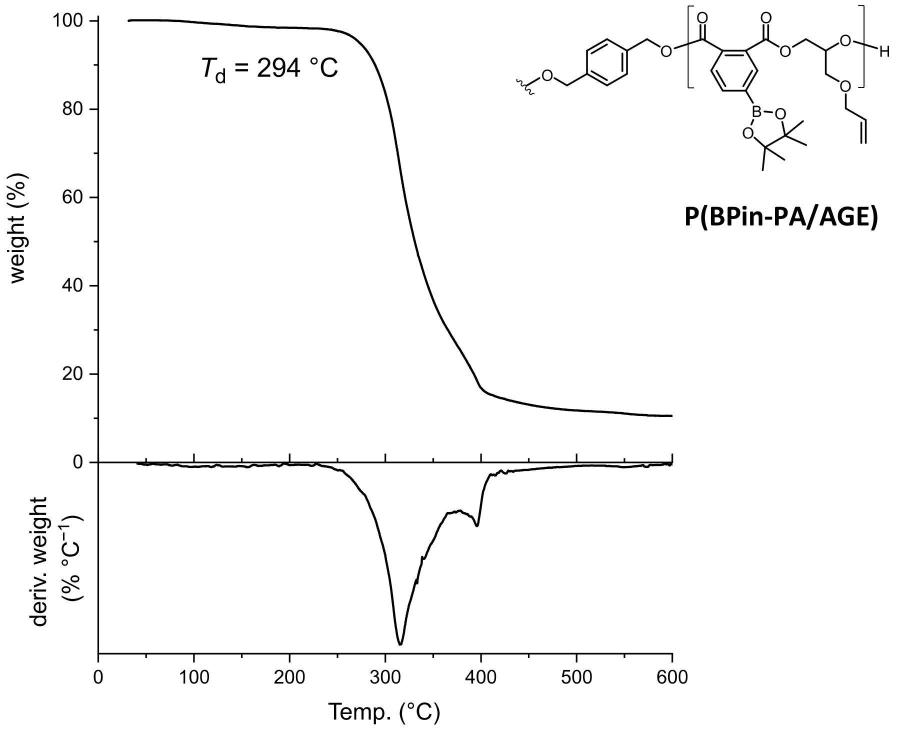

Figure S38. TGA Data for P(BPin-PA/AGE) recorded at 10 °C min^{-1} (top), and the first derivative (bottom).

4. Deprotection to Boronic Acid-Polymers

4.1. Investigation of boronic ester transesterification using small-molecule model

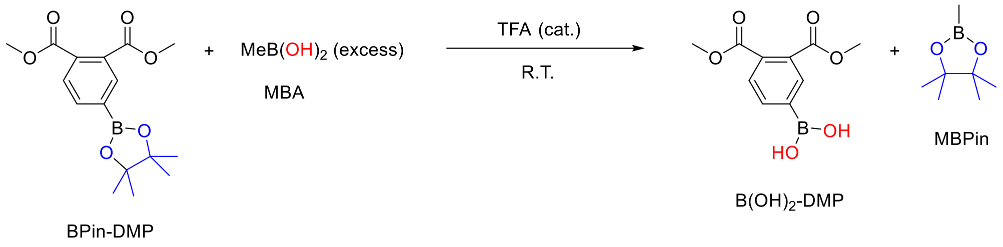

In air, a 40 mL reaction vial, containing a magnetic stir bar, was loaded with dimethyl phthalate pinacolboronate ester, BPin-DMP, (160 mg, 0.5 mmol) and methylboronic acid, MBA, (5.0 or 20.0 mmol). The solids were dissolved in a solution of trifluoroacetic acid (2% or 5%) in the desired solvent, and stirred at room temperature for the appropriate time interval. At this point, aliquots were taken, diluted in acetone-d_6 and analysed by ^{1}H NMR spectroscopy to obtain conversion data.

Table S3. Optimization of transesterification conditions, at room temperature.

entry #	Solvent	TFA (%)	$MeB(OH)_2$ eq.	$[(BPin)DMP]_0$	Time (h)	Conv. (%)^a
1	DCM	5	5	0.1	22	88
2	DCM/Acet. 1:1	5	20	0.1	3	96
3	DCM/Acet. 1:1	2	20	0.1	3	94
4	THF	2	20	0.06	22	95
5	THF	2	20	0.03	22	89

^{a} Determined from the ^{1}H NMR spectra of reaction aliquots.

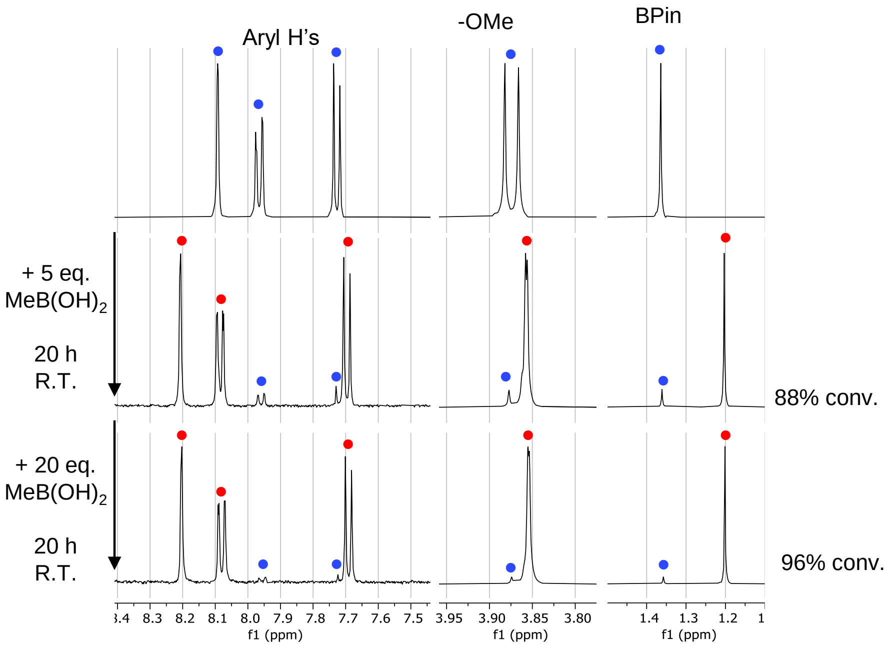

Figure S39. Sequential ^{1}H NMR spectra (acetone-d_6) displaying conversion of BPin-DMP in DCM (top); after equilibration of the transesterification reaction with 5 equiv. (middle) and 25 equiv. (total) of $MeB(OH)_2$ (bottom). Blue labelled signals: BPin-DMP; red labelled signals: $B(OH)_2$-DMP.

4.2. General method for the deprotection of boronic-ester polyesters

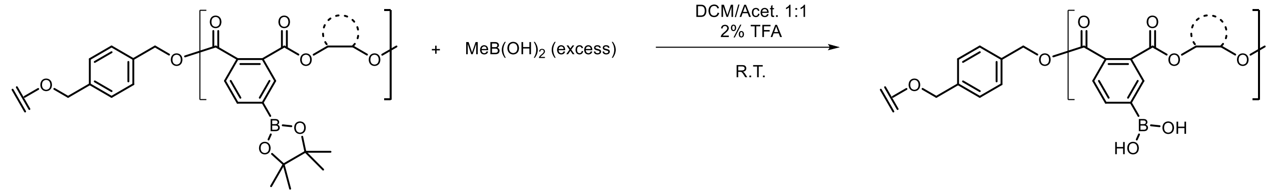

In a 50 mL round-bottom flask, the boronic ester-polymer (200 mg, 0.5–0.8 mmol in boron units) and methylboronic acid (599–958 mg, 10–16 mmol, 20 equiv.) were loaded and dissolved in a mixture of acetone/CH_2Cl_2 2:1 (v:v) so that the final concentration was 0.15 M. To this solution was added trifluoroacetic acid (2%) and the resulting homogeneous transparent solution was stirred, at room temperature, for 20 h. Then, the volatiles were removed, under vacuum at 40 °C, in the rotavapor for ~1 h. The resulting residue was redissolved in acetone (2 mL), and the solution evaporated, at 60 °C in the rotavapor, for 2 hours. The resulting powder was redissolved in acetone (2 mL) and precipitated thrice over a large excess of distilled H_2O (3 × 100 mL); the polymer was separated by centrifugation, and dried under vacuum, at 60 °C until constant weight (yields varied between ca. 50-70% due to losses during manipulation).

P[B(OH)$_2$-PA/CHO]. ^{1}H NMR [$(CD_3)_2CO$, 25 ◦C, 499.9 MHz]: *δ* 8.21 (bs, 1H, Ar-*H*); 8.02 (bs, 1H, Ar-*H*); 7.65 (bs, 1H, Ar-*H*); 7.47 (s, Ar-*H* 1,4-BDM end-group); 5.10 (bs, 2H, –OC*H*–); 3.37 (bs, 2H, B–O*H*); 2.14 (bs, 2H, –C*H*$_2$–); 1.70 (bs, 2H, –C*H*$_2$–); 1.57 (bs, 2H, –C*H*$_2$–); 1.39 (bs, 2H, –C*H*$_2$–). ^{11}B{^{1}H} NMR [$(CD_3)_2CO$, 25 ◦C, 160.4 MHz]: *δ* 27.5. FT-IR (25 °C, cm^{-1}): 3470, 2940, 2866, 1721, 1607, 1561, 1495, 1321, 1254, 1155, 1122, 1062, 1024, 989, 918, 845, 793, 769, 711, 662.

P[B(OH)$_2$-PA/vCHO]. ^{1}H NMR [$(CD_3)_2CO$, 25 ◦C, 499.9 MHz]: *δ* 8.24 (bs, 1H, Ar-*H*); 8.06 (bs, 1H, Ar-*H*); 7.75 (bs, 1H, Ar-*H*); 7.49 (s, Ar-*H* 1,4-BDM end-group); 5.81 (bs, 1H, –C*H*=C); 5.36–4.96 (m, 4H, –OC*H*– and C=C*H*$_2$); 3.57 (bs, 2H, B–O*H*); 2.48 (bs, 1H, –C*H*–); 1.98 (bs, 4H, –C*H*$_2$–); 1.68–1.57 (bs, 2H, –C*H*$_2$–). ^{11}B{^{1}H} NMR [$(CD_3)_2CO$, 25 ◦C, 160.4 MHz]: *δ* 28.8. FT-IR (25 °C, cm^{-1}): 3475, 3073, 2935, 2859, 1720, 1640, 1605, 1561, 1495, 1245, 1117, 1061, 1117, 1061, 992, 966, 915, 871, 848, 791, 771, 712, 661.

P[B(OH)$_2$-PA/PO]. ^{1}H NMR [$(CD_3)_2CO$, 25 ◦C, 499.9 MHz]: *δ* 8.25 (bs, 1H, Ar-*H*); 8.06 (bs, 1H, Ar-*H*); 7.80–7.69 (bs, 1H, Ar-*H*); 7.44 (s, Ar-*H* 1,4-BDM end-group); 5.41 (bs, 1H, –OC*H*–); 4.44 (bs, 2H, –C*H*$_2$–); 3.24 (bs, 2H, B–O*H*); 1.38–1.35 (bs, 3H, –C*H*$_3$). ^{11}B{^{1}H} NMR [$(CD_3)_2CO$, 25 ◦C, 160.4 MHz]: *δ* 28.3.

P[B(OH)$_2$-PA/AGE]. ^{1}H NMR [$(CD_3)_2CO$, 25 ◦C, 499.9 MHz): *δ* 8.26 (bs, 1H, Ar-*H*); 8.08 (bs, 1H, Ar-*H*); 7.76 (bs, 1H, Ar-*H*); 7.44 (s, Ar-*H* 1,4-BDM end-group); 5.87 (bs, 1H, –C*H*=C); 5.52 (bs, 1H, –OC*H*–); 5.25 (d, *J* = 17.5 Hz, 1H, C=C*H*$_2$); 5.10 (d, *J* = 10.5 Hz, 1H, C=C*H*$_2$); 4.65–4.57 (bs, 2H, O–C*H*$_2$); 4.02 (bs, 2H, O–C*H*$_2$); 3.77 (bs, 2H, O–C*H*$_2$); 3.36 (bs, 2H, B–O*H*). ^{11}B{^{1}H} NMR [$(CD_3)_2CO$, 25 ◦C, 160.4 MHz): *δ* 28.6.

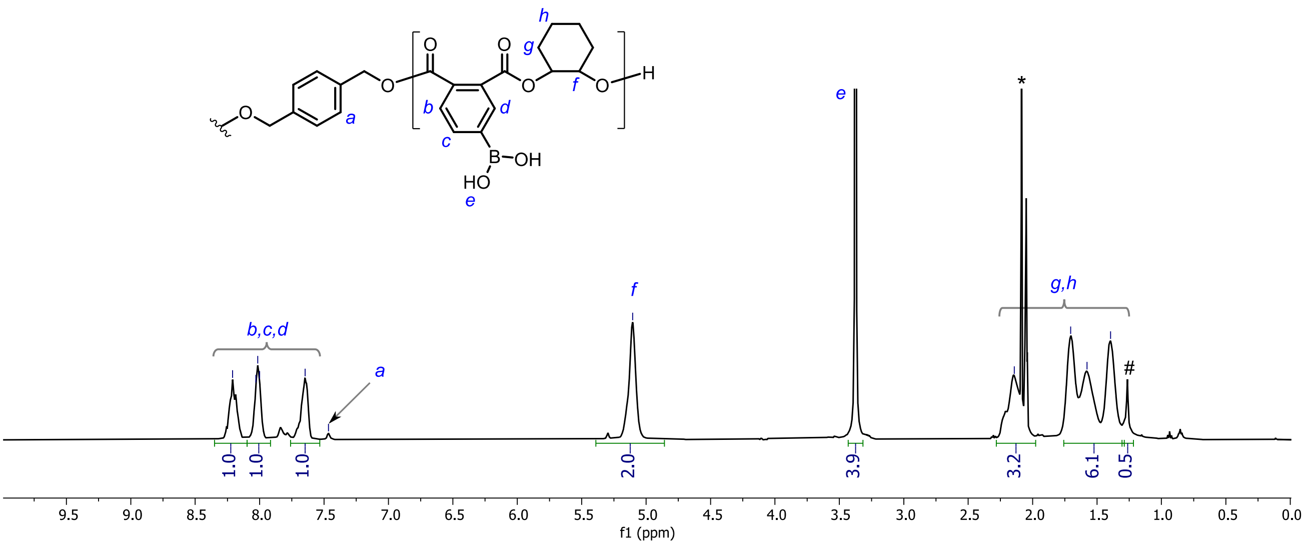

Figure S40. ^{1}H NMR [$(CD_3)_2CO$, 25 °C, 499.9 MHz] spectrum of isolated P[$B(OH)_2$-PA/CHO]. (* = residual acetone; # = unreacted –BPin, <5%).

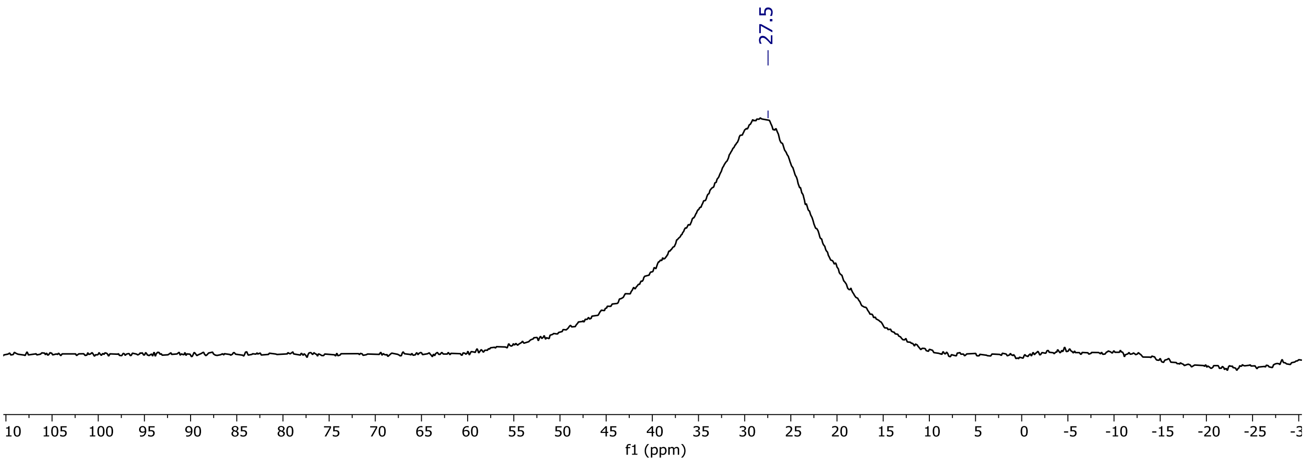

Figure S41. $^{11}B\{^{1}H\}$ NMR [$(CD_3)_2CO$, 25 °C, 160.4 MHz) spectrum of isolated P[$B(OH)_2$-PA/CHO].

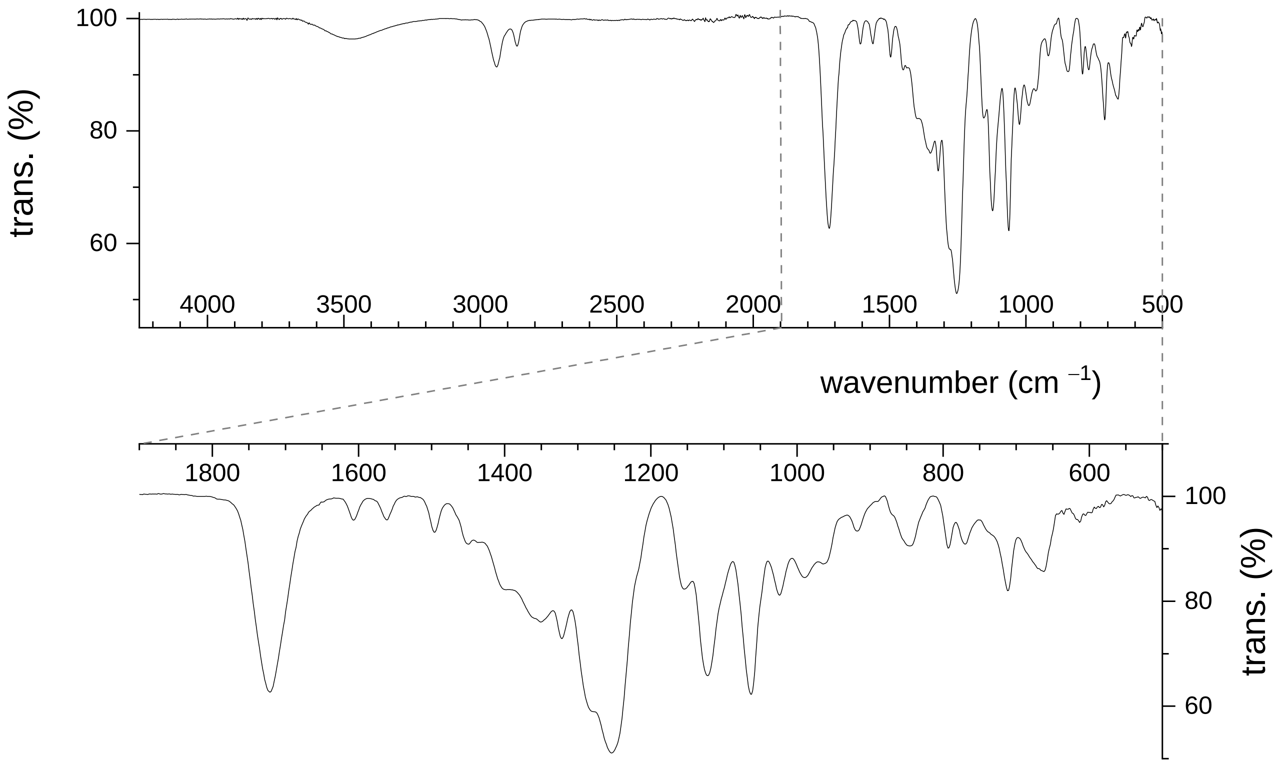

Figure S42. FT-IR spectrum at 25 °C of $P(B(OH)_2/CHO)$ (top) and zoom in the 1900–500 cm^{-1} region (bottom).

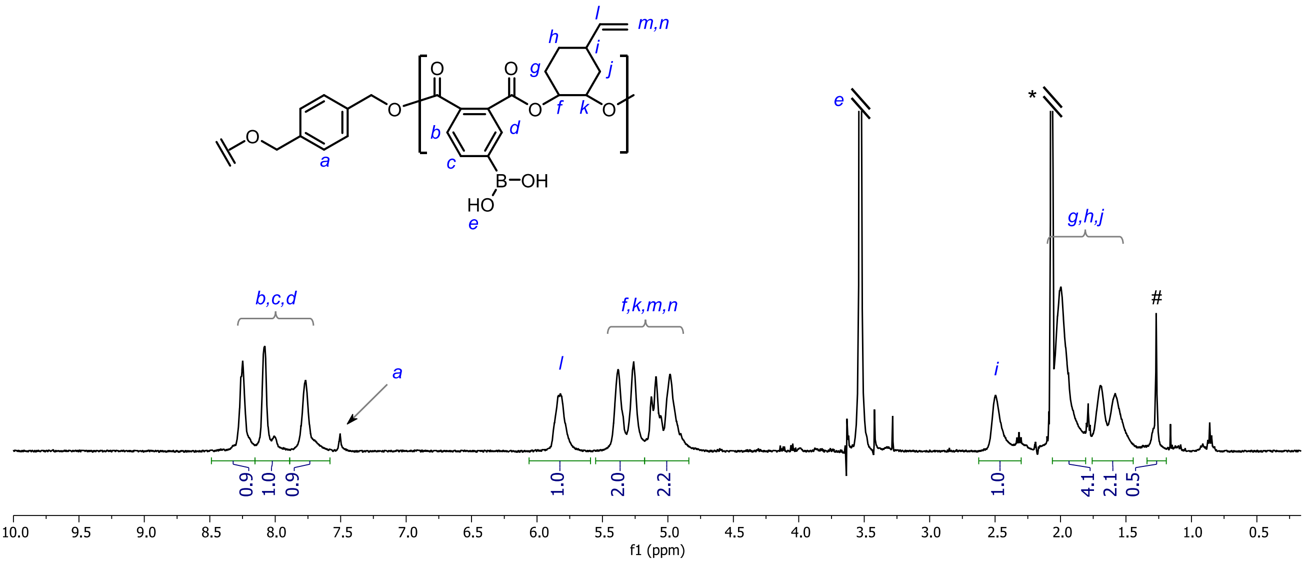

Figure S43. ^{1}H NMR [$(CD_3)_2CO$, 25 °C, 499.9 MHz] spectrum of isolated $P[B(OH)_2$-PA/vCHO]. [* = $(CD_3)_2CO$; # = unreacted –BPin, <5%].

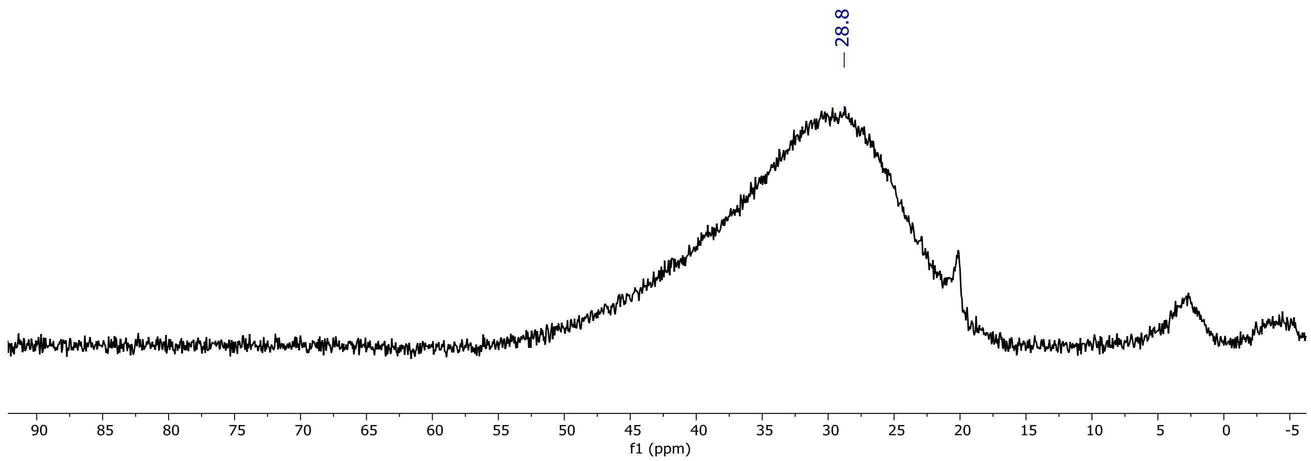

Figure S44. $^{11}B\{^{1}H\}$ NMR [$(CD_3)_2CO$, 25 °C, 160.4 MHz) spectrum of isolated P[$B(OH)_2$-PA/vCHO].

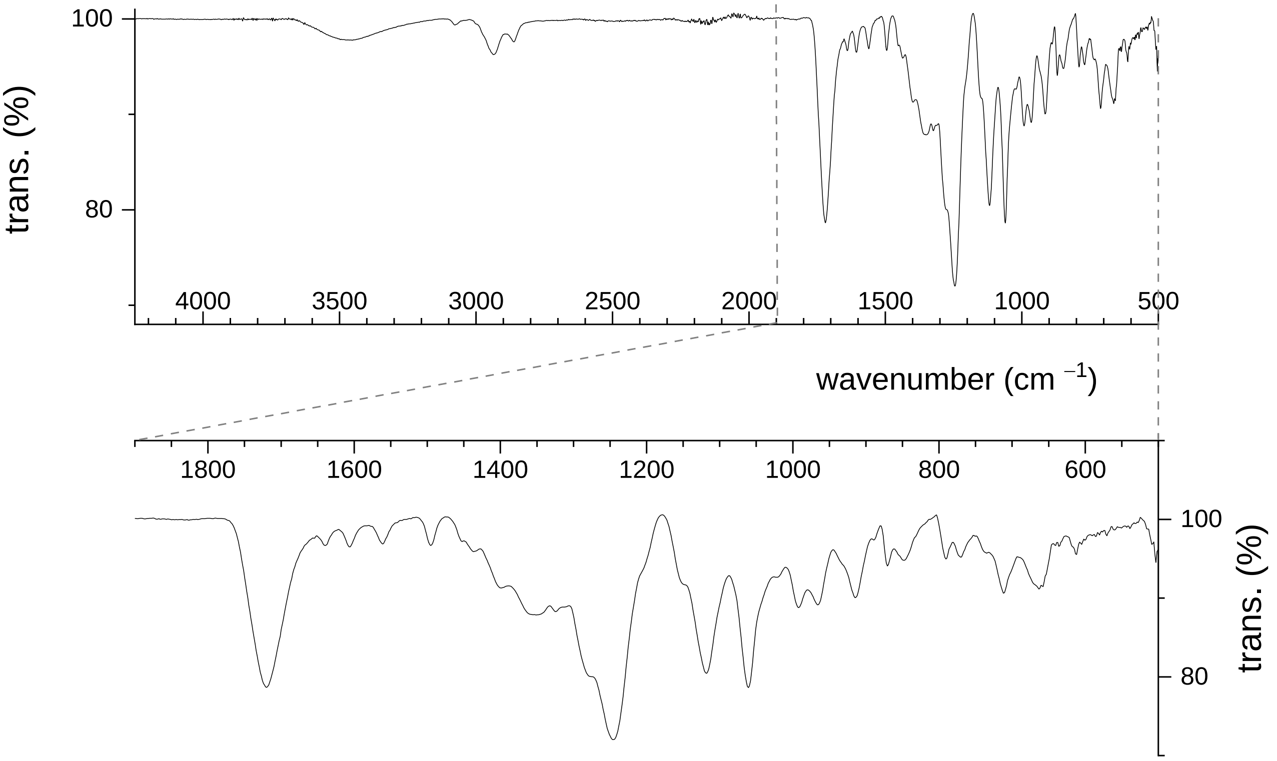

Figure S45. FT-IR spectrum at 25 °C of P($B(OH)_2$/vCHO) (top) and zoom in the 1900–500 cm^{-1} region (bottom).

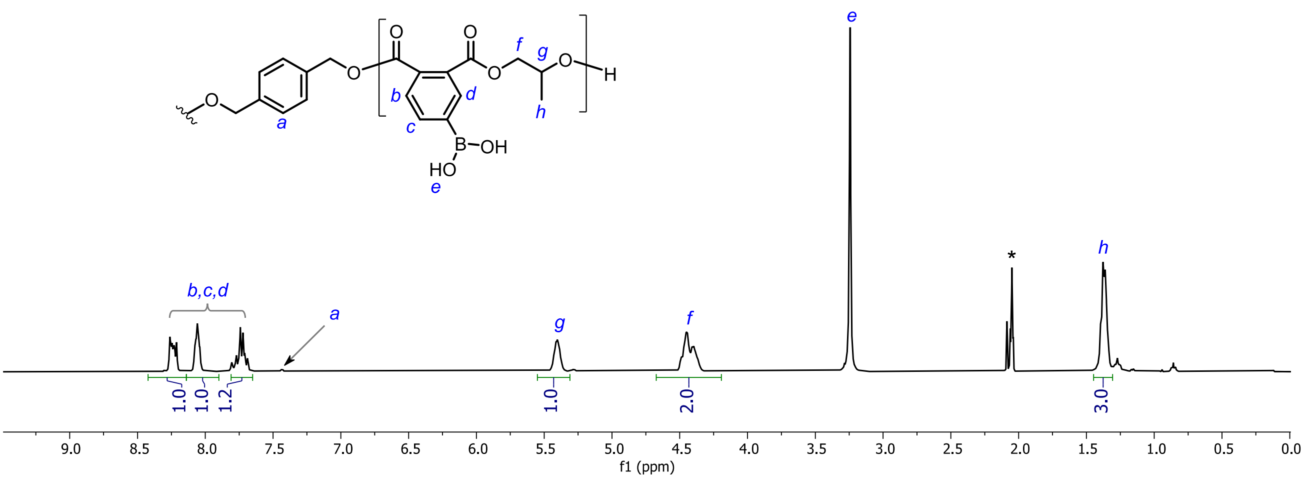

Figure S46. ^{1}H NMR [$(CD_3)_2CO$, 25 °C, 499.9 MHz] spectrum of isolated P[$B(OH)_2$-PA/PO]. [* = $(CD_3)_2CO$].

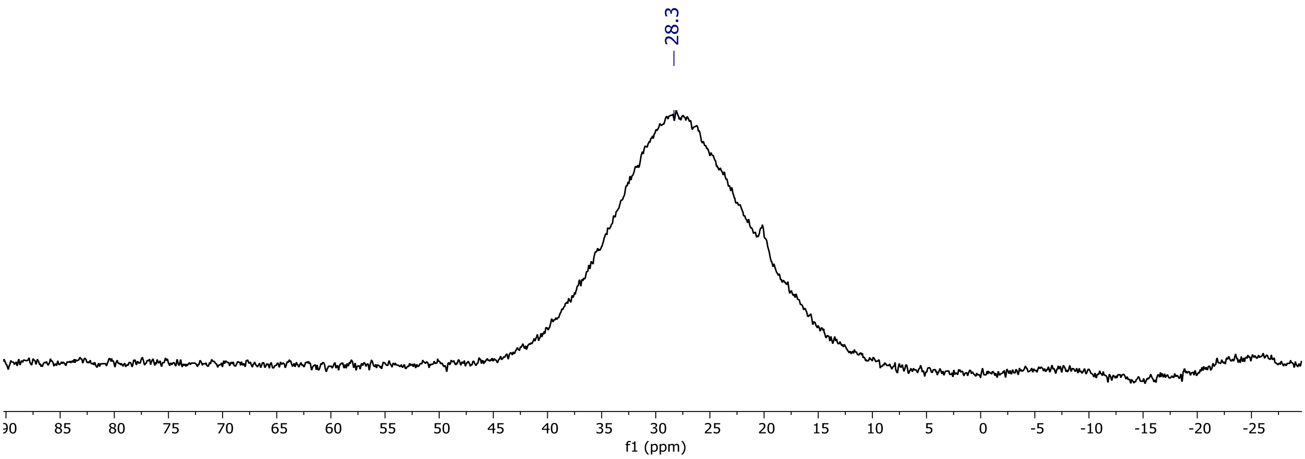

Figure S47. $^{11}B\{^{1}H\}$ NMR [$(CD_3)_2CO$, 25 °C, 160.4 MHz) spectrum of isolated P[$B(OH)_2$-PA/PO].

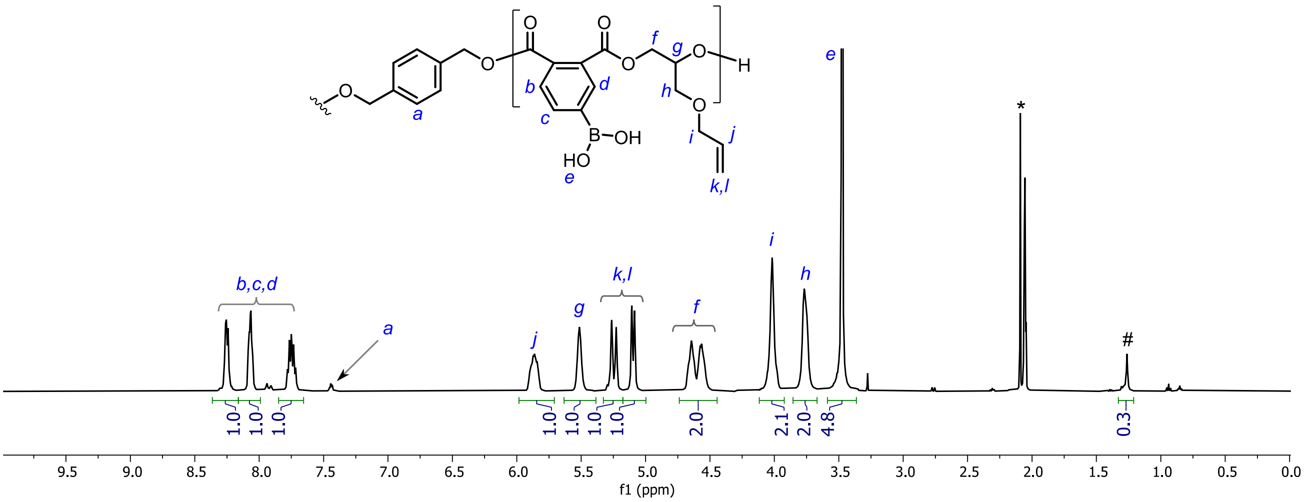

Figure S48. ^{1}H NMR [$(CD_3)_2CO$, 25 °C, 499.9 MHz] spectrum of isolated P[$B(OH)_2$-PA/AGE]. (* = residual acetone; # = unreacted –BPin, <5%).

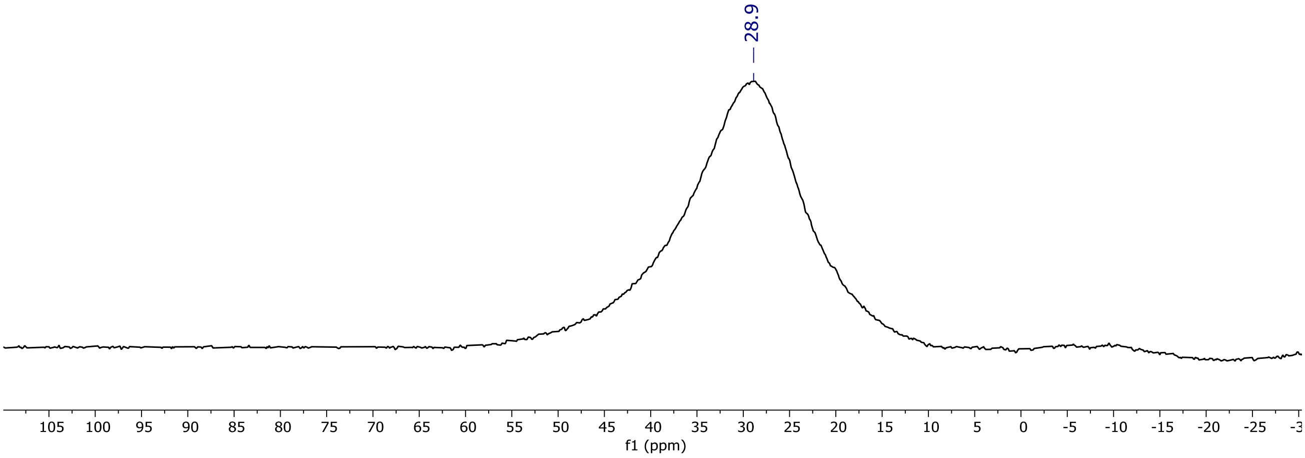

Figure S49. $^{11}B\{^1H\}$ NMR [$(CD_3)_2CO$, 25 °C, 160.4 MHz) spectrum of isolated P[$B(OH)_2$-PA/AGE].

4.3. GPC Analysis of Boronic Acid-Polyesters.

In an Eppendorf tube, dry polymer samples were suspended in THF (1.2 mL of a 5 mg mL^{-1} solution). Then, distilled H_2O (10 µL) was added, and the polymer solutions were vortexed until complete dissolution. Subsequently, neopentyl glycol (NPG) from a stock solution in THF (400 mg mL^{-1}) was added in the desired ratio (5–100 µL). These sample solutions were vortexed for a minute, filtered through 25 µm PTFE filters, and analysed by GPC.

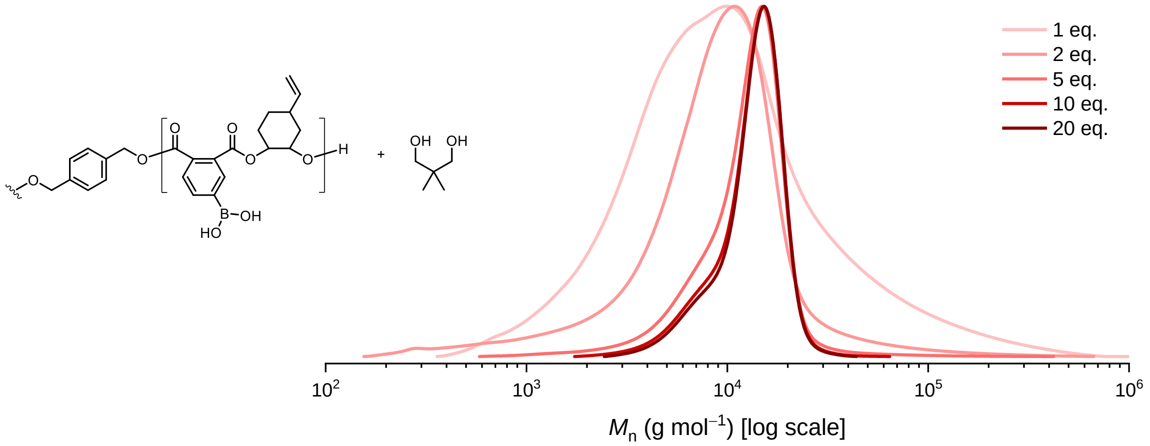

Figure S50. Normalized GPC traces (UV detector) of boronic acid- polyester P[B(OH)$_2$-PA/vCHO] (5 mg/mL in THF) with increasing amounts of NPG.

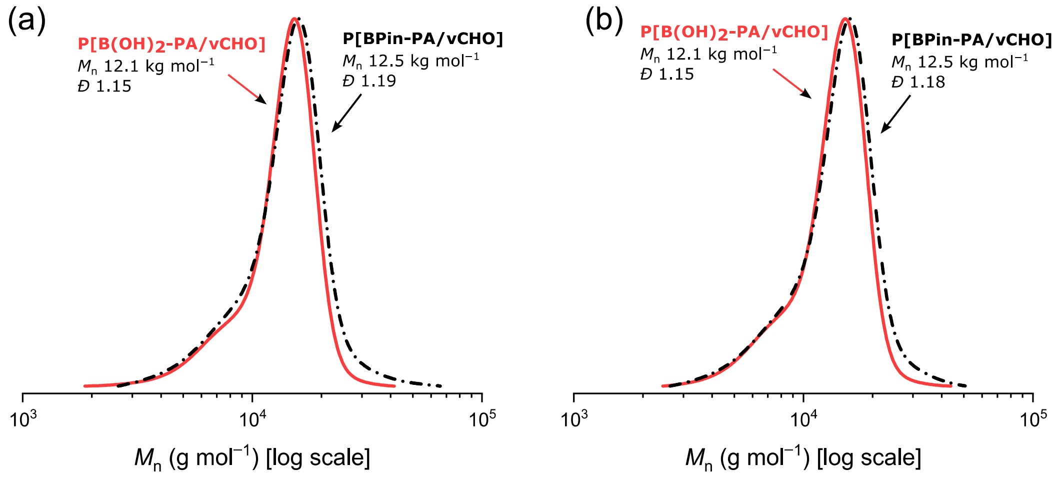

Figure S51. Comparison of GPC traces (normalized) of P[BPin/vCHO] polyester before (dashed black line) and after (red line) the transesterification reaction with MeB(OH)$_2$. (a) RI detector, (b) UV detector in THF. P[B(OH)$_2$-PA/vCHO] was dissolved in THF/H_2O solution containing ~30 mg mL^{-1} of NPG.

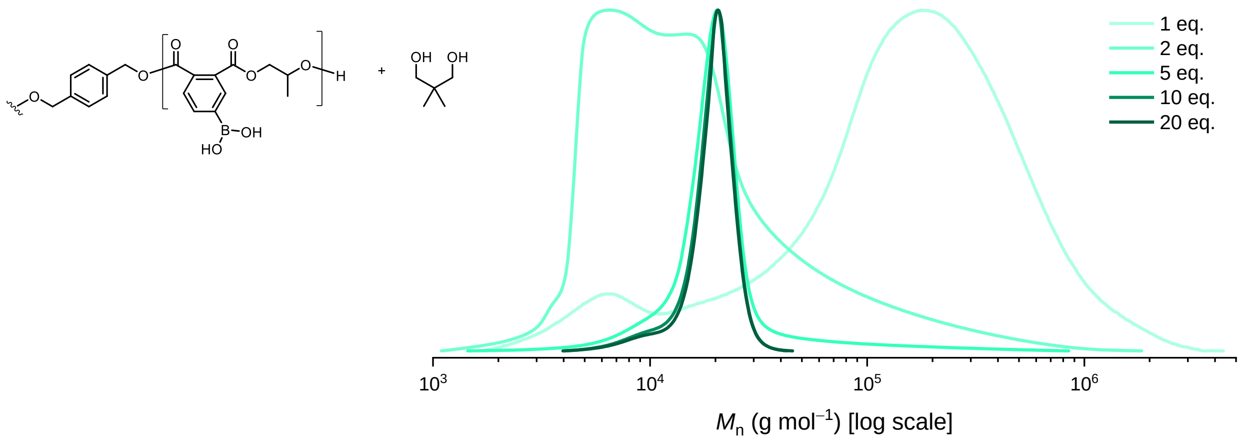

Figure S52. Normalized GPC traces (UV detector) of boronic acid-containing polyester P[B(OH)$_2$-PA/PO] (5 mg/mL in THF) with increasing amounts of NPG.

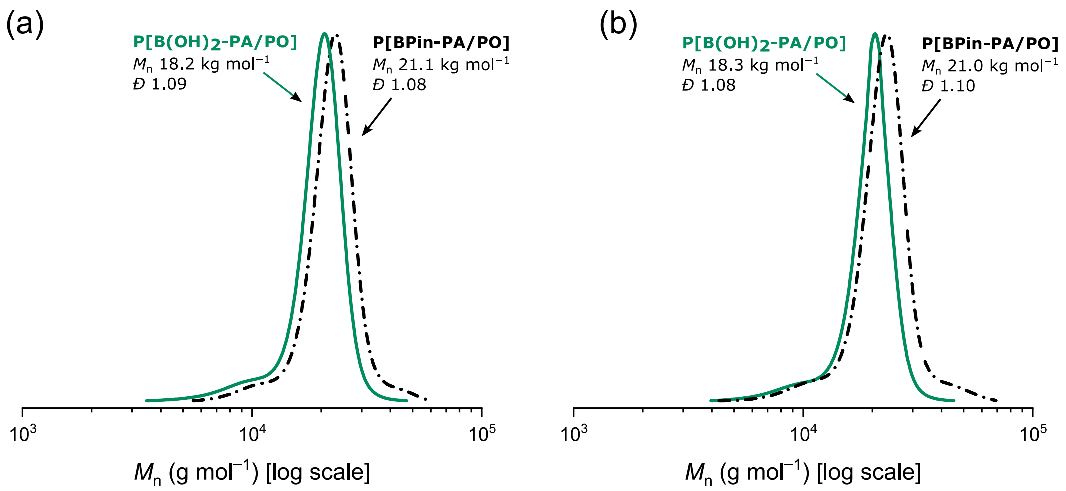

Figure S53. Comparison of GPC traces (normalized) of P[BPin/PO] polyester before (dashed black line) and after (red line) the transesterification reaction with MeB(OH)$_2$. (a) RI detector, (b) UV detector in THF. P[B(OH)$_2$-PA/PO] was dissolved in THF/H_2O solution containing ~30 mg mL^{-1} of NPG.

4.4. MALDI-TOF Spectrum of P[B(NPG)-PA/vCHO]

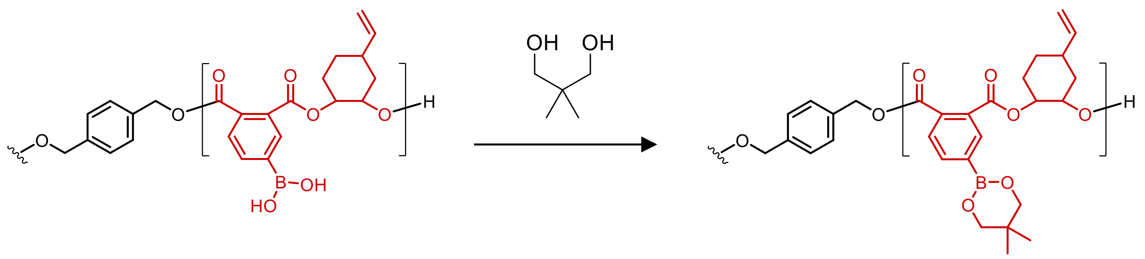

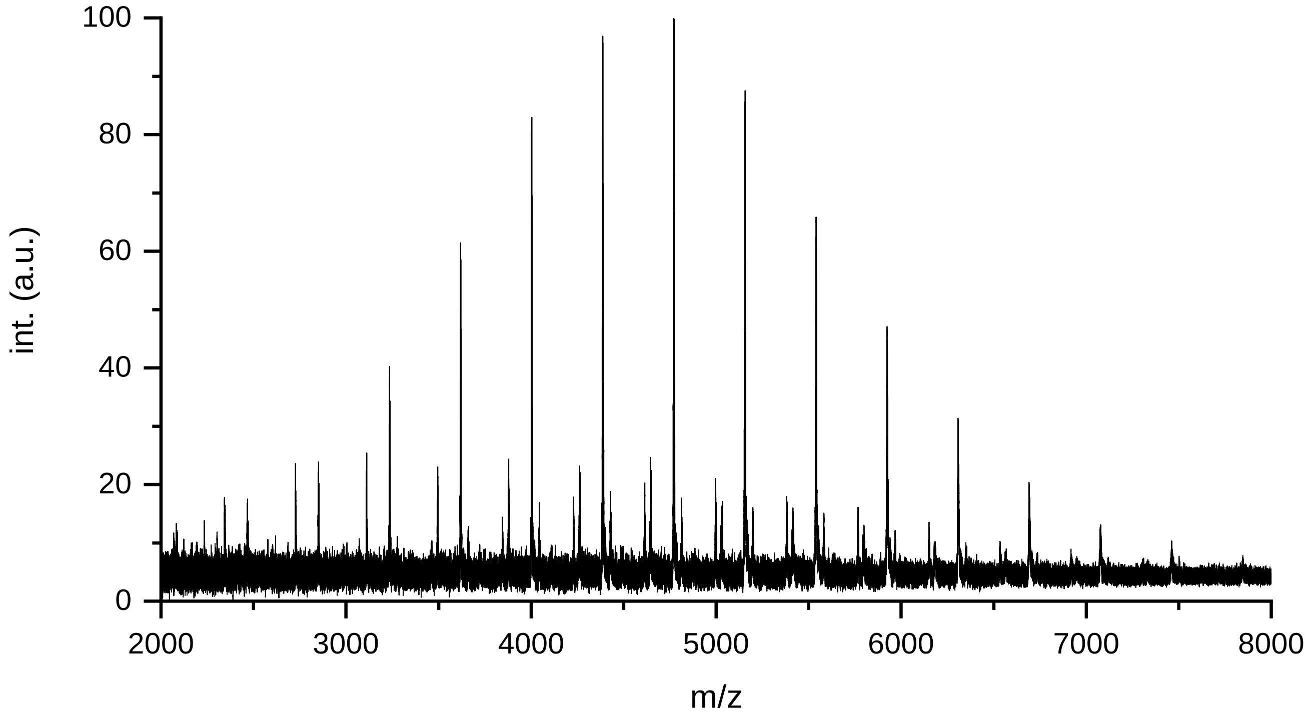

Figure S54. MALDI-TOF spectrum of neopentyl glycol ester-protected polymer P[B(NPG)-PA/vCHO] *in-situ* generated from boronic acid-polymer P[B(OH)$_2$-PA/vCHO] and neopentyl glycol.

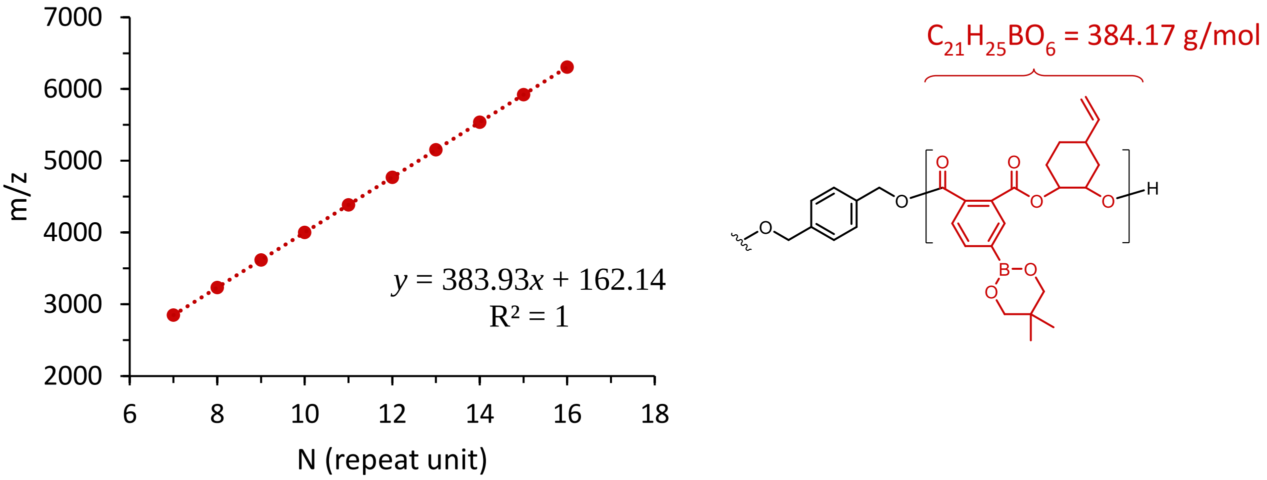

Figure S55. Plot of *m/z* values vs. the number of B(NPG)-PA/vCHO repeat units (*n*) from the MALDI-TOF spectrum in Figure S54. Mass of expected end-group ($C_8H_{10}O_2Na$) = 161.06

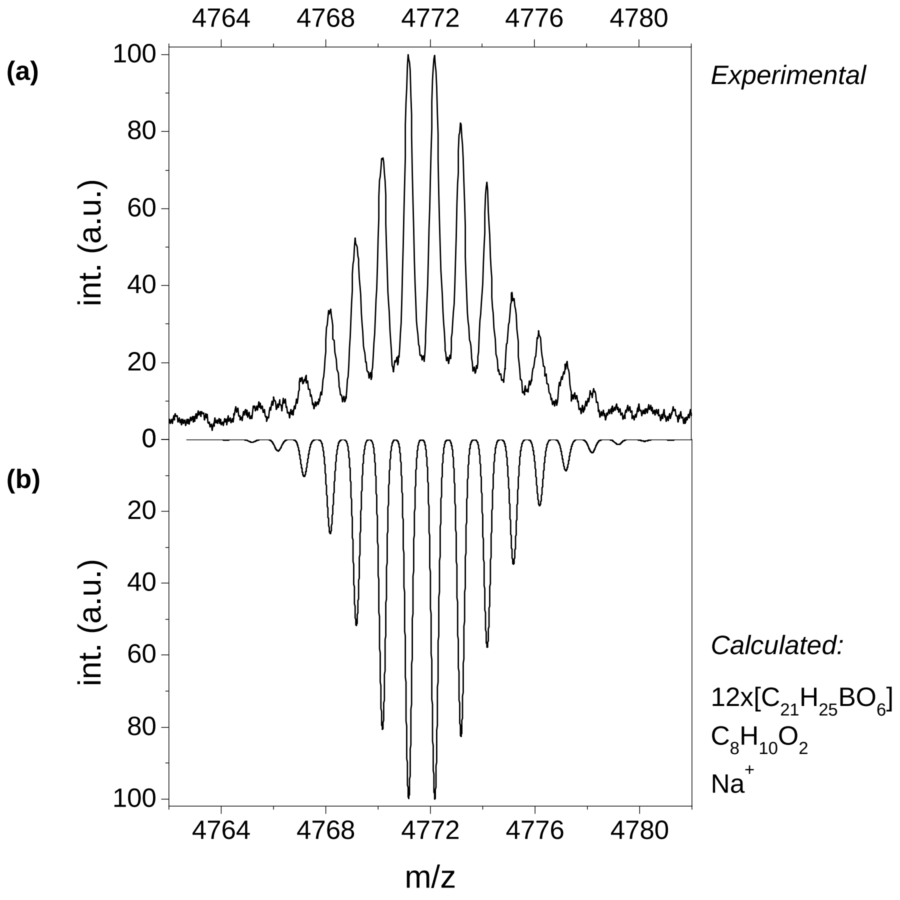

Figure S56. (a) Section of the MALDI-TOF spectrum shown in Figure S54, displaying the isotope distribution pattern, and (b) the calculated isotope distrubitons for a molecular formula of $C_{260}H_{310}B_{12}O_{74}Na$, which is consistent with the 12th-mer repeat unit of the neopentyl glycol ester-protected polymer P[B(NPG)-PA/vCHO] (featuring BDM/proton end-groups and sodium salt).

4.5. Differential Scanning Calorimetry (DSC) of $B(OH)_2$-Polyesters

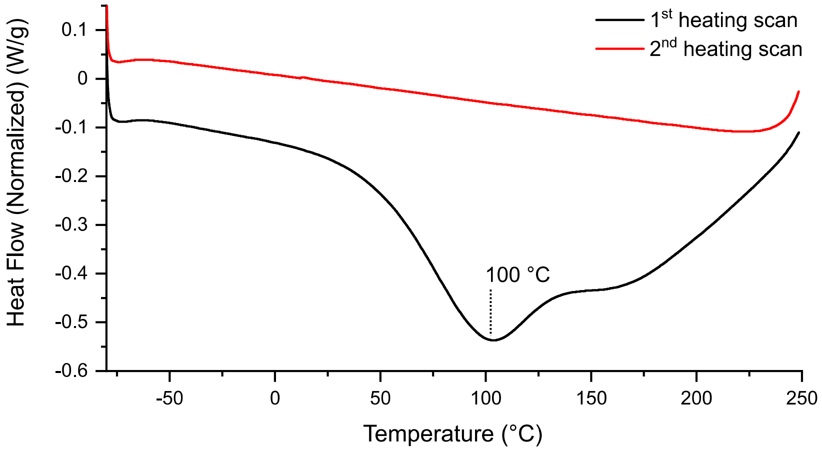

Figure S57. DSC thermograms of $P[B(OH)_2$-PA/CHO] during the first (black line) and second (red line) heating scans, recorded at 10 °C min^{-1}.

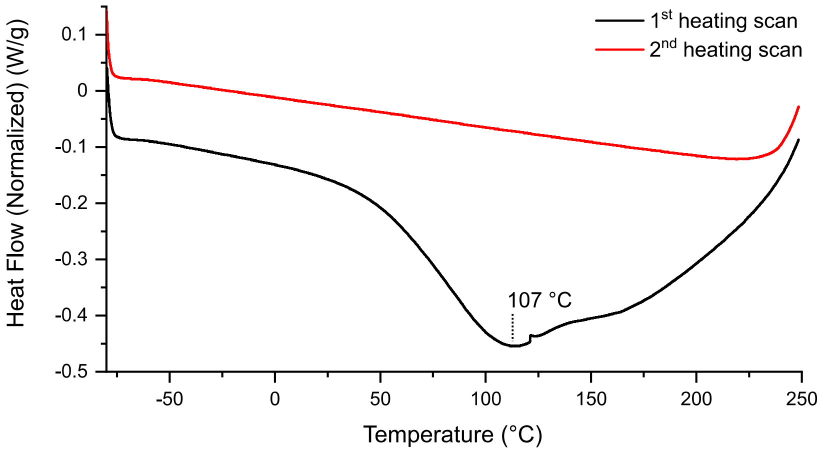

Figure S58. DSC thermograms of P[B(OH)$_2$-PA/vCHO] during the first (black line) and second (red line) heating scans, recorded at 10 °C min^{-1}.

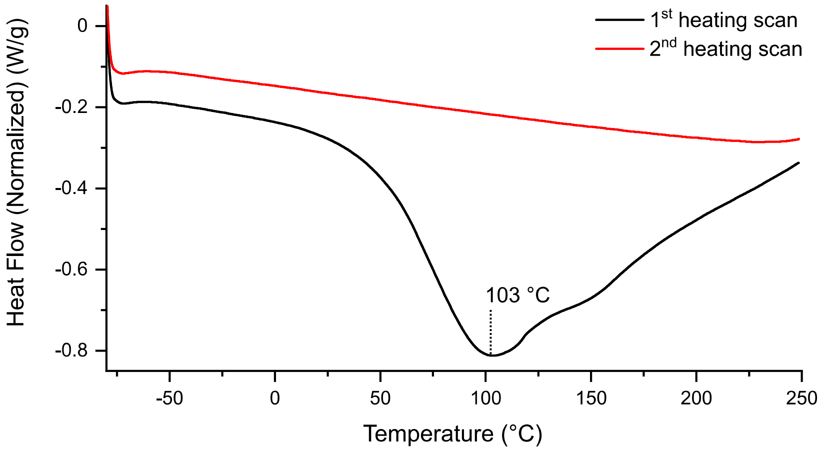

Figure S59. DSC thermograms of P[B(OH)$_2$-PA/PO] during the first (black line) and second (red line) heating scans, recorded at 10 °C min^{-1}.

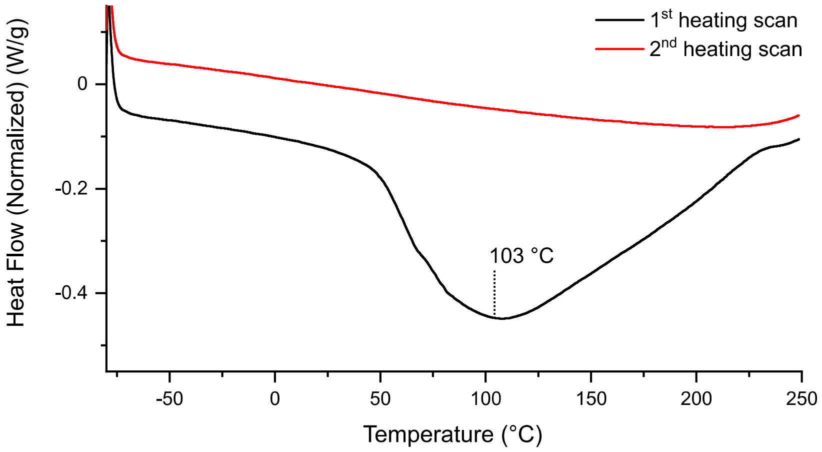

Figure S60. DSC thermograms of P[B(OH)$_2$-PA/AGE] during the first (black line) and second (red line) heating scans, recorded at 10 °C min^{-1}.

4.6. Thermogravimetric Analysis (TGA) of $B(OH)_2$-polyesters

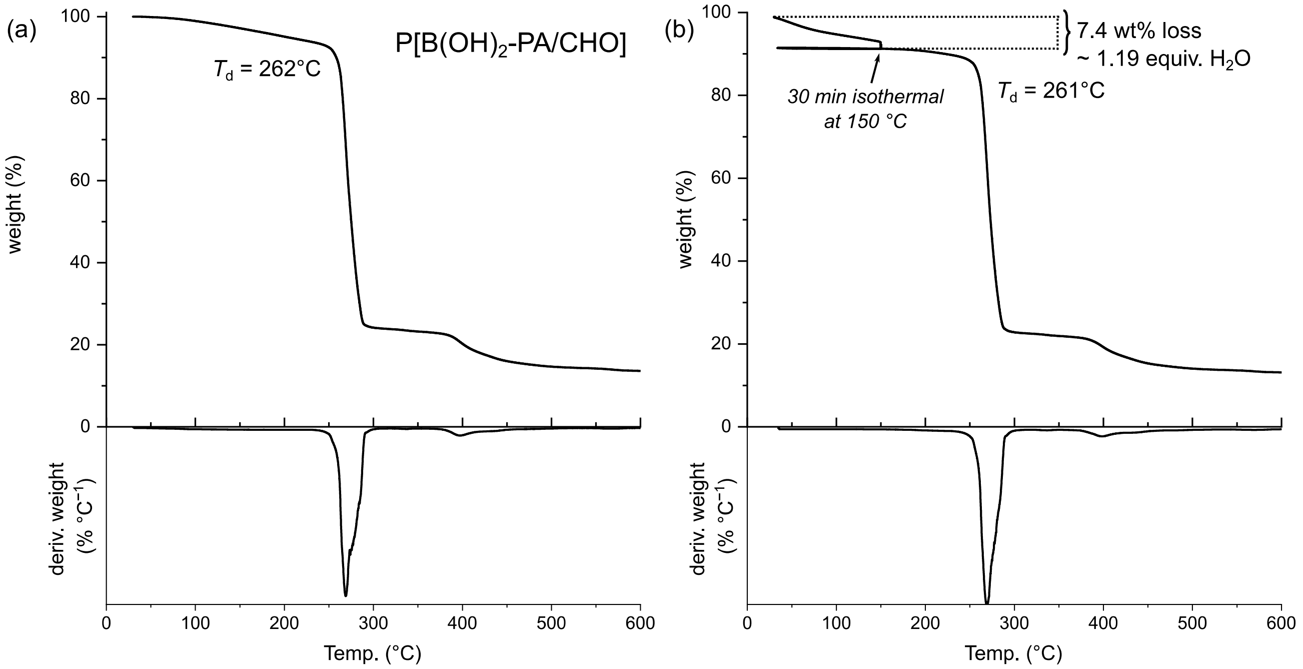

Figure S61. TGA traces of P[$B(OH)_2$-PA/CHO]: (a) direct heating ramp (30 to 600 °C) at 10 °C min^{-1}, and (b) 30 min isothermal at 150 °C, followed by a heating ramp (30 to 600 °C) at 10 °C min^{-1}, showing the loss of 1.19 equiv. H_2O per repeat unit.

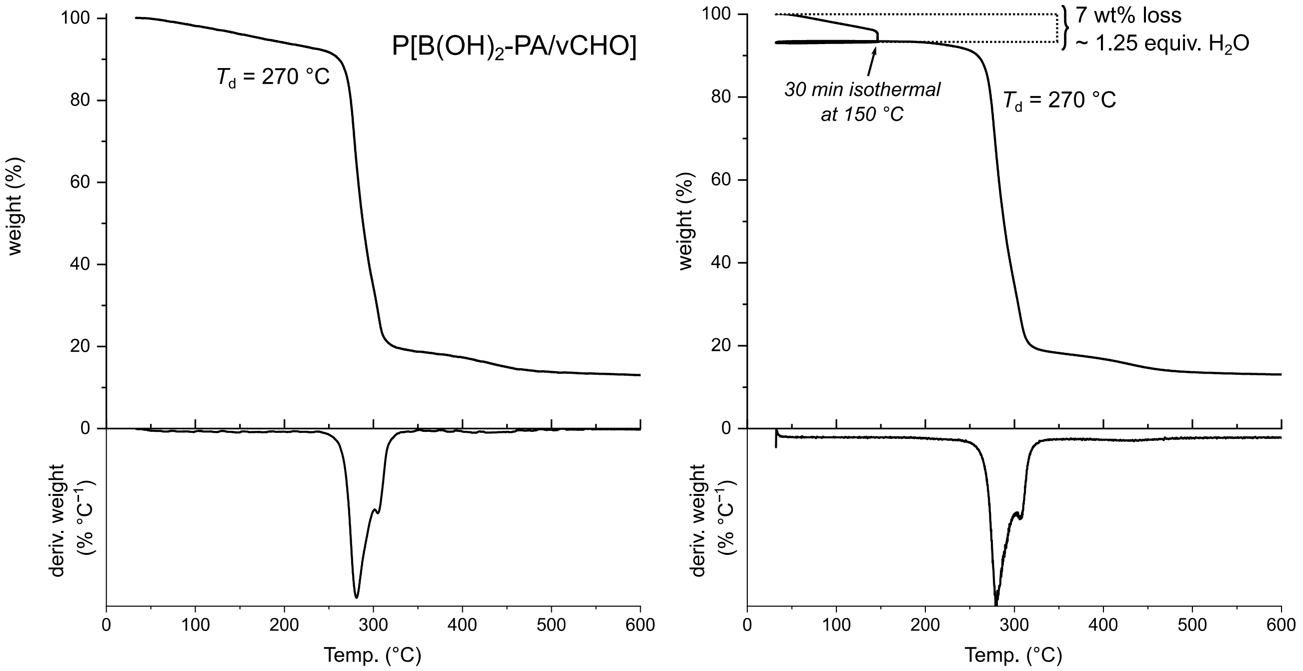

Figure S62. TGA traces of P[$B(OH)_2$-PA/vCHO]: (a) direct heating ramp (30 to 600 °C) at 10 °C min^{-1}, and (b) 30 min isothermal at 150 °C, followed by a heating ramp (30 to 600 °C) at 10 °C min^{-1}, showing the loss of 1.25 equiv. H_2O per repeat unit.

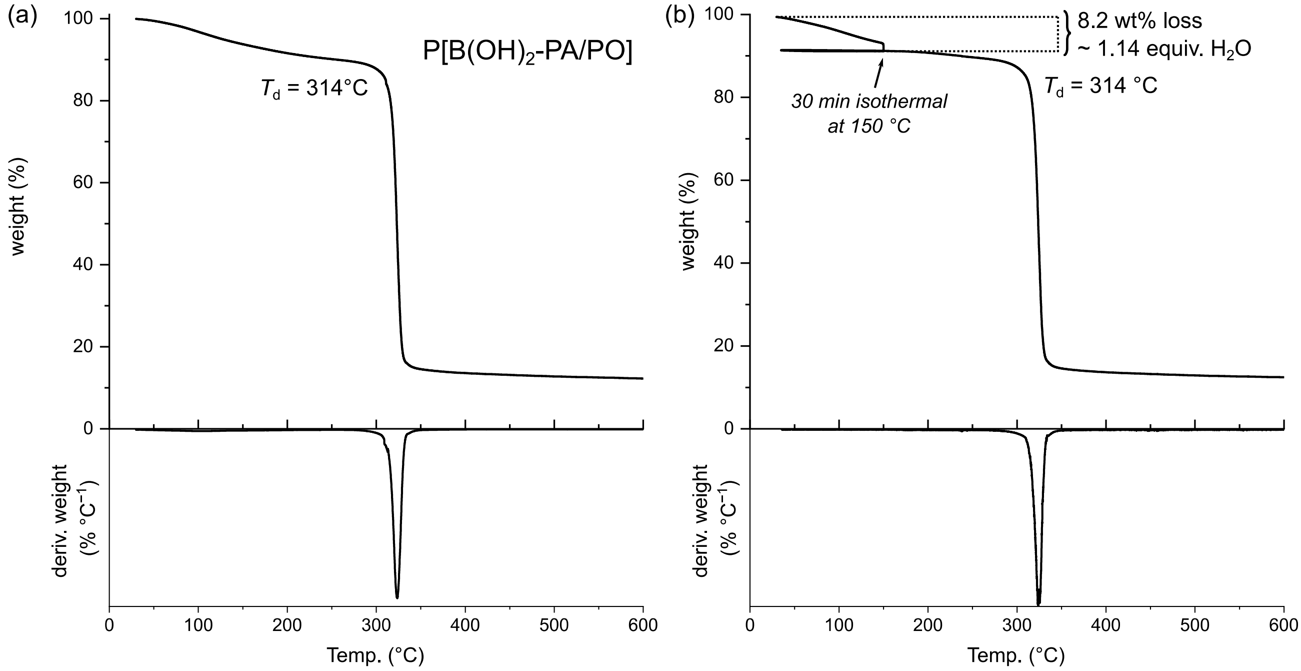

Figure S63. TGA traces of P[B(OH)$_2$-PA/PO]: (a) direct heating ramp (30 to 600 °C) at 10 °C min^{-1}, and (b) 30 min isothermal at 150 °C, followed by a heating ramp (30 to 600 °C) at 10 °C min^{-1}, showing the loss of 1.14 equiv. H_2O per repeat unit.

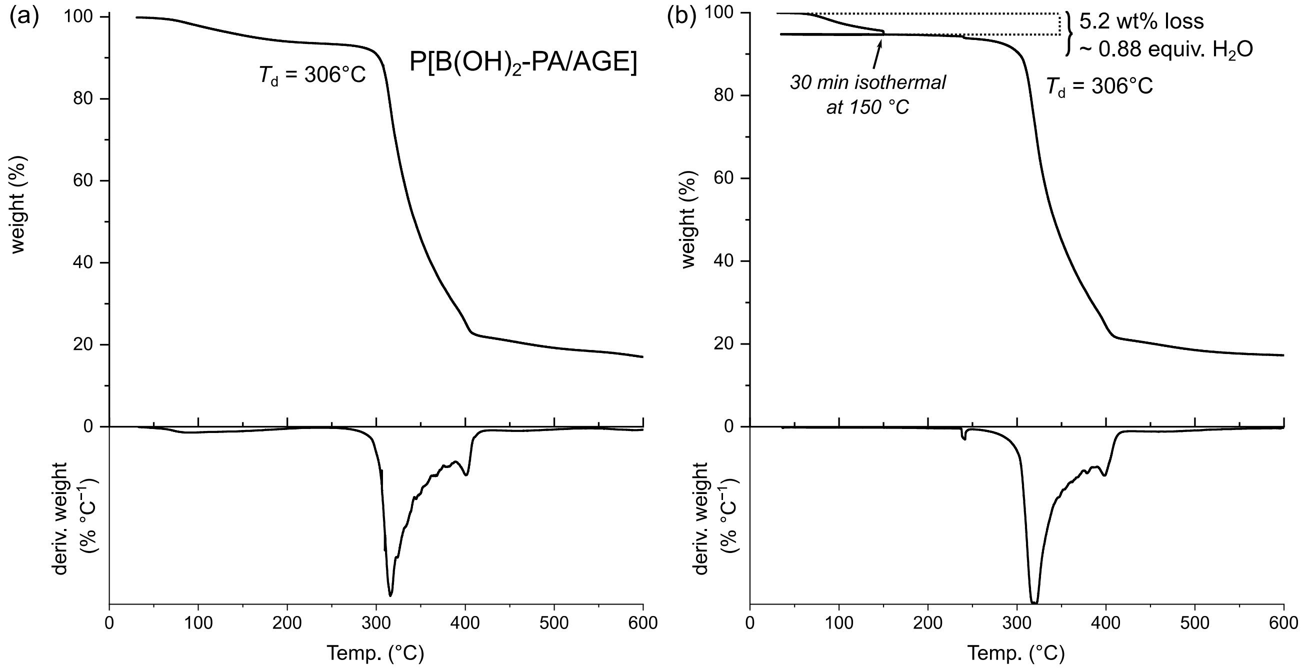

Figure S64. TGA traces of P[B(OH)$_2$-PA/AGE]: (a) direct heating ramp (30 to 600 °C) at 10 °C min^{-1}, and (b) 30 min isothermal at 150 °C, followed by a heating ramp (30 to 600 °C) at 10 °C min^{-1}, showing the loss of 0.88 equiv. H_2O per repeat unit.

5. Synthesis and Characterization of Boron-Block Copolymers (AB and ABC)

5.1. Synthesis of poly(ethylene glycol)-*b*-poly(pinacolboronate phthalate-alt-propylene), *m*PEG-*b*-P(BPin-PA/PO).

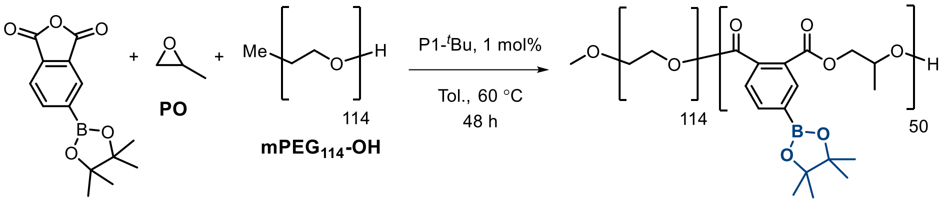

Inside a glovebox, an oven-dried 40 mL reaction vial, with a Teflon-lined screw cap, was loaded with a magnetic stir bar, methylpoly(ethylene glycol) (mPEG–OH) macroinitiator (350 mg, 0.04 mmol, 2 equiv.) and BPin-PA (548 mg, 2.00 mmol, 100 equiv.). Then, propylene oxide (0.21 mL, 3.0 mmol, 150 equiv.) was injected alongside P1-^{t}Bu (5 mg, 0.02 mmol, 1 equiv.) and toluene (3.0 mL) was added so that the final BPin-PA concentration was 1.00 M. The vial was then sealed and placed, with vigorous stirring, into an oil bath preheated to 60 °C. After 48 h, the polymerization was stopped by submersion of the vial in an acetone/dry ice bath and the vial was taken into the glovebox. Analysis of a reaction aliquot, by NMR spectroscopy, indicated quantitative conversion of BPin-PA. At this point, the reaction was diluted with dichloromethane (2 mL), and precipitated into excess diethyl ether (50 mL). The solids were decanted, reprecipitated twice from dichloromethane (5 mL) into excess diethyl ether (50 mL). Finally, the solids were collected, filtered and dried under vacuum, at 60 °C, until constant weight. Yield = 860 mg (85%). The resulting polymer showed a molecular weight (by GPC THF as eluent): M_n = 15.9 kDa, $Đ$ = 1.10.

***m*PEG-*b*-P(BPin-PA/PO).** ^{1}H NMR ($CDCl_3$, 25 ◦C, 499.9 MHz): δ 8.12–8.08 (bs, 1H, Ar-*H*); 7.90 (bs, 1H, Ar-*H*); 7.74–7.62 (bs, 1H, Ar-*H*); 5.40 (bs, 1H, –OC*H*–); 4.40 (bs, 2H, –C*H*$_2$– ester); 3.64 (s, 6.5 × 4H, –C*H*$_2$– ether); 3.37 (s, O–C*H*$_3$– end-group); 1.38 (bs, 3H, –C*H*$_3$); 1.30 (bs, 12H, –BPin).

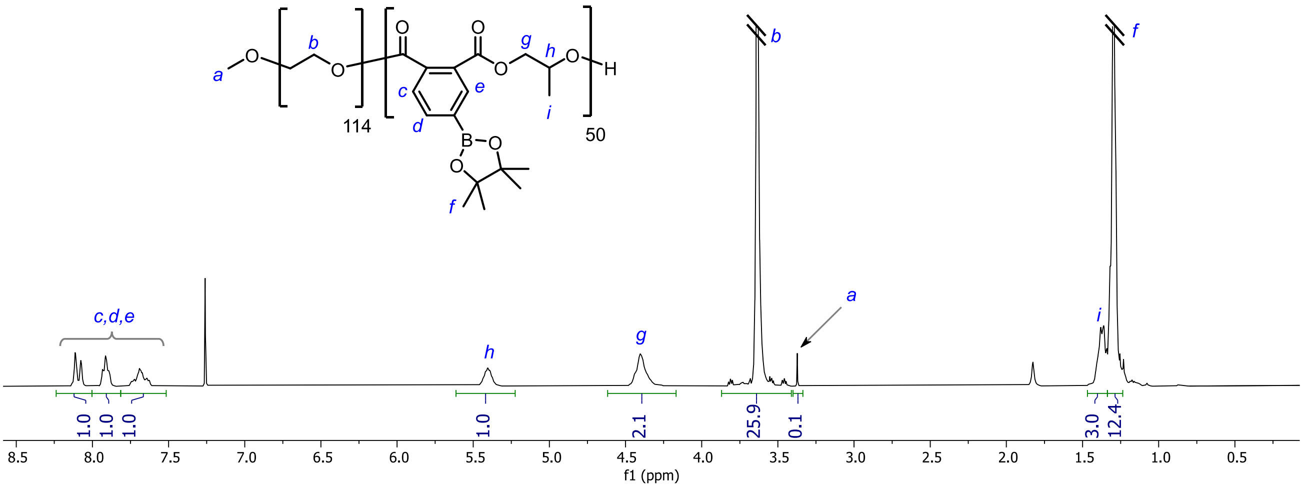

Figure S65. ^{1}H NMR ($CDCl_3$, 25 °C, 499.9 MHz) spectrum of isolated *m*PEG-*b*-P(BPin-PA/PO).

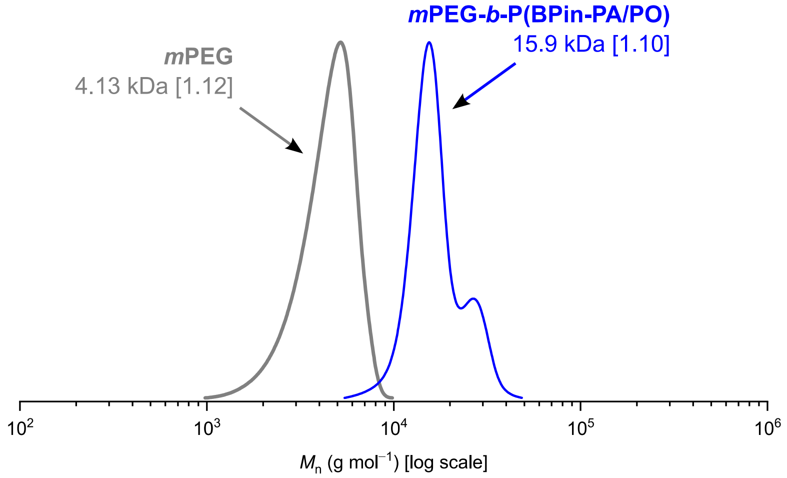

Figure S66. Comparison of GPC traces of isolated *m*PEG-*b*-P(BPin-PA/PO) and its precursor mPEG–OH macroinitiator. The slight bimodality in the GPC trace of *m*PEG-*b*-P(BPin-PA/PO) is attributed to residual mono- and di-functional protic impurities which results in competitive initiation with the *m*PEG.

5.2. Synthesis of poly(ethylene glycol)-*b*-poly(boronic acid phthalate-alt-propylene), *m*PEG-*b*-P[B(OH)$_2$-PA/PO].

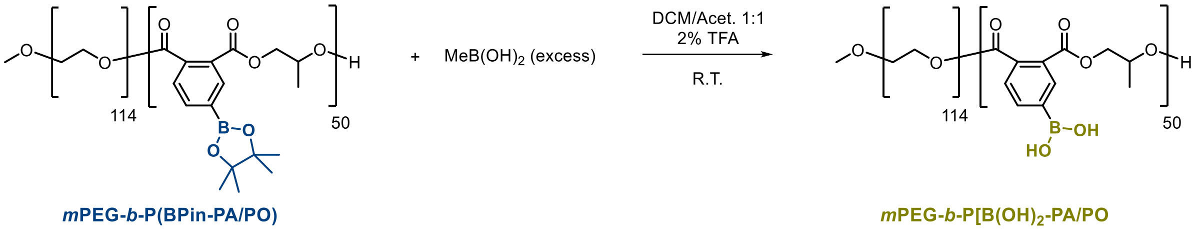

In a 50 mL round-bottom flask, a sample of *m*PEG-*b*-P(BPin-PA/PO) (270 mg, 0.5 mmol in boron units) and methylboronic acid (620 mg, 10 mmol, 20 equiv.) were loaded and dissolved in a mixture of acetone/CH_2Cl_2 1:1 (v:v) so that the final concentration was 0.10 M. To this solution was added trifluoroacetic acid (2%) and the resulting homogeneous and clear solution was stirred, at room temperature, for 20 h. Then, the volatiles were removed, under vacuum at 40 °C, in the rotavapor for 1 h. The resulting residue was redissolved in acetone (2 mL), and the solution evaporated, at 60 °C in the rotavapor, for 2 hours. The resulting powder was redissolved in acetone (2 mL) and precipitated thrice over a large excess of Et_2O (3 × 100 mL); the polymer was then separated by centrifugation, and dried under vacuum, at 60 °C, until constant weight. Yield = 216 mg (95%).

***m*PEG-*b*-P[B(OH)$_2$-PA/PO].** ^{1}H NMR [$(CD_3)_2CO$, 25 ◦C, 499.9 MHz]: δ 8.10–7.61 (bs, Ar-*H*); 5.30 (bs, –OC*H*–); 4.71 (bs, –C*H*$_2$– ester); 3.62 (s,–C*H*$_2$– ether); 2.90 (bs, B–O*H*); 1.25 (bs,–C*H*$_3$).

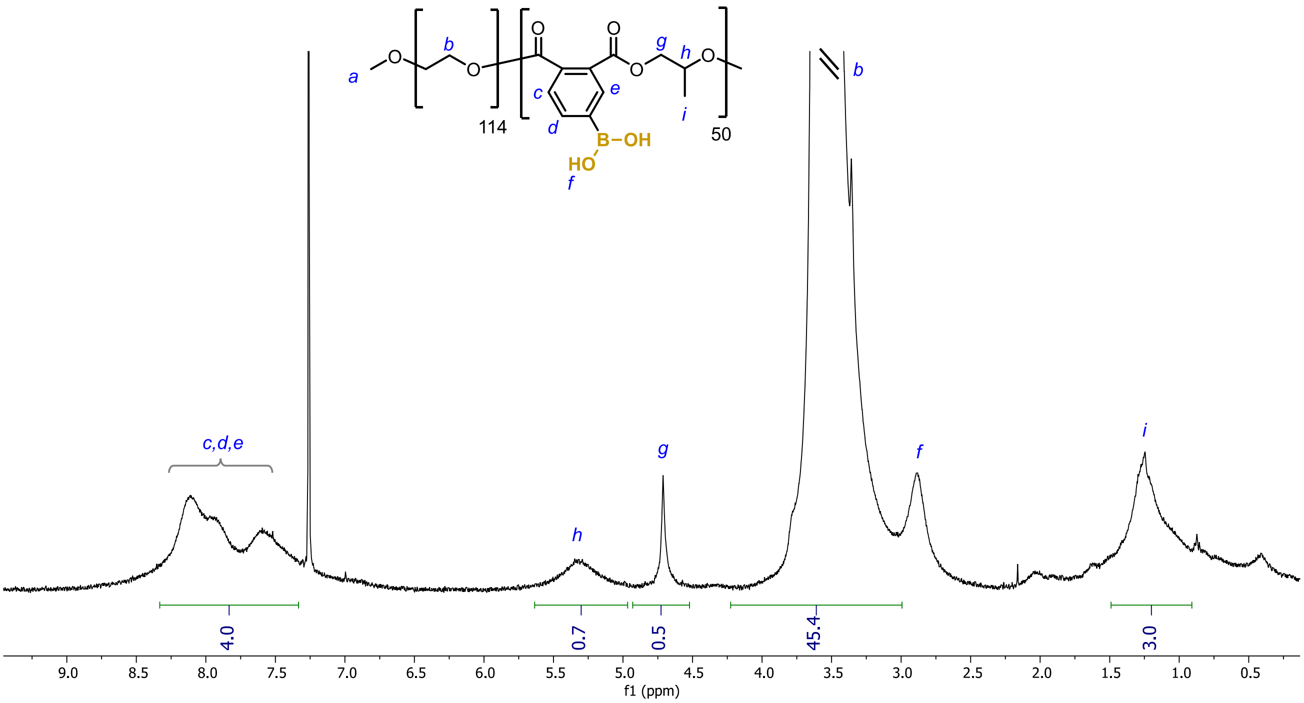

Figure S67. ^{1}H NMR ($CDCl_3$, 25 °C, 400.2 MHz) spectrum of isolated *m*PEG-*b*-P[B(OH)$_2$-PA/PO].

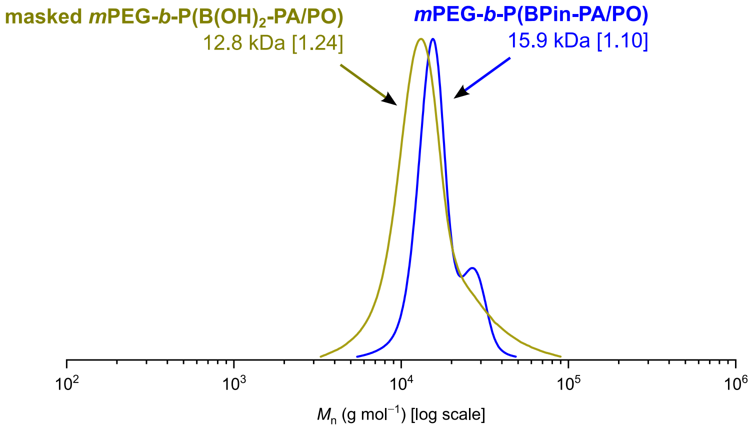

Figure S68. Comparison of GPC traces of isolated *m*PEG-*b*-P[B(OH)$_2$-PA/PO] (*in-situ* masked with NPG) and its precursor.

5.3. Synthesis of poly(ethylene glycol)-*b*-poly(ε-decalactone)-*b*-poly(phthalate/pinacolboronate phthalate-alt-cyclohexene), *m*PEG-*b*-PDL-*b*-P(BPin-PA/CHO-*ran*-PA/CHO)

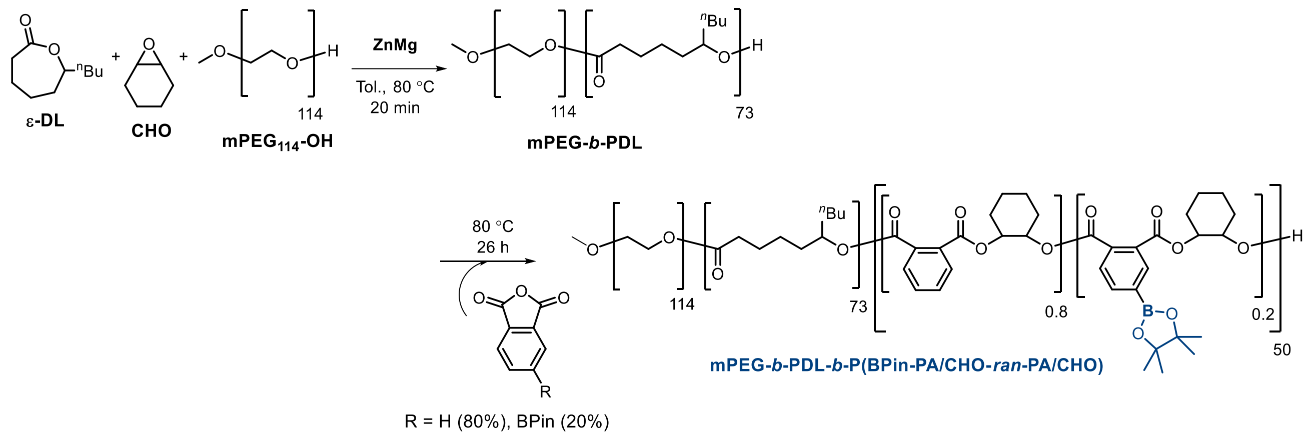

Inside a glovebox, an oven-dried 40 mL reaction vial, with a Teflon-lined screw cap, was loaded with a magnetic stir bar, methylpoly(ethylene glycol) (*m*PEG–OH) macroinitiator (500 mg, 0.07 mmol, 4 equiv.), cyclohexene oxide (0.56 mL, 5.5 mmol, 400 equiv.), and epsilon-decalactone (0.77 mL, 4.4 mmol, 315 equiv.). Then, a solution of [ZnMg] (13 mg, 0.014 mmol, 1 equiv.) in toluene (1.0 mL) was added and the final reaction mixture diluted with toluene (2.5 mL). The vial was then sealed and placed, under vigorous stirring, in an oil bath, preheated to 80 °C. After 20 min, the polymerization was stopped by submersion of the vial in an acetone/dry ice bath and the vial was taken into the glovebox. Analysis of a reaction aliquot, by NMR spectroscopy, indicated 93 % conversion of epsilon-decalactone into *m*PEG-*b*-PDL di-block copolymer (M_n = 13.6 kDa, *Đ* = 1.22). At this point, BPin-PA (152 mg, 0.55 mmol, 40 equiv.), and PA (327 mg, 2.21 mmol, 160 equiv.) were added to the reaction mixture. The vial was once again sealed and placed, under vigorous stirring, in an oil bath preheated to 80 °C. After 26 h, the polymerization was stopped by submersion of the vial in an acetone/dry ice bath and the vial was taken into the glovebox. Analysis of a reaction aliquot, by NMR spectroscopy, indicated quantitative conversions of both BPin-PA and PA into *m*PEG-*b*-PDL-*b*-P(BPin-PA/CHO) triblock copolymer (M_n = 20.4 kDa, *Đ* = 1.19). At this point, the reaction was diluted with dichloromethane (3 mL), and precipitated into excess diethyl ether (50 mL). The solids were decanted, reprecipitated twice from dichloromethane (5 mL) into excess diethyl ether (50 mL). Finally, the solids were collected, filtered and dried under vacuum, at 60 °C, until constant weight. Yield = 1550 mg (85%). The polymer molecular weight (by GPC with THF as eluent): M_n = 20.4 kDa, *Đ* = 1.19.

***m*PEG-*b*-PDL-*b*-P(BPin-PA/CHO-*ran*-PA/CHO).** ^{1}H NMR ($CDCl_3$, 25 ◦C, 400.2 MHz): δ 8.01 (d, *J* = 12.8 Hz, BPin-Ar-*H*); 7.84 (bs, BPin-Ar-*H*); 7.58 (bs, 1H, PA-Ar-*H*); 7.38 (bs, 1H, PA-Ar-*H*); 5.13 (bs, 1H, –OC*H*–); 4.85 (q, 1H, *J* = 6.2 Hz, PDL–OC*H*–); 3.63 (s, 4H, PEG–OCH_2–), 2.26 (t, *J* = 7.4 Hz, 2H, PDL–COCH_2–), 2.23–1.27 (–CH_2–, overlapping –BPin), 0.87 (t, *J* = 6.8 Hz, 3H, PDL–CH_3).

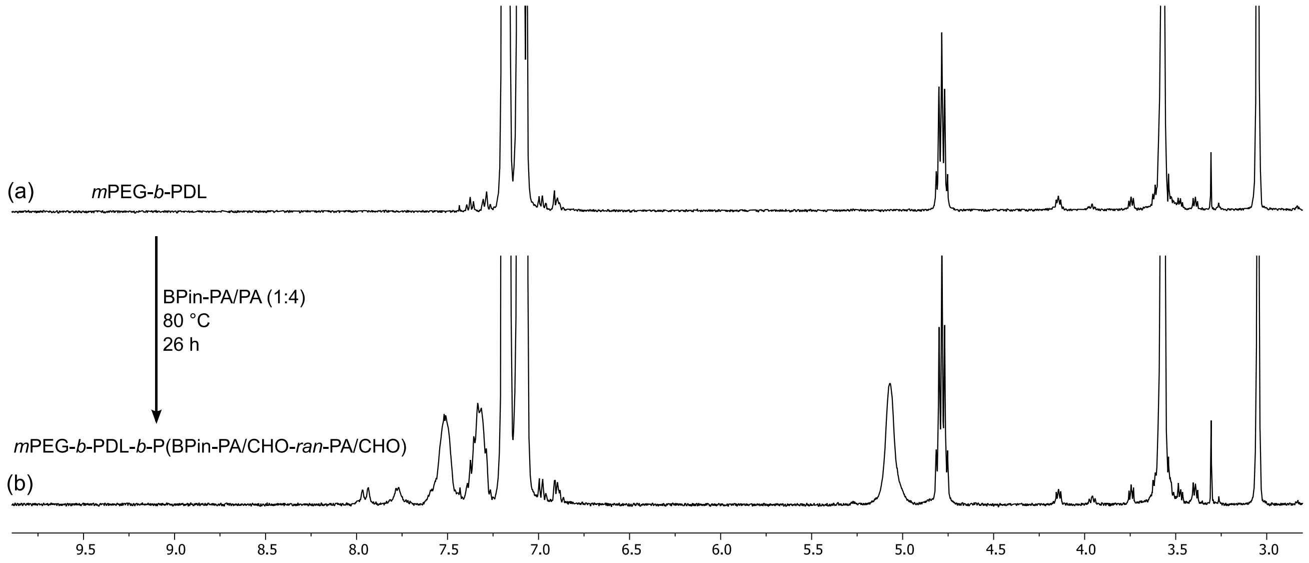

Figure S69. ^{1}H NMR ($CDCl_3$, 25 °C, 400.2 MHz) spectra of reaction aliquots in the formation of (a) *m*PEG-*b*-PDL and (b) *m*PEG-*b*-PDL-*b*-P(BPin-PA/CHO-*ran*-PA/CHO). Note that lack of monomer signals at > 8 ppm which indicates the complete conversion of the anhydrides.

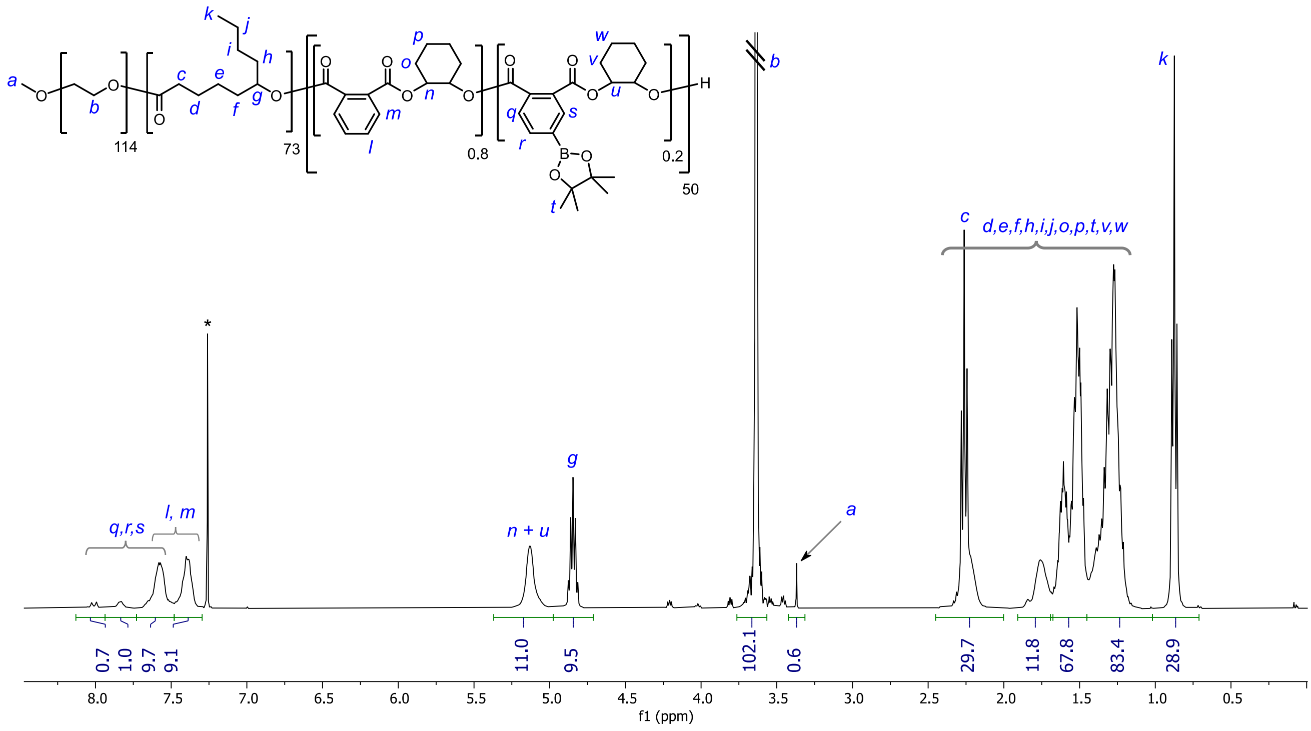

Figure S70. ^{1}H NMR ($CDCl_3$, 25 °C, 400.2 MHz) spectrum of isolated *m*PEG-*b*-PDL-*b*-P(BPin-PA/CHO-*ran*-PA/CHO).

6. Suzuki-Miyaura Cross-Coupling Post-Polymerization Modification

6.1. Synthesis of poly(phthalate/BODIPY-alt-cyclohexene), P(BODIPY-PA/CHO).

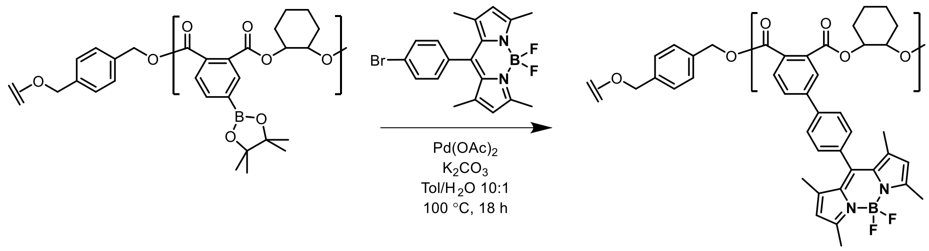

Inside a N_2-filled glovebox, an oven-dried Schlenk flask was loaded with P(BPin-PA/CHO) (100 mg, 0.27 mmol -BPin units), Br-BODIPY (130 mg, 0.27 mmol), K_2CO_3 (112 mg, 0.81 mmol), $Pd(OAc)_2$ (6 mg, 0.027 mmol), SPHOS (22 mg, 0.054 mmol), and the mixture was suspended in toluene (5 mL). The flask was taken outside the glovebox, interfaced on a Schlenk-line, at which point H_2O (50 µL) was added, under a stream of N_2. The flask was securely sealed, and the reaction mixture was freeze/pump/thawed thrice to remove all oxygen. Then, the reaction was heated to 100 °C and allowed to react, under vigorous stirring, for 18 h. Upon cooling, a reaction aliquot was taken under inert atmosphere; analysis by ^{1}H and ^{19}F NMR spectroscopy of the aliquot indicated complete conversion of the boronic ester groups. The reaction mixture was then diluted with chloroform (10 mL), dried with Na_2SO_4, and passed through a small plug of celite. The filtrate was collected in a small round bottomed flask, the volatiles removed under vacuum, and the residue redissolved in dichloromethane (3 mL). The polymer was then precipitated by addition into a large excess of methanol (3 × 150 mL), then hexanes (3 × 150 mL), and finally dried, under vacuo at 50 °C, until constant weight. Yield = 100 mg (65%). The resulting polymer was obtained as a bright red powder and its molecular weight (by GPC with THF as eluent): M_n = 12.0 kDa, $Đ$ = 1.50.

P(BODIPY-PA/CHO). ^{1}H NMR ($CDCl_3$, 25 ◦C, 400.2 MHz): δ 7.79–7.21 (bs, 7H, Ar-*H*); 5.91 (s, 2H, pyr-*H*); 5.16 (bs, 1H, –OC*H*–); 2.50 (s, 6H, pyr–CH_3); 2.25–1.14 (–CH_2–, overlapping pyr–CH_3). ^{19}F NMR ($CDCl_3$, 25 °C, 376.5 MHz): δ −146.0. $^{11}B\{^{1}H\}$ NMR ($CDCl_3$, 25 °C, 128.4 MHz): δ 0.7.

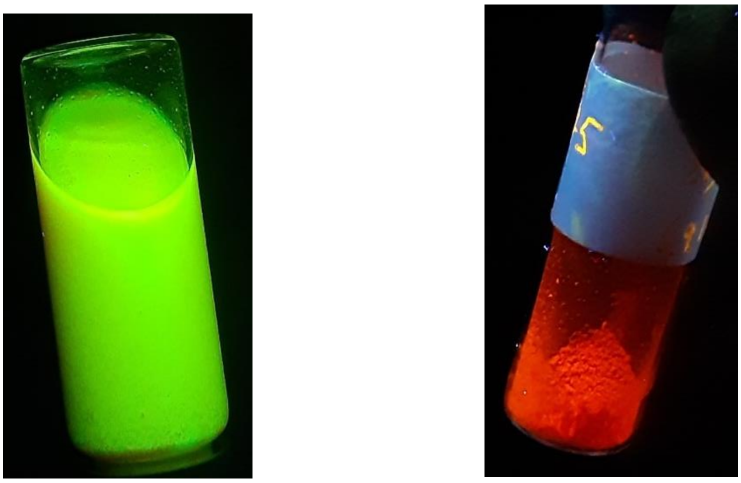

Figure S71. Photographs of P(BODIPY-PA/CHO) under UV irradiation (left, MeOH suspension); right, solid powder).

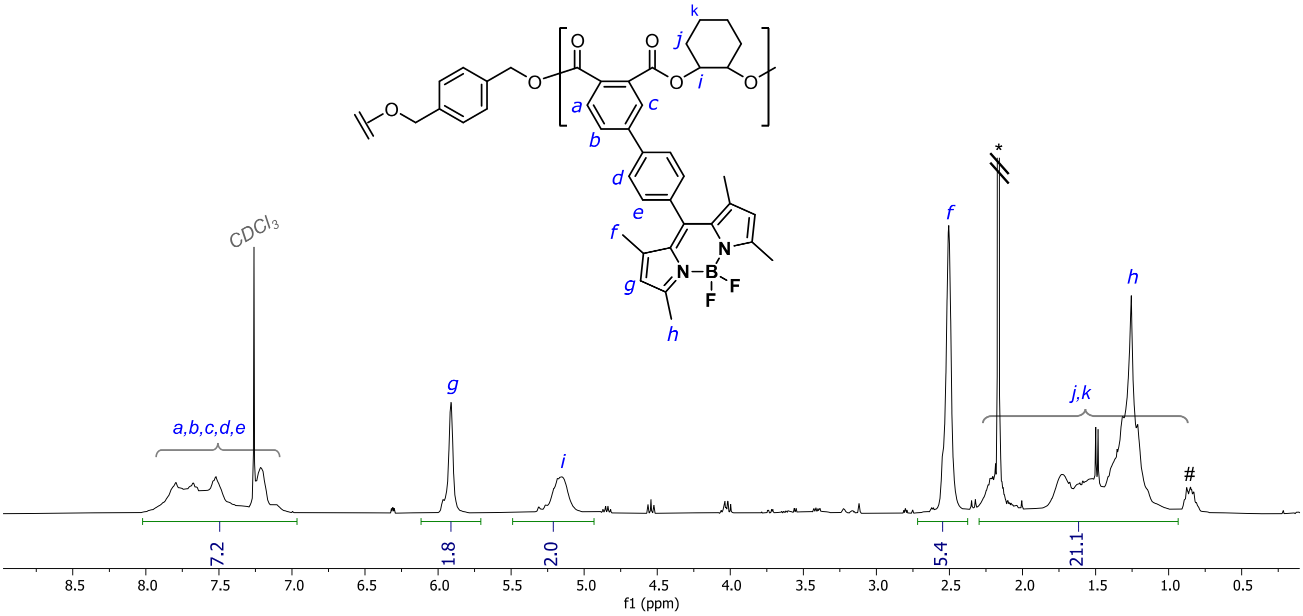

Figure S72. ^{1}H NMR ($CDCl_3$, 25 °C, 400.2 MHz) spectrum of P(BODIPY-PA/CHO). (* = acetone; # = hexanes).

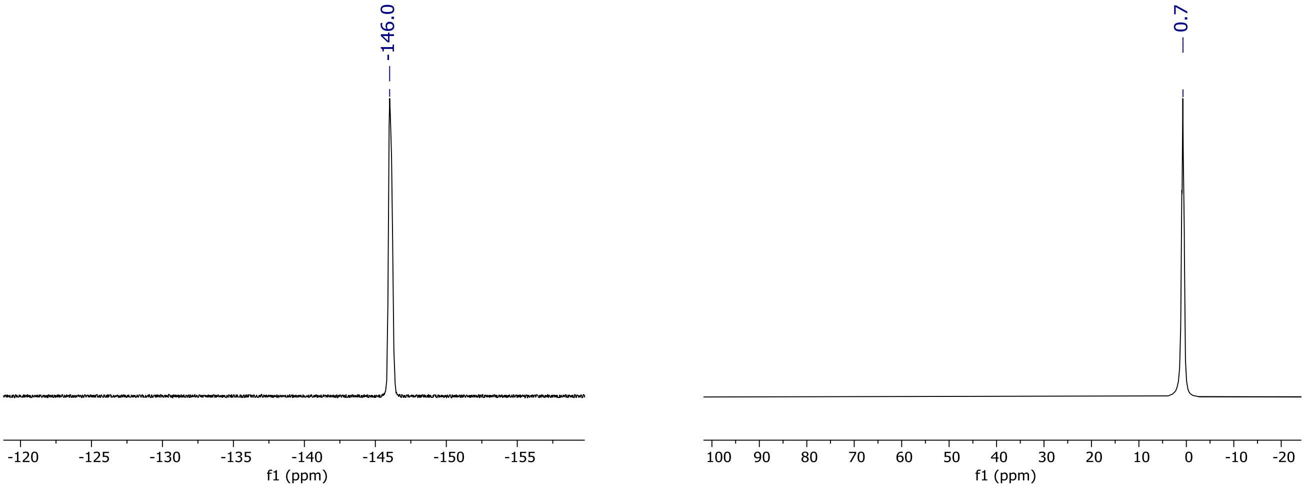

Figure S73. (left) ^{19}F NMR ($CDCl_3$, 25 °C, 376.5 MHz) and (right) ^{11}B{^{1}H} NMR ($CDCl_3$, 25 °C, 128.4 MHz) spectra of isolated P(BODIPY-PA/CHO).

6.2. Synthesis of poly(ethylene glycol)-*b*-poly(ε-decalactone)-*b*-poly(phthalate/BODIPY phthalate-alt-cyclohexene), *m*PEG-*b*-PDL-*b*-P(BODIPY-PA/CHO-*ran*-PA/CHO).

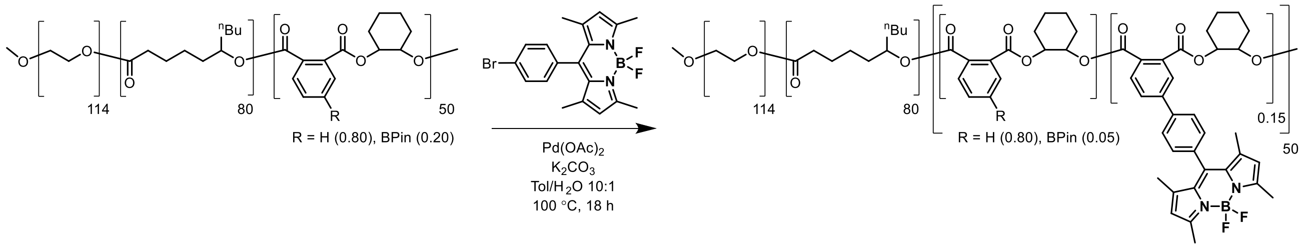

Inside a N_2-filled glovebox, an oven-dried Schlenk flask was loaded with *m*PEG-*b*-PDL-*b*-P(BPin-PA/CHO) (500 mg, 0.065 mmol -BPin units), Br-BODIPY (28 mg, 0.068 mmol), K_2CO_3 (27 mg, 0.20 mmol), $Pd(OAc)_2$ (2 mg, 0.007 mmol), SPHOS (5 mg, 0.001 mmol), and the mixture was suspended in toluene (3 mL). The flask was taken outside the glovebox, interfaced on a Schlenk-line, at which point H_2O (30 µL) was added, under a stream of N_2. The flask was securely sealed, and the reaction mixture was freeze/pump/thawed thrice to remove all oxygen. Then, the reaction was heated to 100 °C and allowed to react, under vigorous stirring, for 18 h. Upon cooling, a reaction aliquot was taken under inert atmosphere; analysis by ^{1}H and ^{19}F NMR spectroscopy of the aliquot indicated full conversion of the boronic ester groups. The reaction mixture was then diluted with chloroform (10 mL), dried with Na_2SO_4, and passed through a small plug of celite. The filtrate was collected in a small round bottomed flask, the volatiles removed under vacuum, and the residue redissolved in dichloromethane (3 mL). The polymer was then precipitated by addition into a large excess of diethyl ether (3 × 150 mL), then hexanes (3 × 150 mL). The final polymer was dissolved in THF/H_2O (10 mL, 1:9), filtered through a syringe filter, and the solvents allowed to evaporate. The final polymer was then dried, under vacuo at 50 °C, until constant weight. Yield = 220 mg (38%). Molecular weight (by GPC with THF as eluent): M_n = 25.0 kDa, $Đ$ = 1.30.

m**PEG-*****b*****-PDL-*****b*****-P(BODIPY-PA/CHO-*****ran*****-PA/CHO).** ^{1}H NMR ($CDCl_3$, 25 ◦C, 400.2 MHz): δ 8.02–7.25 (bs, overlapping –BPin and –BODIPY Ar-*H*); 7.60 (bs, 2H, phthalic Ar-*H*); 7.41 (bs, 2H, phthalic Ar-*H*); 5.99

(s, pyr-*H*); 5.16 (bs, 1H, –OC*H*–); 4.85 (q, 1H, *J* = 6.2 Hz, PDL–OC*H*–); 3.66 (s, 4H, PEG–OCH_2–), 2.57 (s, pyr–CH_3); 2.29i (t, *J* = 7.4 Hz, 2H, PDL–COCH_2–), 2.25–1.27 (–CH_2–, overlapping pyr–CH_3), 0.90 (t, *J* = 6.8 Hz, 3H, PDL–CH_3). ^{19}F NMR ($CDCl_3$, 25 °C, 376.5 MHz): δ −146.3. ^{11}B{^{1}H} NMR ($CDCl_3$, 25 °C, 128.4 MHz): δ 0.9.

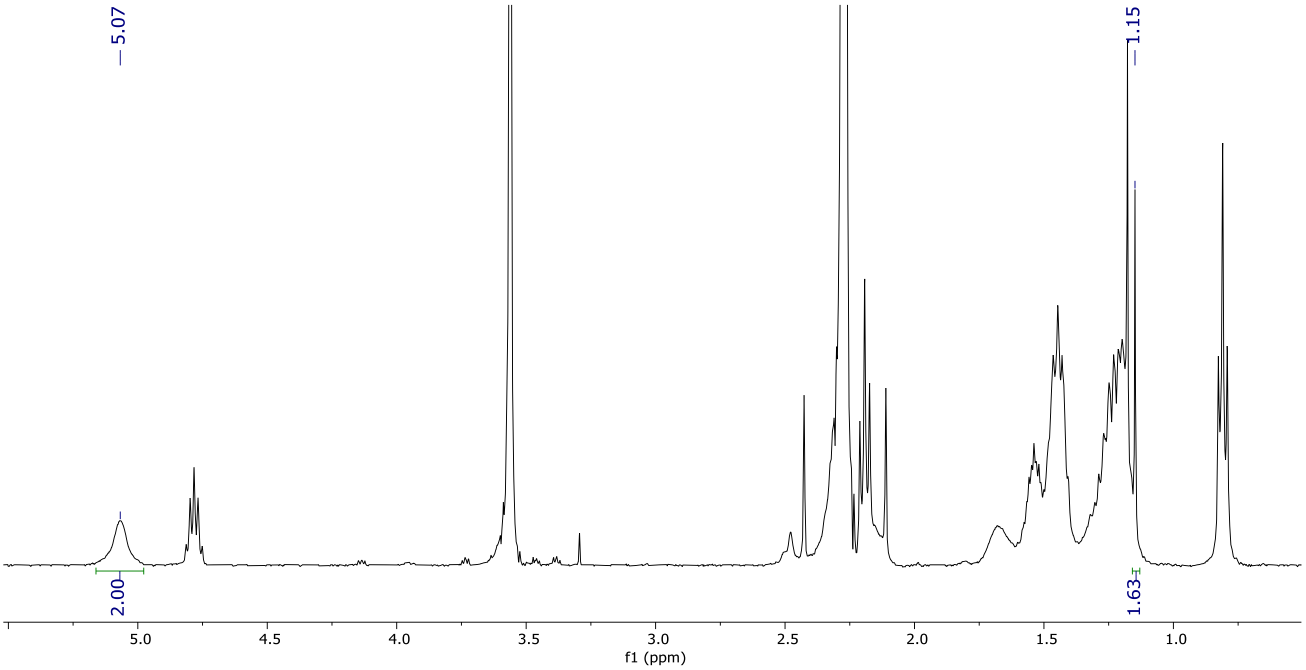

Figure S74. ^{1}H NMR ($CDCl_3$, 25 °C, 400.2 MHz) spectrum of the reaction aliquot during the synthesis *m*PEG-*b*-PDL-*b*-P(BODIPY-PA/CHO-*ran*-PA/CHO). The signal at 1.15 ppm corresponds to released OAc-BPin; integral comparison with the methylene protons of the polymer main-chain provides an estimate of the reaction conversion of the -BPin moieties of ~70%.

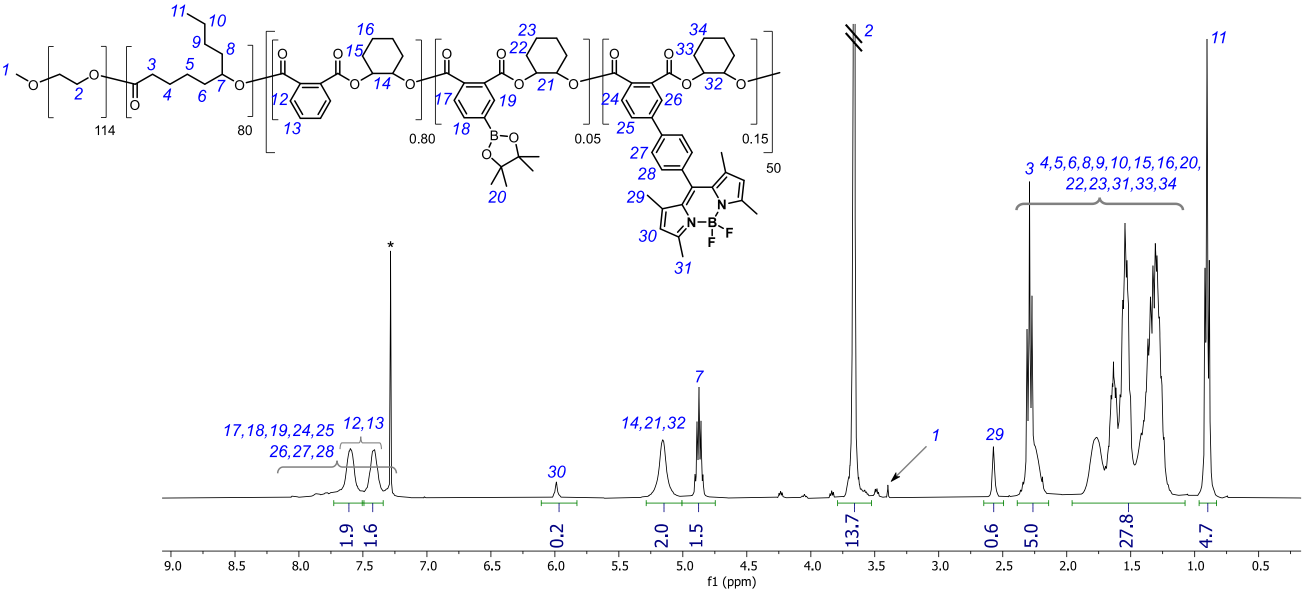

Figure S75. ^{1}H NMR ($CDCl_3$, 25 °C, 400.2 MHz) spectrum of isolated *m*PEG-*b*-PDL-*b*-P(BODIPY-PA/CHO-*ran*-PA/CHO). (* = $CDCl_3$).

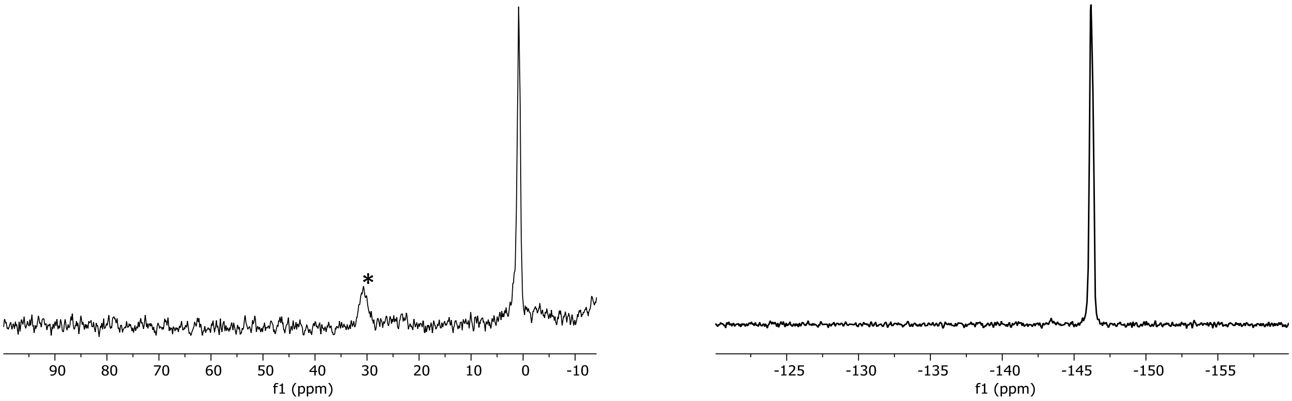

Figure S76. (left) $^{11}B\{^1H\}$ NMR ($CDCl_3$, 25 °C, 128.4 MHz) and (right) ^{19}F NMR ($CDCl_3$, 25 °C, 376.5 MHz) spectra of isolated *m*PEG-*b*-PDL-*b*-P(BODIPY-PA/CHO-*ran*-PA/CHO). (* = residual –BPin)

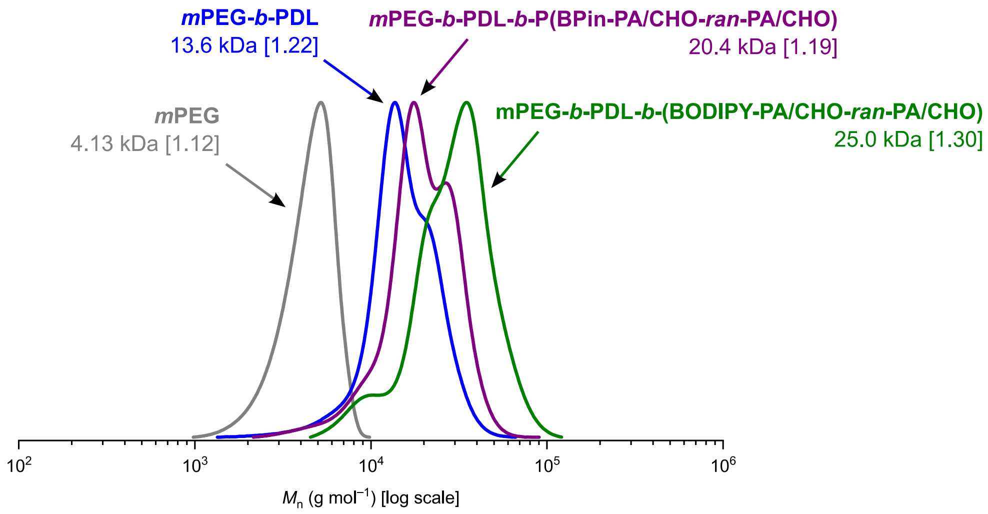

Figure S77. Comparison of GPC traces of isolated *m*PEG-*b*-PDL-*b*-P(BODIPY-PA/CHO-*ran*-PA/CHO) and its precursors.

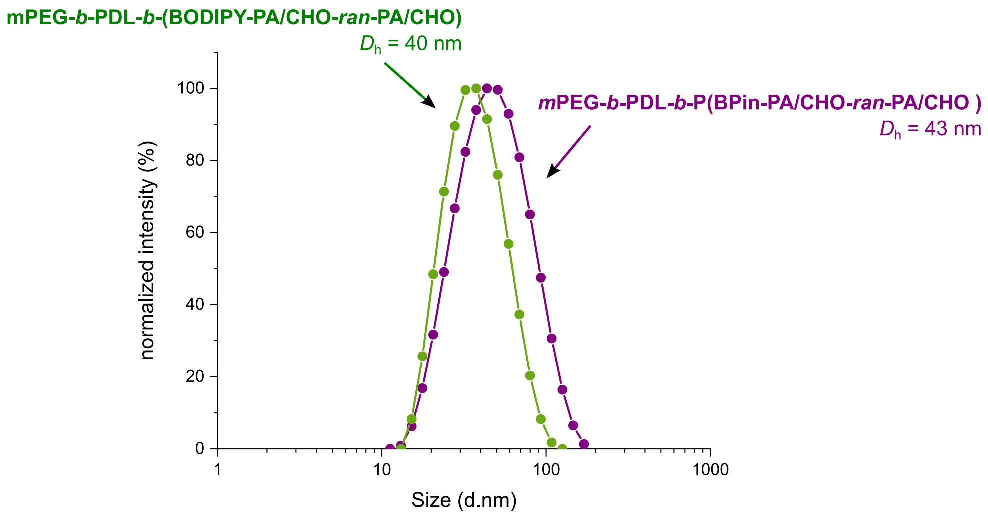

Figure S78. Comparison of the particle size distributions measured by dynamic light scattering (DLS) of the polyester nanoparticles.

7. Aqueous Solution Studies of Boronic Acid-Polymers

7.1. NMR scale investigations in water

In an Eppendorf tube, dry polymer samples of $B(OH)_2$ polyesters (~12 mg) were suspended in D_2O (0.5 mL). Then, 1 equiv. of NaOD with respect to boronic acid units was added using a micropipette from a NaOD/D_2O solution (40 wt%). The mixture was vortexed until a clear solution was obtained, and the resulting solution was analysed by NMR spectroscopy.

P[B(OH)$_3$-PA/CHO]. ^{1}H NMR (D_2O, 25 °C, 499.9 MHz): δ 7.76–7.61 (bs, 3H, Ar-*H*); 5.08 (bs, 2H, –OC*H*–); 3.36 (bs, 2H, B–O*H*); 2.09 (bs, 2H, –C*H*$_2$–); 1.62–1.23 (bs, 6H, –C*H*$_2$–). ^{11}B{^{1}H} NMR (D_2O, 25 °C, 160.4 MHz): δ 1.9.

P[B(OH)$_3$-PA/vCHO]. ^{1}H NMR (D_2O, 25 °C, 499.9 MHz]: δ 7.98–7.53 (bs, 3H, Ar-*H*); 5.77 (bs, 1H, –C*H*=C); 5.35–4.89 (m, 4H, –OC*H*– and C=C*H*$_2$); 3.80 (bs, 2H, B–O*H*); 2.62–1.06 (bs, 8H, –C*H*– and –C*H*$_2$–). ^{11}B{^{1}H} NMR (D_2O, 25 °C, 160.4 MHz): δ 2.0.

P[B(OH)$_3$-PA/PO]. ^{1}H NMR (D_2O, 25 °C, 499.9 MHz): δ 7.84 (bs, 1H, Ar-*H*); 7.77 (bs, 1H, Ar-*H*); 7.63 (bs, 1H, Ar-*H*); 7.54 (s, Ar-*H* 1,4-BDM end-group); 5.18 (bs, 1H, –OC*H*–); 4.24 (bs, 2H, –C*H*$_2$–); 1.19 (bs, 12H, –C*H*$_3$). ^{11}B{^{1}H} NMR (D_2O, 25 °C, 160.4 MHz): δ 1.9.

P[B(OH)$_3$-PA/AGE]. ^{1}H NMR (D_2O, 25 °C, 499.9 MHz): δ 7.77 (bs, 1H, Ar-*H*); 7.72 (bs, 1H, Ar-*H*); 7.61 (bs, 1H, Ar-*H*); 7.46 (s, Ar-*H* 1,4-BDM end-group); 5.73 (bs, 1H, –C*H*=C); 5.35–5.28 (bs, 1H, –OC*H*–); 5.14–5.07 (bs, 2H, C=C*H*$_2$); 4.40 (bs, 2H, O–C*H*$_2$); 3.88 (bs, 2H, O–C*H*$_2$); 3.60 (bs, 2H, O–C*H*$_2$). ^{11}B{^{1}H} NMR (D_2O, 25 °C, 160.4 MHz): δ 2.3.

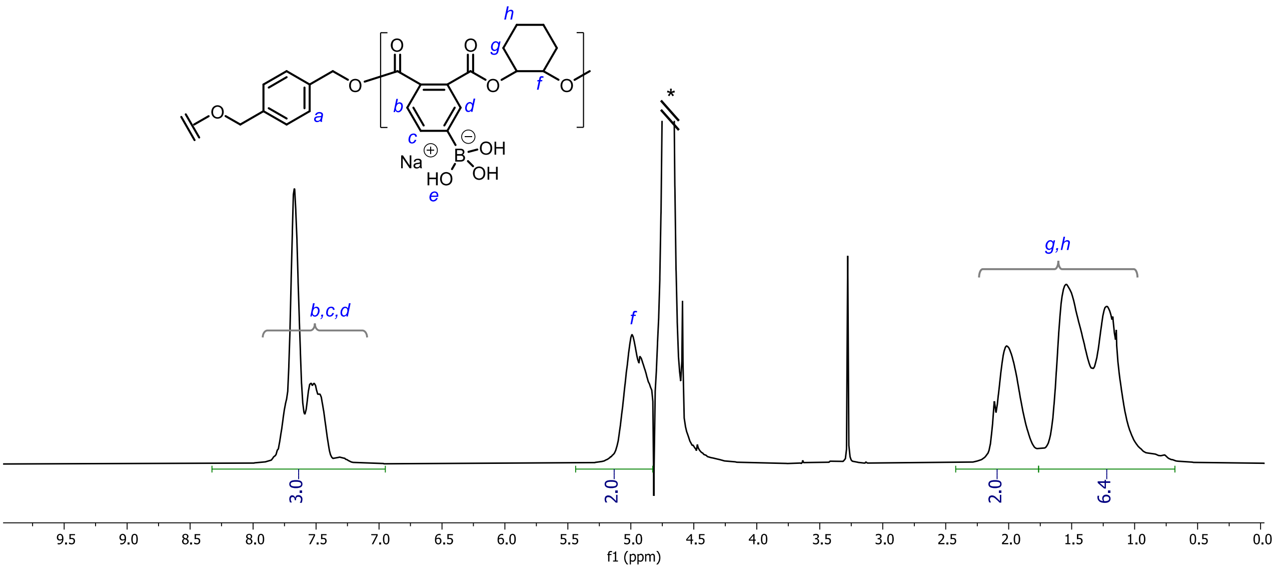

Figure S79. ^{1}H NMR (D_2O, 25 °C, 499.9 MHz] spectrum of *in-situ* generated P[B(OH)$_3$-PA/CHO]. [* = D_2O]. (B–O*H* signal not detected)

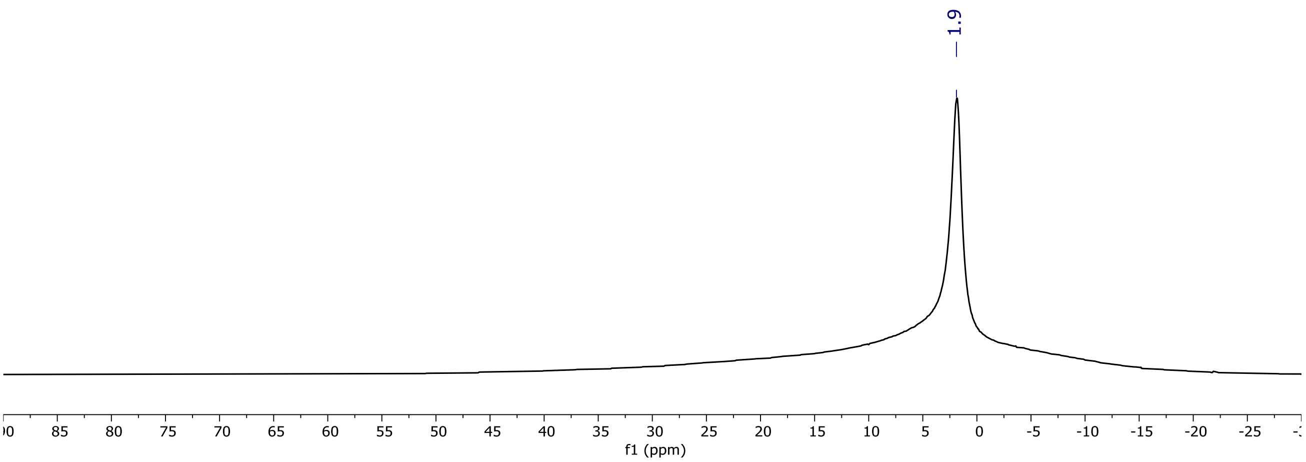

Figure S80. $^{11}B\{^1H\}$ NMR (D_2O, 25 °C, 160.4 MHz) spectrum of *in-situ* generated P[B(OH)$_3$-PA/CHO].

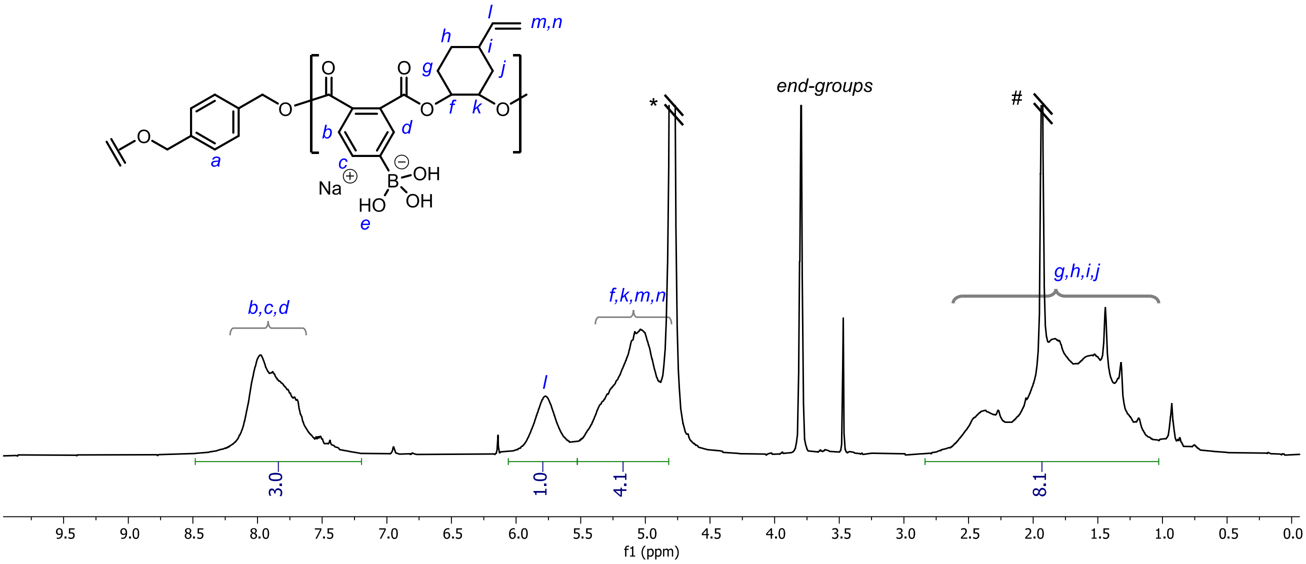

Figure S81. ^1H NMR (D_2O, 25 °C, 499.9 MHz] spectrum of *in-situ* generated P[B(OH)$_3$-PA/vCHO]. [* = D_2O; # = residual acetone]. (B–O*H* signal not detected)

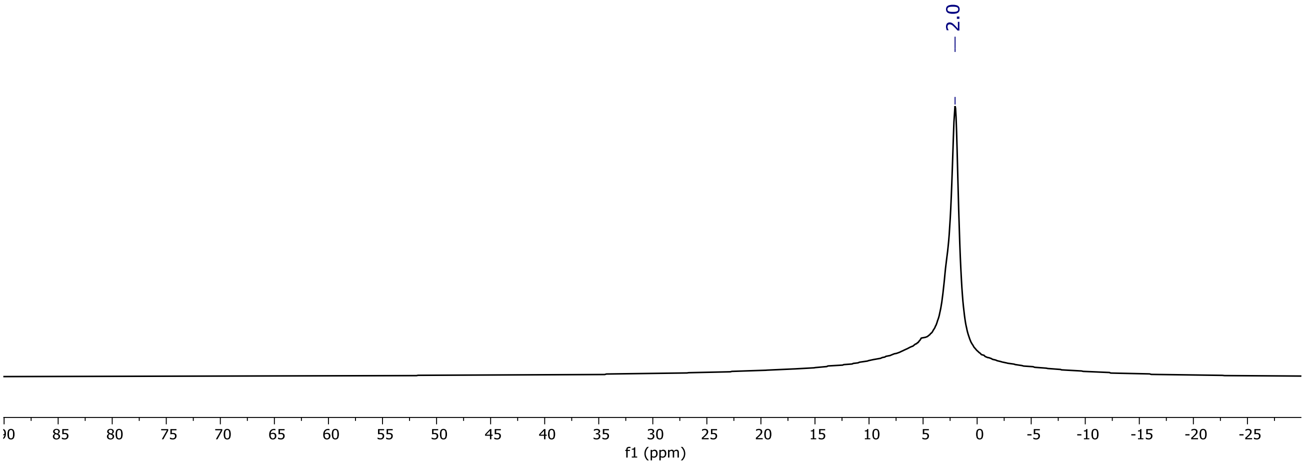

Figure S82. $^{11}B\{^1H\}$ NMR (D_2O, 25 °C, 160.4 MHz) spectrum of *in-situ* generated P[B(OH)$_3$-PA/vCHO].

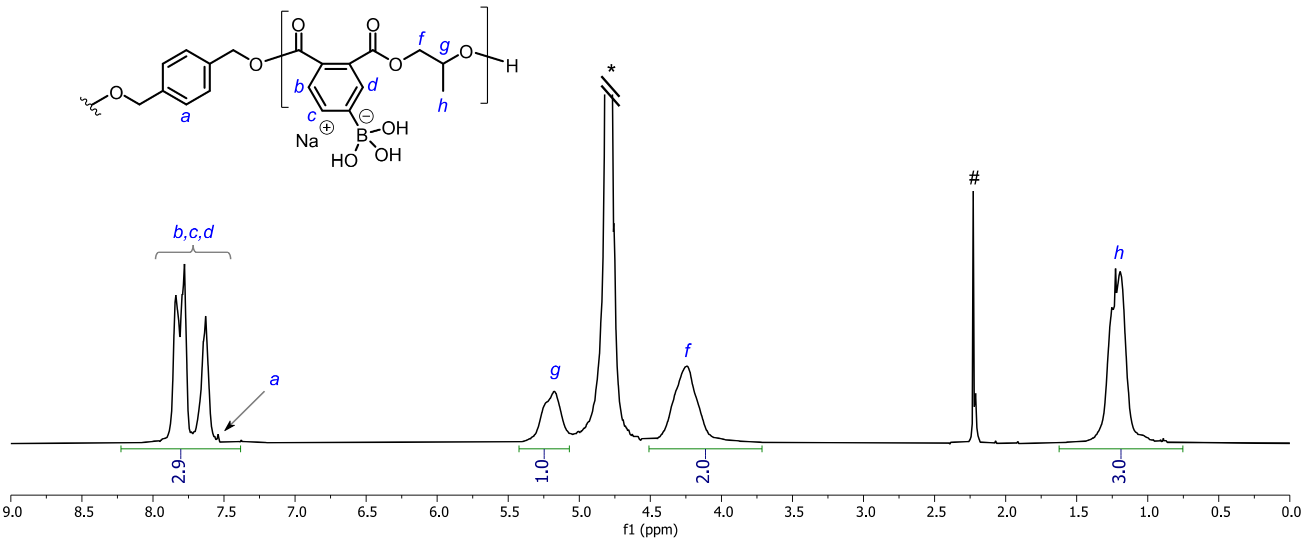

Figure S83. ^{1}H NMR (D_2O, 25 °C, 499.9 MHz] spectrum of *in-situ* generated P[B(OH)$_3$-PA/PO]. [* = D_2O; # = residual acetone]. (B–O*H* signal not detected)

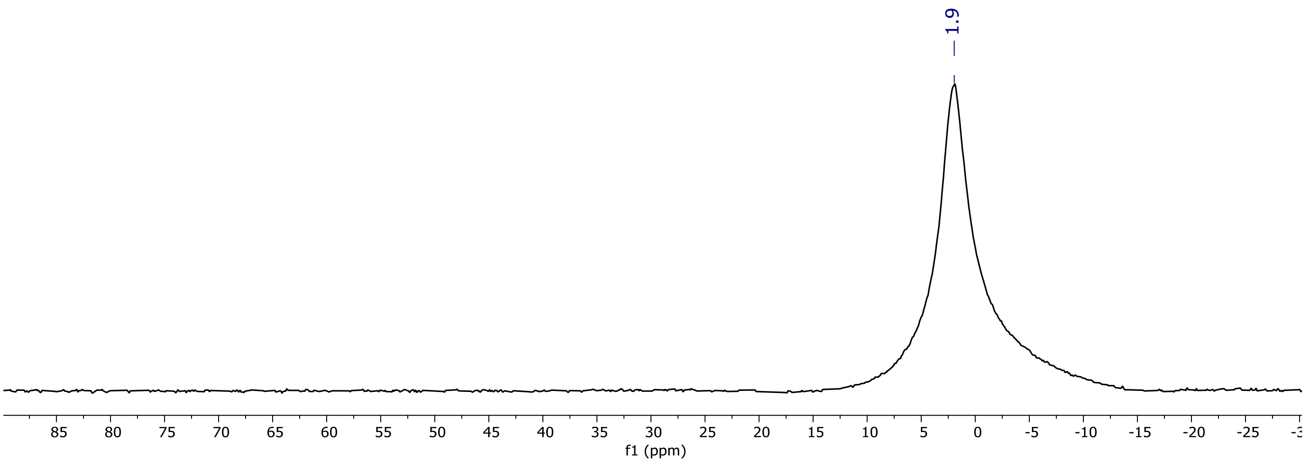

Figure S84. ^{11}B{^{1}H} NMR (D_2O, 25 °C, 160.4 MHz) spectrum of *in-situ* generated P[B(OH)$_3$-PA/PO].

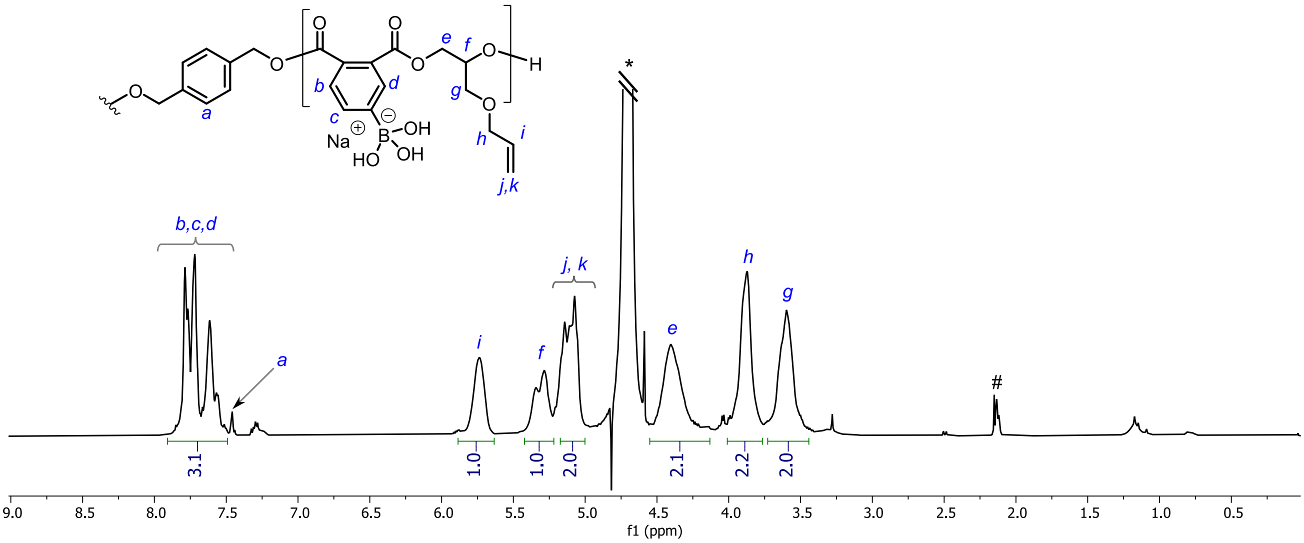

Figure S85. ^{1}H NMR (D_2O, 25 °C, 499.9 MHz] spectrum of *in-situ* generated P[B(OH)$_3$-PA/AGE]. [* = D_2O; # = residual acetone]. (B–O*H* signal not detected)

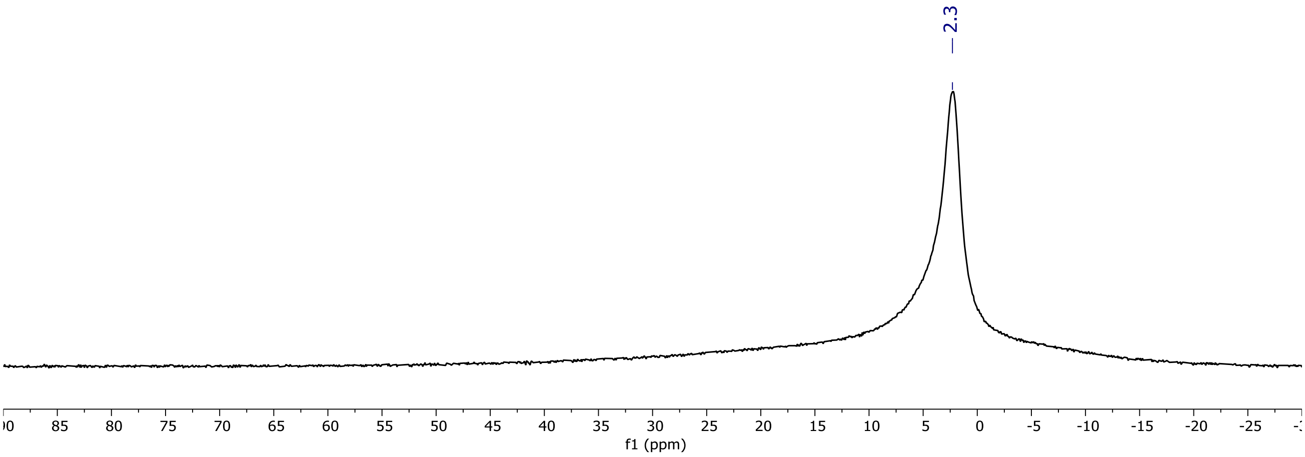

Figure S86. $^{11}B\{^1H\}$ NMR (D_2O, 25 °C, 160.4 MHz) spectrum of *in-situ* generated P[B(OH)$_3$-PA/AGE].

7.2. Hydrolytic Stability in Basic Aqueous Solution

In an Eppendorf tube, a dry sample of P[B(OH)$_2$-PA/PO] (~12 mg) was suspended in D_2O (0.5 mL). Then, 1 equiv. of NaOD with respect to boronic acid units was added using a micropipette from a NaOD/D_2O solution (40 wt%). The mixture was vortexed until a clear solution was obtained, and the resulting solution was analysed by NMR spectroscopy. The reaction was left standing at room temperature (25 °C) and monitored at regular intervals.

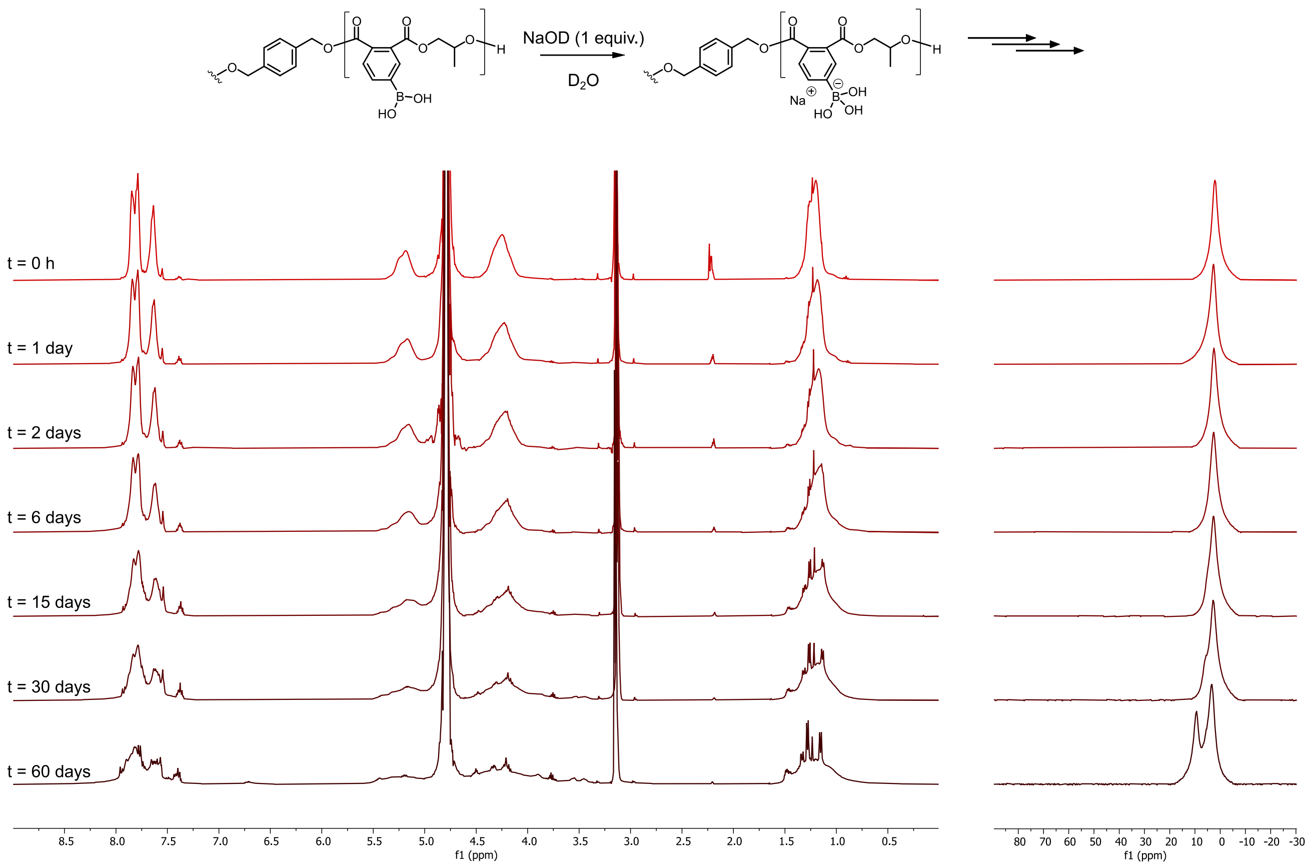

Figure S87. Evolution over time of the ^{1}H NMR (left) and ^{11}B NMR spectra in D_2O (25 °C) of P[B(OH)$_3$-PA/PO]. After 6 days, shaper peaks due to degradation products appear in the ^{1}H NMR spectra.

In an Eppendorf tube, a dry sample of P[B(OH)$_2$-PA/PO] (~12mg) was suspended in D_2O (0.5 mL). Then, 1 equiv. of NaOD with respect to boronic acid units was added using a micropipette from a NaOD/D_2O solution (40 wt%). The mixture was vortexed until a clear solution was obtained, after which an aliquot (250 µL) was immediately quenched over THF (1.0 mL) containing benzoic acid (20 mg/mL) and neopentyl glycol (40 mg/mL). The solution was then filtered and analysed by GPC. The remaining P[B(OH)$_3$-PA/PO] solution (250 µL) was left to stand at room temperature for 4 days, after which it was treated in a similar manner and analysed by GPC.

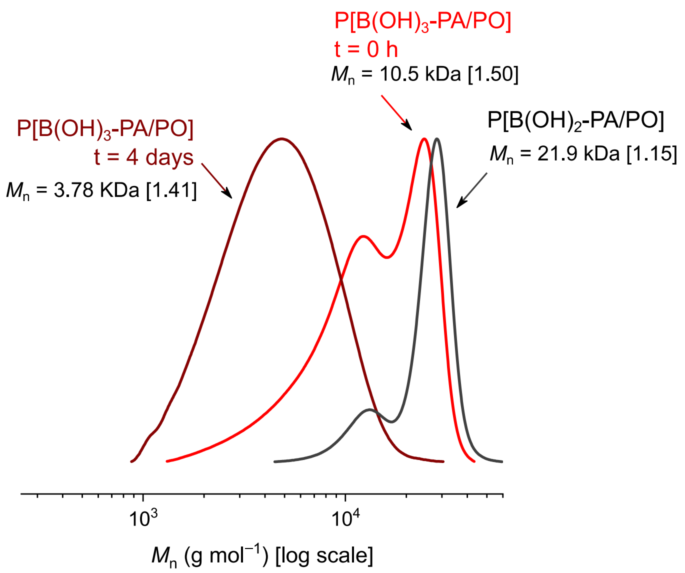

Figure S88. Comparison of normalized GPC traces (RI detector) of boronic acid-polyester P[B(OH)$_3$-PA/PO] (black), and anionic borate-polyester P[B(OH)$_3$-PA/PO]: immediately after dissolution with NaOD and neutralized with benzoic acid (red), and 4 days after dissolution with NaOD (maroon). The slight bimodality in the starting GPC trace of P[B(OH)$_2$-PA/PO] is attributed to residual mono- and di-functional protic impurities which results in competitive initiation with the BDM.

7.3. Monitoring of polymer degradation by NMR spectroscopy

In an Eppendorf tube, a dry sample of polyester P[B(OH)$_2$-PA/PO] (~12 mg, 0.05 mmol of boronic acid units) was suspended in D_2O (0.5 mL) containing dimethylsulfone (2 mg, 25 µmol) as an internal standard. Then, 1 equiv. of NaOD with respect to boronic acid units was added with a micropipette from a NaOD/D_2O solution (40 wt%, 3.5 µL, 0.05 mmol). The mixture was subsequently vortexed, at room temperature, until a clear solution was obtained. At this point, the polymer solution was transferred to an NMR tube and ^{1}H and ^{11}B NMR spectra were recorded at 300 K (time 0). The sample was removed from the spectrometer, a second equiv. of NaOD was added to it, and the sealed tube was shaken vigorously, at room temperature. The NMR tube was immediately returned to the NMR spectrometer and ^{1}H NMR spectra were taken at regular intervals to monitor polymer degradation, at 300 K. Once the reaction was complete, a sample was diluted in distilled H_2O and analysed by LC-MS.

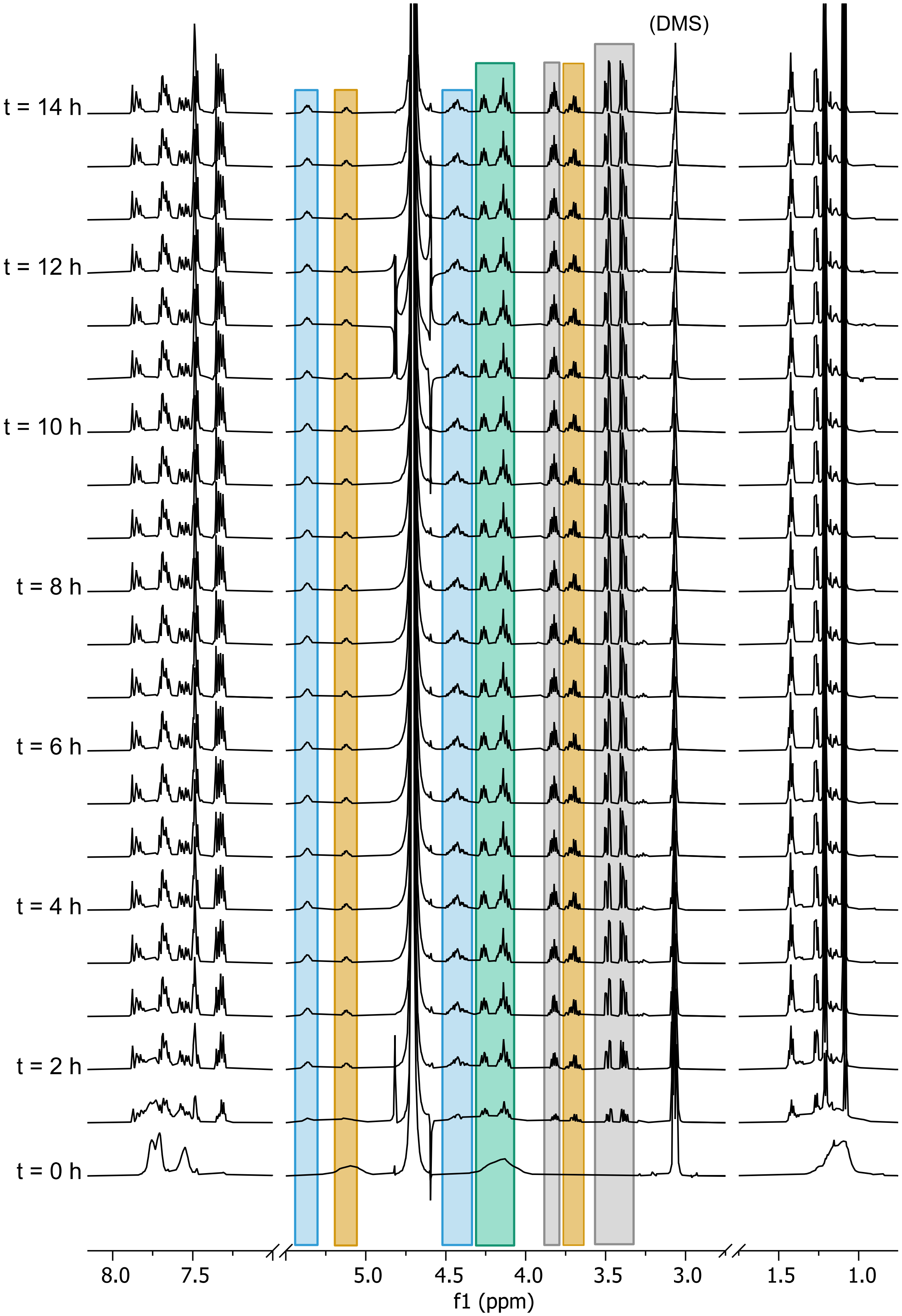

Figure S89. Evolution over time of the ^{1}H NMR spectra in D_2O (300 K) of *in-situ* generated P[B(OH)$_3$-PA/PO] after the addition of one additional equivalent of NaOD, at room temperature.

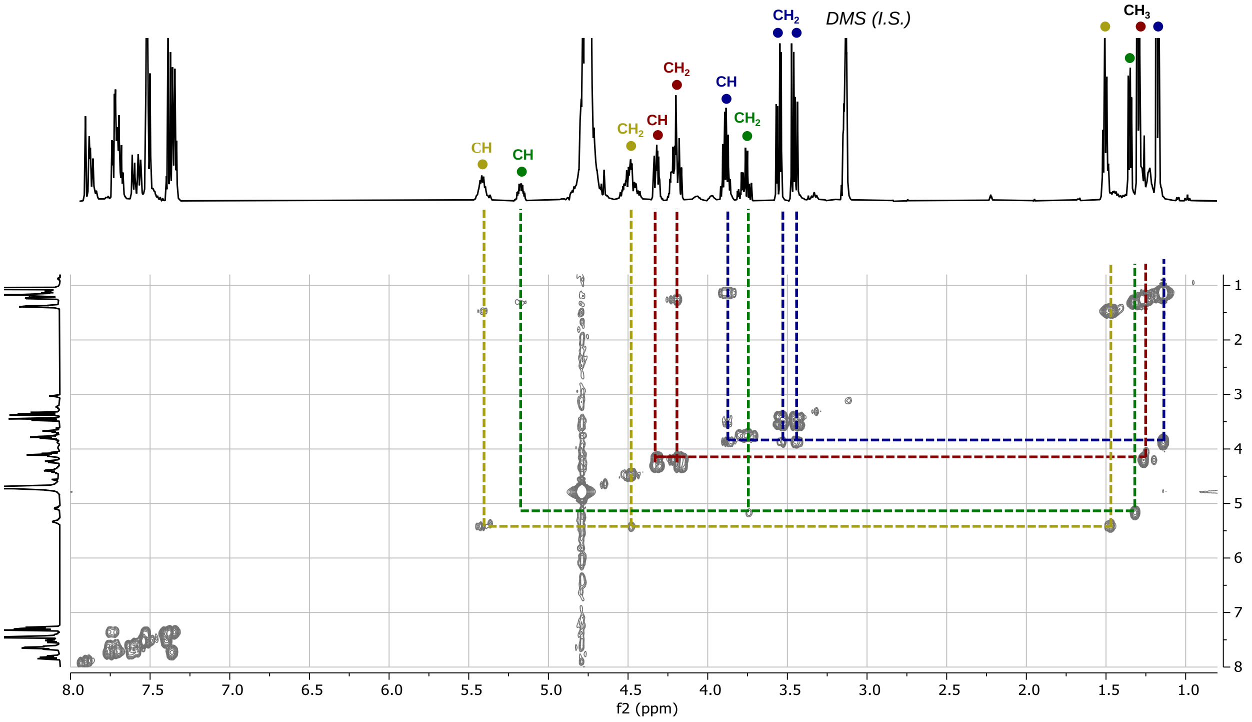

Figure S90. 2D ^{1}H-^{1}H COSY NMR spectrum in D_2O (300 K) of degradation products obtained from the hydrolysis of P[B(OH)$_3$-PA/PO].

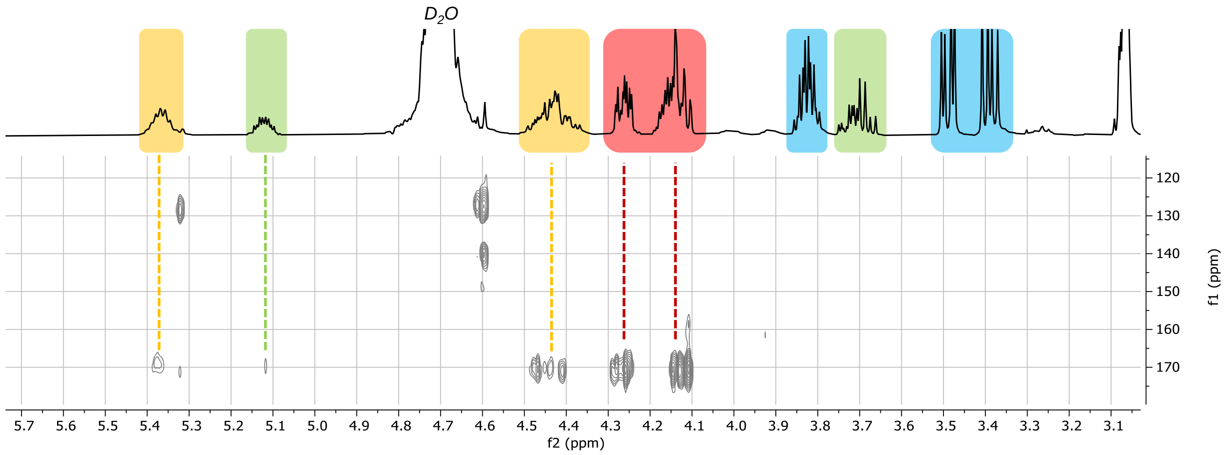

Figure S91. 2D ^{1}H-^{13}C HMBC NMR spectrum (long J coupling 2.5 Hz) in D_2O of degradation products obtained from the hydrolysis of P[B(OH)$_3$-PA/PO].

7.4. Liquid Chromatography-Mass Spectrometry of Polymer Degradation Products

An NMR sample containing degradation products (Figures S84-S86) in D_2O was diluted in HPLC grade water, for analysis by liquid chromatography-mass spectrometry (LC-MS). Mode = negative electron-spray ionization, formic acid (HCOONa) used to facilitate ionization.

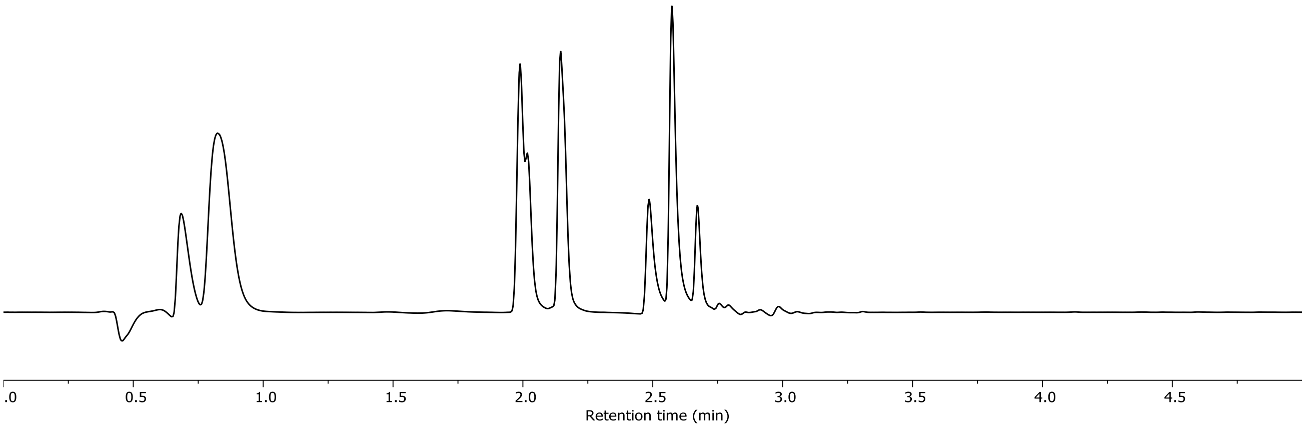

Figure S92. LC-MS chromatogram of hydrolysed P[B(OH)$_2$-PA/PO].

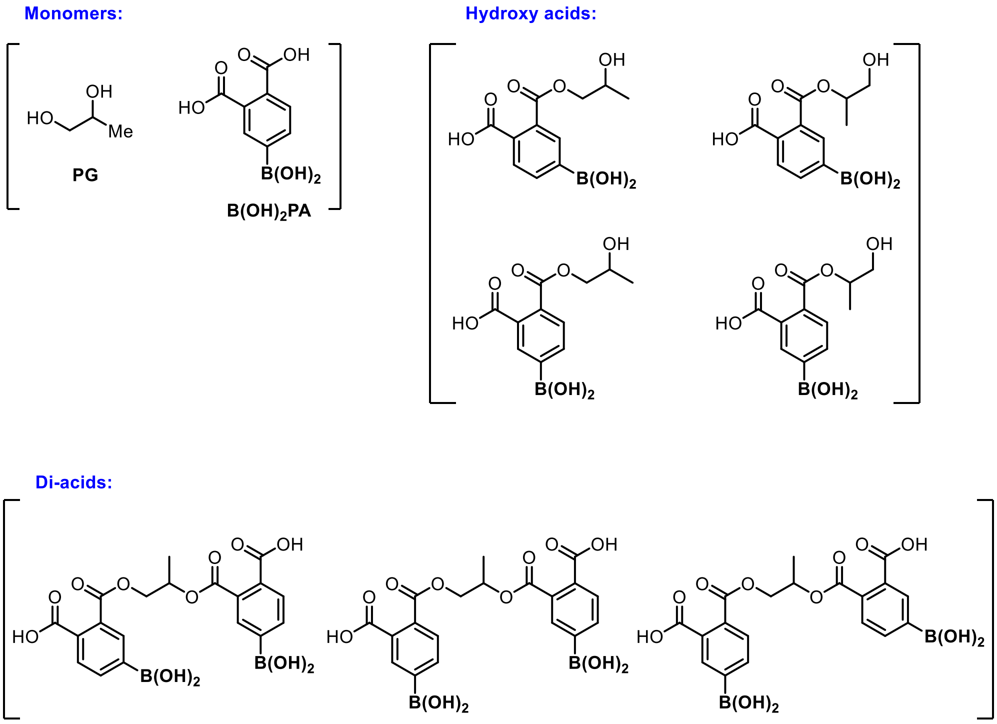

Figure S93. Chemical structures of the different hydrolysis products detected from P[B(OH)$_2$-PA/PO].

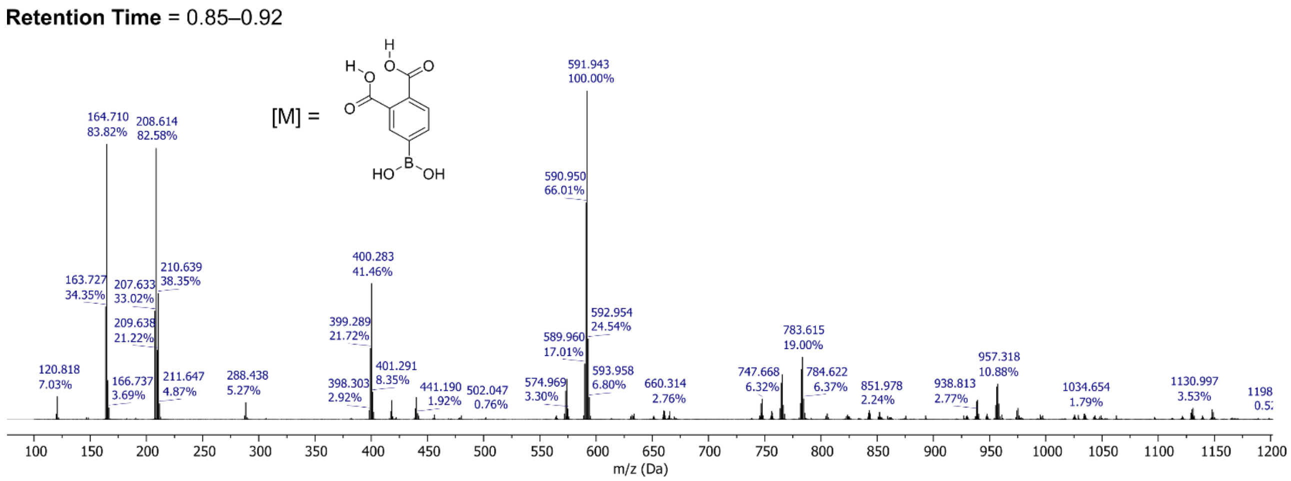

Figure S94. Negative ESI-MS spectrum corresponding to the species present in the peak of the degradation products chromatogram at 0.85–0.92 minutes.

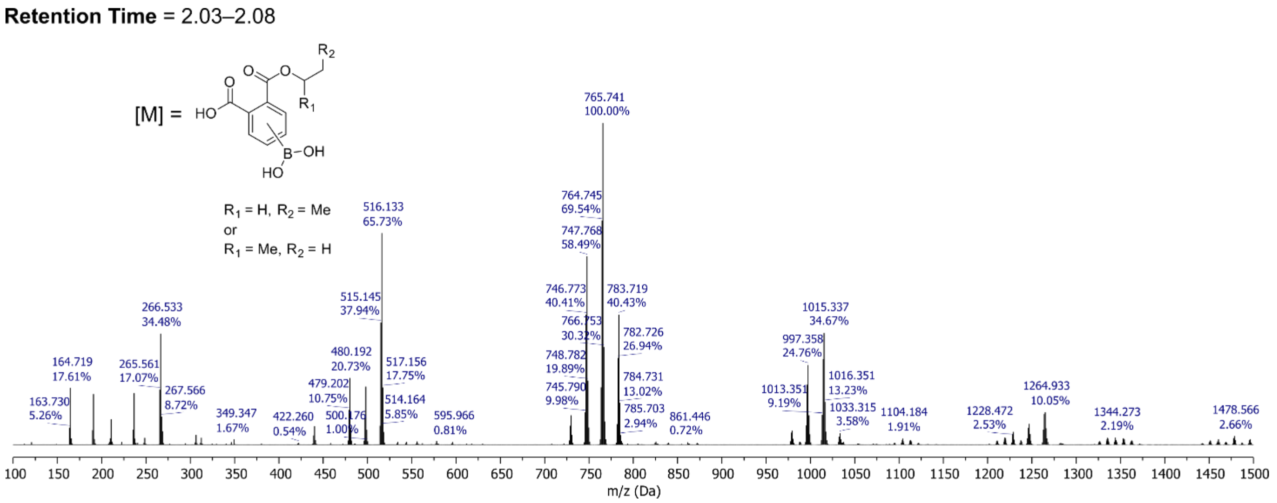

Figure S95. Negative ESI-MS spectrum corresponding to the species present in the peak of the degradation products chromatogram at 2.03–2.08 minutes.

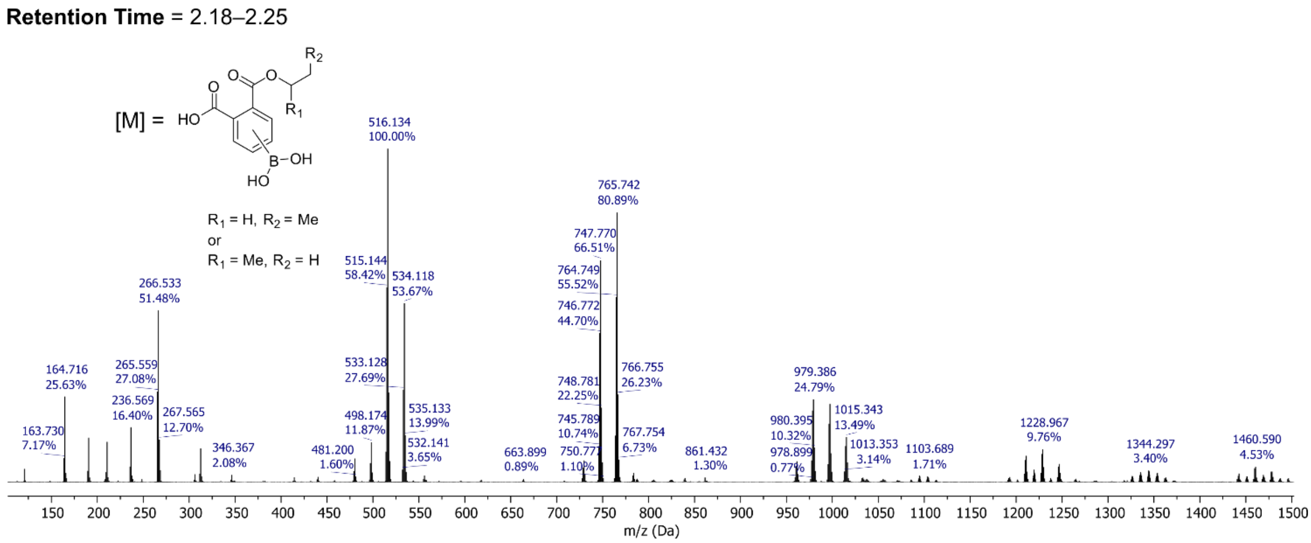

Figure S96. Negative ESI-MS spectrum corresponding to the species present in the peak of the degradation products chromatogram at 2.18–2.25 minutes.

Retention Time = 2.54

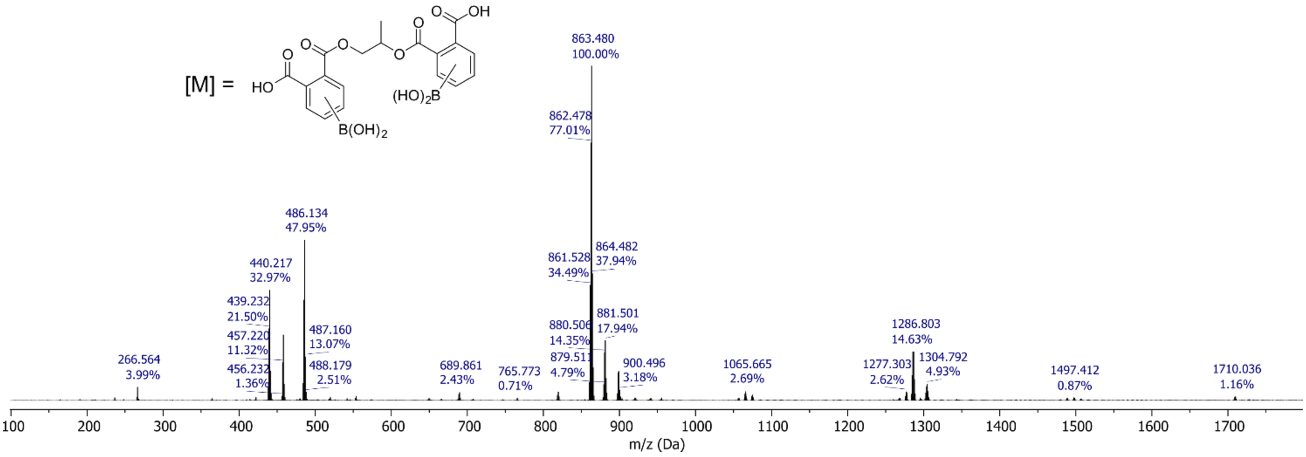

Figure S97. Negative ESI-MS spectrum corresponding to the species present in the peak of the degradation products chromatogram at 2.54 minutes.

Retention Time = 2.68

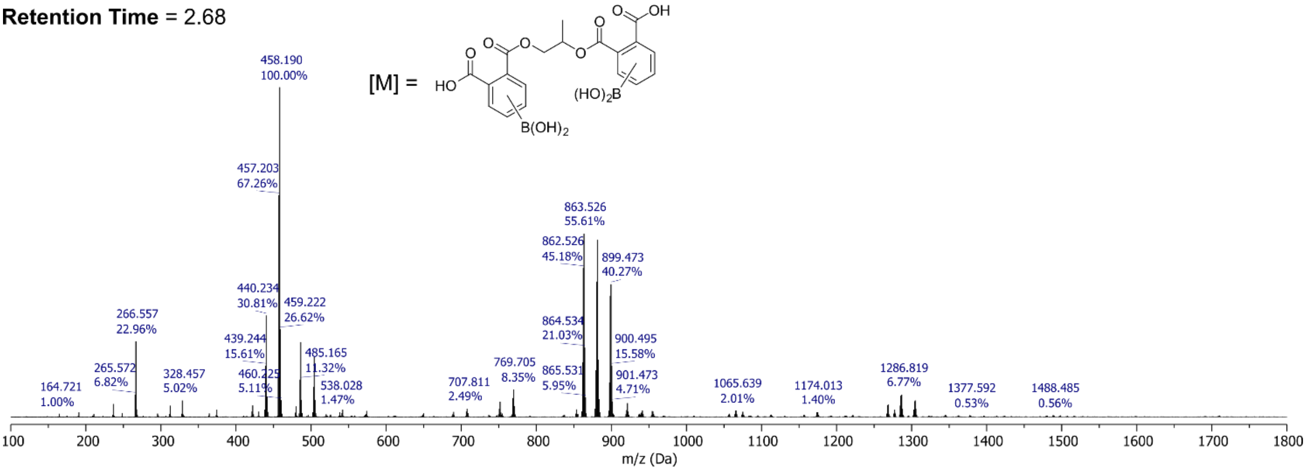

Figure S98. Negative ESI-MS spectrum corresponding to the species present in the peak of the degradation products chromatogram at 2.68 minutes.

Retention Time = 2.73

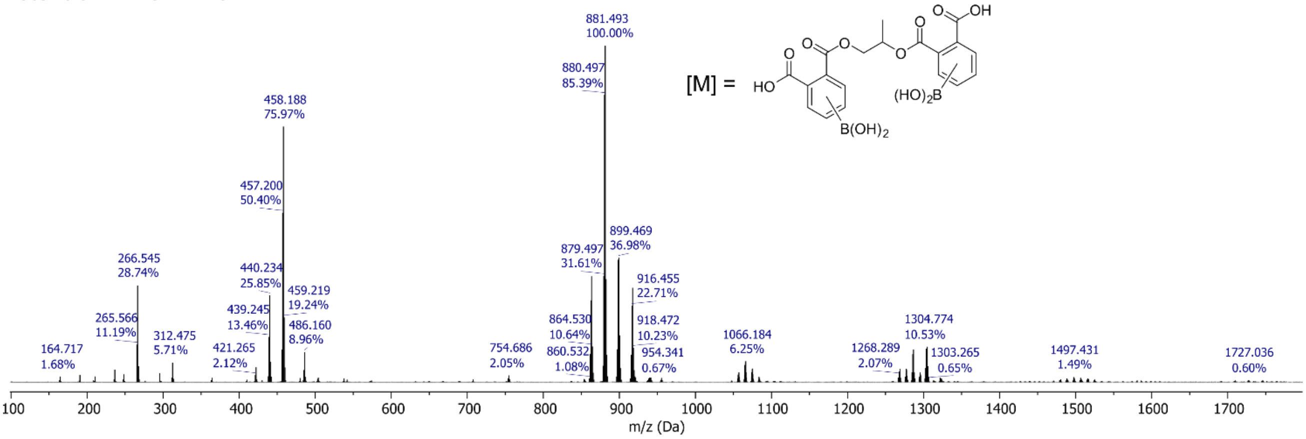

Figure S99. Negative ESI-MS spectrum corresponding to the species present in the peak of the degradation products chromatogram at 2.73 minutes.

Table S4. Proposed ESI-MS assignment of P[B(OH)$_2$-PA/PO] degradation products in alkaline media.

Proposed Structure [M]	Proposed Species	Theo. m/z	Obs. m/z (relat. int. %)	Error (%)	R.T. (min)
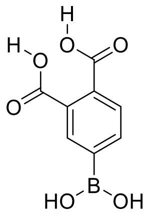	$[M–H–B(OH)_2]^-$	164.010	164.710 (84)	−0.41	0.85–0.92
	$[M–H]^-$	209.025	208.614 (83)	+0.19	
	$[2M–2H–H_2O]^-$	400.040	400.283 (42)	−0.06	
	**$[3M–3H–2H_2O]^-$**	592.063	591.943 (100)	+0.02	
	$[4M–4H–4H_2O]^-$	764.060	765.641 (14)	−0.21	
	$[4M–4H–3H_2O]^-$	782.070	783.615 (19)	−0.20	
	$[5M–5H–5H_2O]^-$	955.075	957.318 (11)	−0.23	
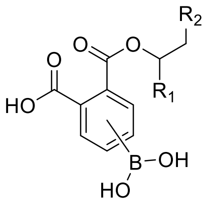 R_1 = H, R_2 = Me or R_1 = Me, R_2 = H isomers A/B	$[M–H–CH_2O]^-$	237.056	236.568 (16)	+0.21	2.03–2.08
	$[M–H]^-$	267.067	266.533 (34)	+0.20	
	$[2M–2H–3H_2O]^-$	480.103	480.192 (21)	−0.02	
	$[2M–2H–2H_2O]^-$	498.113	498.170 (18)	−0.01	
	$[2M–2H–1H_2O]^-$	516.124	516.133 (66)	−0.01	
	$[3M–3H–4H_2O]^-$	729.160	729.794 (9)	−0.09	
	$[3M–3H–3H_2O]^-$	747.171	747.768 (58)	−0.08	
	**$[3M–3H–2H_2O]^-$**	765.181	765.741 (100)	−0.07	
	$[3M–3H–1H_2O]^-$	783.192	783.719 (40)	−0.07	
	$[4M–4H–4H_2O]^-$	996.228	997.358 (25)	+0.11	
	$[4M–4H–3H_2O]^-$	1014.238	1015.337 (35)	+0.11	
	$[5M–5H–4H_2O]^-$	1263.295	1264.933 (10)	−0.13	
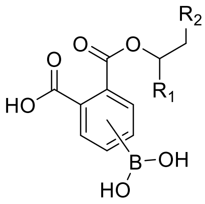 R_1 = H, R_2 = Me or R_1 = Me, R_2 = H isomers C/D	$[M–H–CH_2O]^-$	237.056	236.569 (16)	+0.21	2.18–2.25
	$[M–H]^-$	267.067	266.533 (51)	+0.20	
	$[2M–2H–3H_2O]^-$	480.103	480.201 (7)	−0.02	
	$[2M–2H–2H_2O]^-$	498.113	498.174 (12)	−0.01	
	**$[2M–2H–1H_2O]^-$**	516.124	516.134 (100)	−0.01	
	$[2M–2H]^-$	534.135	534.118 (54)	> 0.01	
	$[3M–3H–4H_2O]^-$	729.160	729.797 (6)	−0.09	
	$[3M–3H–3H_2O]^-$	747.171	747.770 (67)	−0.08	
	$[3M–3H–2H_2O]^-$	765.181	765.742 (81)	−0.07	
	$[4M–4H–5H_2O]^-$	978.217	979.386 (25)	−0.12	
	$[4M–4H–4H_2O]^-$	996.228	997.359 (23)	+0.11	
	$[4M–4H–3H_2O]^-$	1014.238	1015.343 (13)	+0.11	
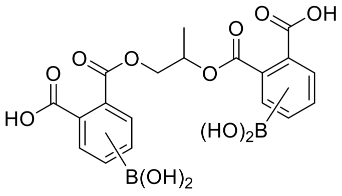 isomer A	$[M–H–H_2O]^-$	441.080	440.217 (33)	+0.19	2.54
	$[M–H]^-$	459.090	458.201 (20)	+0.19	
	**$[2M–2H–3H_2O]^-$**	864.149	863.480 (100)	+0.08	
	$[2M–2H–2H_2O]^-$	882.160	881.501 (18)	+0.08	
	$[2M–2H–1H_2O]^-$	900.170	899.474 (9)	+0.08	
	$[3M–3H–3H_2O]^-$	1290.212	1286.803 (15)	+0.26	
	$[3M–3H–2H_2O]^-$	1308.222	1304.792 (5)	+0.26	
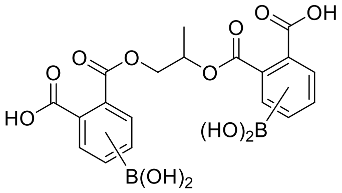 isomer B	$[M–H–H_2O]^-$	441.080	440.234 (31)	+0.19	2.68
	**$[M–H]^-$**	459.090	458.190 (100)	+0.19	
	$[2M–2H–3H_2O]^-$	864.149	863.526 (56)	+0.08	
	$[2M–2H–2H_2O]^-$	882.160	881.498 (54)	+0.08	
	$[2M–2H–1H_2O]^-$	900.170	899.473 (40)	+0.08	
	$[3M–3H–3H_2O]^-$	1290.212	1286.819 (7)	+0.26	

	[3M–3H–2H_2O]$^-$	1308.222	1304.793 (5)	+0.26	
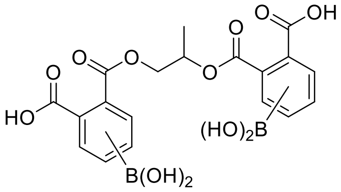 isomer C	[M–H–H_2O]$^-$	441.080	440.234 (26)	+0.19	2.73
	[M–H]$^-$	459.090	458.188 (76)	+0.19	
	[2M–2H–3H_2O]$^-$	864.149	863.523 (31)	+0.08	
	**[2M–2H–2H_2O]$^-$**	882.160	881.493 (100)	+0.08	
	[2M–2H–1H_2O]$^-$	900.170	899.469 (37)	+0.08	
	[2M–2H]$^-$	918.181	917.445 (28)	+0.08	
	[3M–3H–3H_2O]$^-$	1290.212	1286.789 (10)	+0.26	
	[3M–3H–2H_2O]$^-$	1308.222	1304.774 (11)	+0.26	

7.5. Proposed Polymer Hydrolysis Mechanism

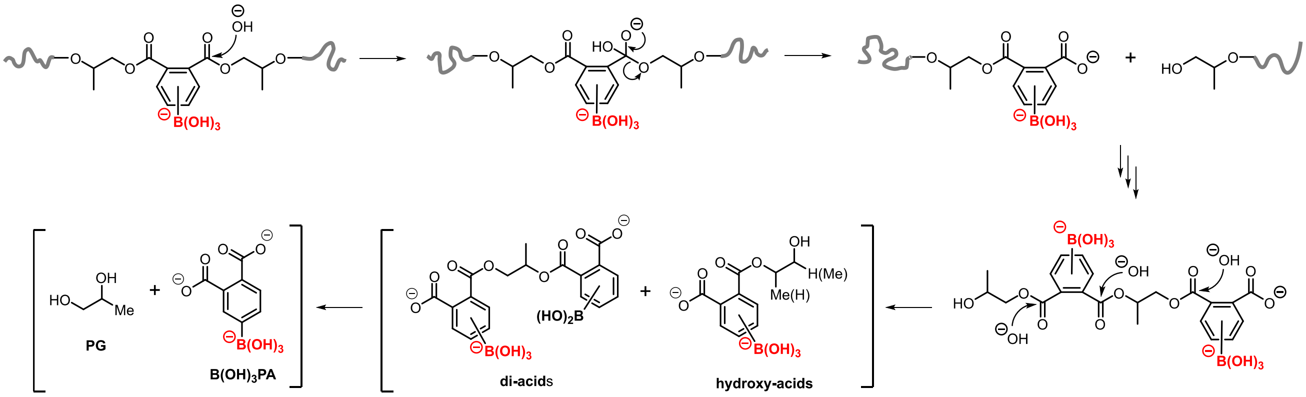

Scheme S1. Proposed polymer hydrolysis mechanism for P[B(OH)$_2$-PA/PO] at high pH.

8. Potentiometric Titration of Dimethyl Phthalate Boronic Acid, [$B(OH)_2$-DMP]

The potentiometric titration of dimethyl phthalate boronic acid, [$B(OH)_2$-DMP], was performed using an automatic titrator system (T5, Mettler Toledo) equipped with a pH electrode (glass electrode DGi115-SC, Mettler Toledo). A three-point calibration of the electrode was carried out using buffer solutions (pH = 4.00, 7.00, and 10.00, Alfa Aesar). Each titration run consisted of $B(OH)_2$-DMP (30 mL of a 0.7 mg·mL^{-1}) in decarbonized Mili-Q water (150 mM, NaCl) titrated with freshly prepared NaOH solution (0.01 N, 150 mM NaCl). The titrant was added with dynamic control (0.2–0.005 mL), at 12-120 s intervals, when the drift equilibrium reached the rate of 0.25 mV/s. Gran plots were constructed from the titration data ($V_b \cdot 10^{-pH}$ vs V_b, where V_b is the volume of NaOH titrant added), and the linear region is fitted to a straight line according to the equation,

$$K_a(V_e - V_b) = 10^{-pH} \cdot V_b$$

where K_a is the dissociation constant of $B(OH)_2$-DMP and V_e·is the equivalence volume. The pK_a of $B(OH)_2$-DMP obtained from the slope of the linear fit ($-K_a$) was determined as **6.96**.

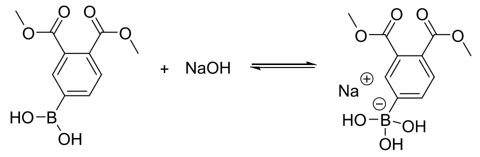

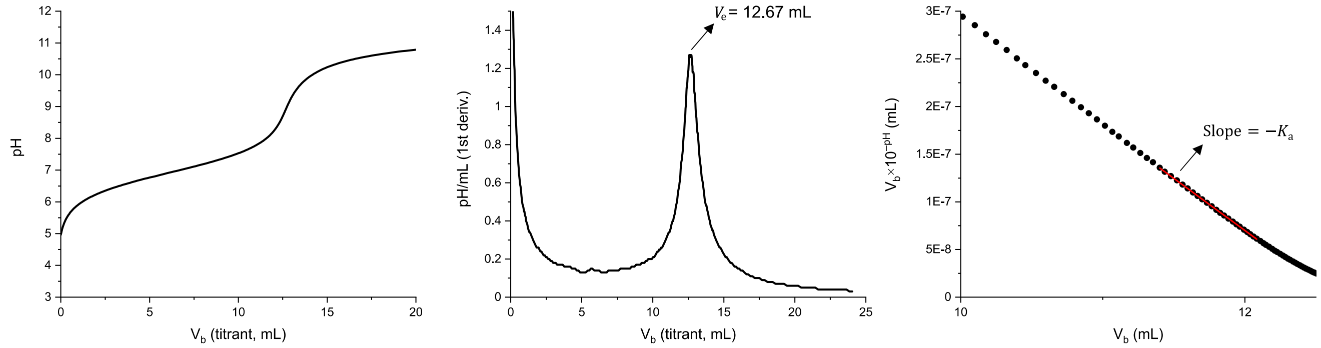

Figure S100. Summary of titration data and Gran plot of $B(OH)_2$-DMP (0.7 mg·mL^{-1}, 150 mM NaCl) with 0.01 N NaOH solution (150 mM NaCl).

9. References

(1) (a) Sulley, G. S.; Gregory, G. L.; Chen, T. T. D.; Peña Carrodeguas, L.; Trott, G.; Santmarti, A.; Lee, K.-Y.; Terrill, N. J.; Williams, C. K. Switchable Catalysis Improves the Properties of CO2-Derived Polymers: Poly(cyclohexene carbonate-b-ε-decalactone-b-cyclohexene carbonate) Adhesives, Elastomers, and Toughened Plastics. *J. Am. Chem. Soc.* **2020**, *142* (9), 4367-4378. DOI: 10.1021/jacs.9b13106. (b) Chen, T. T. D.; Carrodeguas, L. P.; Sulley, G. S.; Gregory, G. L.; Williams, C. K. Bio-based and Degradable Block Polyester Pressure-Sensitive Adhesives. *Angew. Chem. Int. Ed.* **2020**, *59* (52), 23450-23455. DOI: 10.1002/anie.202006807.

(2) Diment, W. T.; Williams, C. K. Chain end-group selectivity using an organometallic Al(iii)/K(i) ring-opening copolymerization catalyst delivers high molar mass, monodisperse polyesters. *Chem. Sci.* **2022**, *13* (29), 8543-8549. DOI: 10.1039/d2sc02752f.

(3) Flores, J. R.; Castruita-De León, G.; Turlakov, G.; Arias, E.; Moggio, I.; Montemayor, S. M.; Torres, R.; Ledezma, R.; Ziolo, R. F.; González-Torres, J. Dual Emission of meso-Phenyleneethynylene–BODIPY Oligomers: Synthesis, Photophysics, and Theoretical Optoelectronic Study. *Chem. Eur. J.* **2020**, *27* (7), 2493-2505. DOI: 10.1002/chem.202004481.

(4) Li, H.; Luo, H.; Zhao, J.; Zhang, G. Well-Defined and Structurally Diverse Aromatic Alternating Polyesters Synthesized by Simple Phosphazene Catalysis. *Macromolecules* **2018**, *51* (6), 2247-2257. DOI: 10.1021/acs.macromol.8b00159.

(5) Si, G.; Zhang, L.; Han, B.; Duan, Z.; Li, B.; Dong, J.; Li, X.; Liu, B. Novel chromium complexes with a [OSSO]-type bis(phenolato) dianionic ligand mediate the alternating ring-opening copolymerization of epoxides and phthalic anhydride. *Polym. Chem.* **2015**, *6* (35), 6372-6377. DOI: 10.1039/c5py01040c.
